# Associations of mortality with own blood pressure using son’s blood pressure as an instrumental variable

**DOI:** 10.1038/s41598-019-45391-w

**Published:** 2019-06-20

**Authors:** David Carslake, Abigail Fraser, Margaret T. May, Tom Palmer, Karri Silventoinen, Per Tynelius, Debbie A. Lawlor, George Davey Smith

**Affiliations:** 1 MRC Integrative Epidemiology Unit at the University of Bristol, Bristol, UK; 2 Population Health Sciences, Bristol Medical School, Bristol, UK; 3 Department of Mathematics and Statistics, University of Lancaster, Lancaster, UK; 4 Population Research Unit, Department of Social Research, University of Helsinki, Helsinki, Finland; 5 Department of Public Health Sciences, Karolinska Institute, Stockholm, Sweden

**Keywords:** Risk factors, Epidemiology

## Abstract

High systolic blood pressure (SBP) causes cardiovascular disease (CVD) and is associated with mortality from other causes, but conventional multivariably-adjusted results may be confounded. Here we used a son’s SBP (>1 million Swedish men) as an instrumental variable for parental SBP and examined associations with parents’ cause-specific mortality, avoiding reverse causation. The hazard ratio for CVD mortality per SD (10.80 mmHg) of SBP was 1.49 (95% CI: 1.43, 1.56); SBP was positively associated with coronary heart disease and stroke. SBP was also associated positively with all-cause, diabetes and kidney cancer mortality, and negatively with external causes. Negative associations with respiratory-related mortality were probably confounded by smoking. Hazard ratios for other causes were imprecise or null. Diastolic blood pressure gave similar results to SBP. CVD hazard ratios were intermediate between those from conventional multivariable studies and Mendelian randomization and stronger than those from clinical trials, approximately consistent with an effect of exposure duration on effect sizes. Plots of parental mortality against offspring SBP were approximately linear, supporting calls for lower SBP targets. Results suggest that conventional multivariable analyses of mortality and SBP are not substantially confounded by reverse causation and confirm positive effects of SBP on all-cause, CVD and diabetes mortality.

## Introduction

Higher blood pressure (BP) resulted in an estimated 10 million deaths and 212 million disability-adjusted life years globally in 2015^1^. Randomised control trial (RCT) and Mendelian randomization (MR) analyses show that higher BP causes mortality from cardiovascular diseases (CVD) including coronary heart disease (CHD) and stroke^2–6^, supporting the results of numerous prospective cohort studies^7–12^. Higher BP has also been associated with mortality from external causes (accidents, homicides and suicides)^13^, renal disease^14^, all-site cancer^15–17^ and with several site-specific cancers^18–23^, including a strong positive association with kidney cancer^15,22,24–26^.

It is not clear whether these non-CVD outcomes are causally affected by BP or whether there is some confounding factor that affects both BP and survival. Potential confounding factors include aspects of behaviour, socioeconomic circumstances and the presence of pre-existing undiagnosed disease (sometimes called reverse causation). Reduced BP will only result in improved survival if the association between them is causal. Limited MR results suggest that higher BP is causally related to the risk of type 2 diabetes^27^, but not kidney disease^6^.

Current advice in the UK is to reduce the BP of hypertensive patients to a target of 140/90 mmHg in adults under 80 years old, and to 150/90 mmHg in older adults^28,29^. However, two major meta-analyses of prospective studies of BP and CVD^7,11^ have suggested that further reductions, to at least 115/75 mmHg, are beneficial to health and the apparent thresholds found in some prospective studies may have been due to confounding by pre-existing disease^30,31^. RCTs of antihypertensive drugs^32–35^ rarely include those with lower baseline BP, but mostly indicate that reduction to below 140/90 mmHg would reduce CVD mortality, with one study^2^ suggesting an advantage in reductions to 110/70 mmHg. BP target values are the subject of ongoing debate^2,32,36–38^.

Here, we estimate the effect of BP on all-cause and cause-specific mortality using a son’s BP as an instrumental variable (IV) for their parent’s BP. An IV is a variable that is associated with the exposure of interest, but not with the factors confounding the association between the exposure and outcome. It can therefore be used to estimate causal effects in the presence of confounding. In MR, the IV is a genetic variant associated with the person’s (usually lifelong) exposure. We use the BP of a person’s son in the same way. A son’s BP is correlated with their parent’s BP but is unlikely to be affected by the parent’s existing, but undiagnosed, illness. This approach may also avoid some socioeconomic and behavioural confounding, but not all, since socioeconomic position and health-related behaviours track across generations. We compare our results with those from RCTs of anti-hypertensive treatments, MR and conventional multivariable analyses of prospective cohorts to explore evidence for different potential causes of bias and duration of exposure to higher BP across these studies. We also examine the shape of the association between son’s BP and parental mortality for any evidence of a threshold.

## Results

### Description of study sample by quintiles of blood pressure

The standard deviations (SD) of son’s BP (used to convert all BP to SD units) were 10.80 mmHg for systolic blood pressure (SBP) and 9.22 mmHg for diastolic blood pressure (DBP). In the main dataset, sons with higher SBP or DBP had greater height and BMI and were less likely to smoke (Supplementary Tables S2 and S3). Their fathers also had greater height, BMI and BP and were less likely to smoke. Their mothers and fathers were born earlier, had lower educational and occupation socio-economic position (SEP) and were older when the son was born. Results within the data subset with data on father’s BP were similar, except that sons with higher SBP (but not DBP) had parents of higher SEP (Supplementary Tables S4 and S5). Characteristics of sons, fathers and mothers varied similarly with father’s BP (Supplementary Tables S6 and S7).

### Two-sample IV estimates for parental mortality

In the main dataset, there were 152,575 maternal deaths and 281,489 paternal deaths from all causes. The mean differences in father’s BP per SD of son’s BP (used as denominators in the IV ratios) are shown in Table 1. These associations were strong, approximately linear (Supplementary Fig. S2) and changed little with adjustment for the father’s SEP and/or the son’s BMI (Table 1).

**Table 1:** Mean differences in father’s blood pressure per standard deviation (SD) of son’s blood pressure.

Blood pressure	Regression of father’s blood pressure (SD) against son’s blood pressure (SD)			
	Adjustment	Mean difference (95% CI)	F-statistic	R^2^
SBP	None	0.130 (0.122, 0.137)	1121.8	0.0166
SBP	Father’s SEP	0.131 (0.123, 0.138)	145.5	0.0193
SBP	Father’s SEP, son’s BMI	0.128 (0.120, 0.135)	134.4	0.0199
DBP	None	0.060 (0.053, 0.067)	278.8	0.0042
DBP	Father’s SEP	0.060 (0.053, 0.067)	36.1	0.0049
DBP	Father’s SEP, son’s BMI	0.059 (0.052, 0.066)	38.1	0.0057

Systolic blood pressure (SBP) and diastolic blood pressure (DBP) were each pre-adjusted for regional patterns, secular trends and age at examination. Blood pressure in fathers and sons was analysed in SD units (10.80 mmHg SBP and 9.22 mmHg DBP). Mean differences were obtained from linear regression and provide the denominators for the ratio method instrumental variable estimates. N = 66,567. F-statistics and R^2^ are provided as measures of instrument strength.

**Figure 1:**
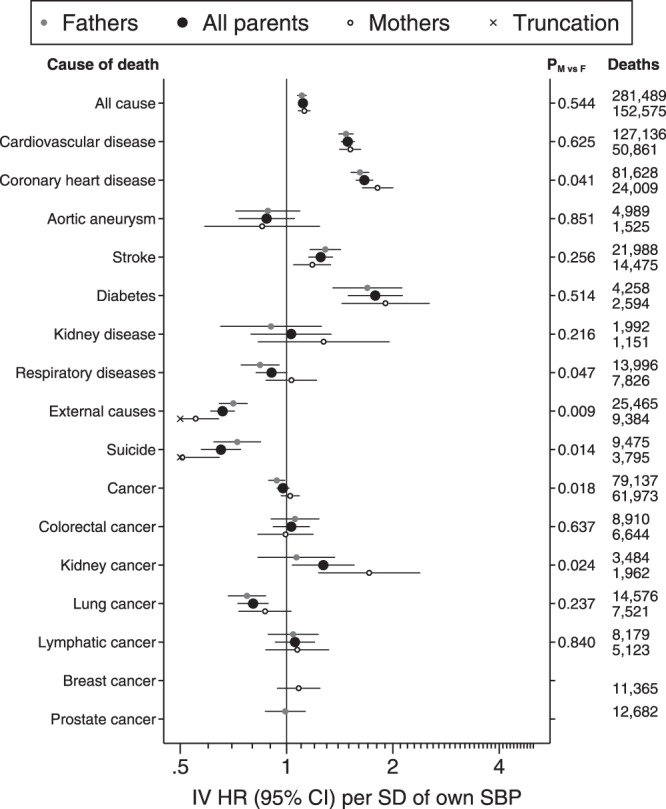
Adjusted hazard ratios (HR) for parental mortality per standard deviation (SD) of own SBP, using son’s SBP as an instrumental variable (IV). SBP was pre-adjusted for regional patterns, secular trends and age at examination and its SD was 10.80 mmHg. Cox proportional hazards models with age as the time axis were adjusted for parental sex and for educational and occupational socioeconomic position. Robust standard errors were clustered by the son’s identity. P_M vs F_ was derived from a Z-test of an additional interaction term between parental sex and son’s SBP. Two-sample IV estimates were made using the ratio method. Mothers and fathers were also modelled separately, without the robust standard errors or the adjustment for parental sex. Plotted data are tabulated in Supplementary Table S8. N = 1,002,031 mothers and 986,075 fathers at risk of mortality.

Two-sample IV analysis suggested a modest association between SBP and all-cause mortality (HR per SD: 1.11, 95% CI: 1.09, 1.14) which did not differ between mothers and fathers (Fig. 1, Supplementary Table S8). Reasonably well-powered results for CVD, CHD, stroke and diabetes suggested stronger positive associations. SBP had a strong negative association with external causes and suicide mortality and weaker negative associations with mortality from respiratory diseases (fathers only) and lung cancer. Very strong positive associations with cancers of the oesophagus and kidney were apparent only in mothers and a strong positive association with thyroid cancer was imprecisely estimated (Supplementary Table S14). Precision for many site-specific cancers was too low to allow confident interpretation.

**Figure 2:**
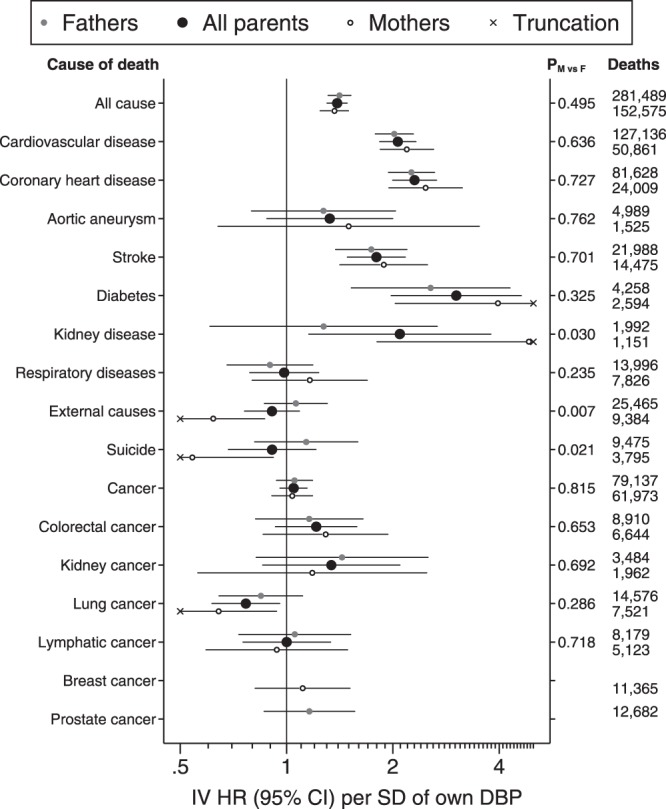
Adjusted hazard ratios (HR) for parental mortality per standard deviation (SD) of own DBP, using son’s DBP as an instrumental variable (IV). DBP was pre-adjusted for regional patterns, secular trends and age at examination and its SD was 9.22 mmHg. Cox proportional hazards models with age as the time axis were adjusted for parental sex and for educational and occupational socioeconomic position. Robust standard errors were clustered by the son’s identity. P_M vs F_ was derived from a Z-test of an additional interaction term between parental sex and son’s DBP. Two-sample IV estimates were made using the ratio method. Mothers and fathers were also modelled separately, without the robust standard errors or the adjustment for parental sex. Plotted data are tabulated in Supplementary Table S9. N = 1,002,031 mothers and 986,075 fathers at risk of mortality.

Two-sample IV analyses of DBP (Fig. 2, Supplementary Table S9) showed a positive association with all-cause mortality (HR per SD: 1.39, 95% CI: 1.30, 1.48) that was stronger than that observed for SBP. Positive associations with CVD, CHD, stroke and diabetes were also greater. Unlike for SBP, there was a strong but imprecise positive association with kidney disease, which was most apparent in mothers. The negative association of SBP with external causes and suicide mortality was also apparent with DBP among mothers, but not fathers, and there was no apparent association of DBP with respiratory diseases. There was again a negative association with lung cancer and a positive association with kidney cancer which matched those of SBP, albeit with very low power in the latter. Positive associations of DBP with liver cancer and malignant melanoma (Supplementary Table S15) were only very weakly repeated for SBP (Supplementary Table S14). A sex-specific association with oesophageal cancer (positive in mothers, negative in fathers) was apparent for both SBP and DBP, albeit with very low precision in both cases.

Two-sample IV analyses that were not adjusted for SEP (Supplementary Tables S10 and S11) gave slightly higher hazard ratios (i.e. stronger positive ones and weaker negative ones) but the differences were not sufficient to change interpretation materially. Additional adjustment for son’s BMI substantially attenuated IV hazard ratios for diabetes mortality (e.g. from 1.78 (95% CI: 1.49, 2.13) to 1.29 (95% CI: 1.08, 1.55) for SBP), but had a relatively small effect for other causes of death (Supplementary Tables S12 and S13). There was strong evidence suggesting that the positive association between a son’s BP and parental all-cause mortality (except for DBP and fathers) increased with the parent’s age, but hazard ratios from follow-up split at 60 years old (Supplementary Tables S18 and S19) suggested that the magnitude of the difference may have been minor. For most specific causes of mortality, there was no strong evidence of non-proportional hazards.

### One-sample IV estimates for parental mortality

Hazard ratios from one-sample IV analyses in the subset with data on father’s BP (Tables 2 and 3, Supplementary Tables S16 and S17) were broadly similar to the corresponding estimates from two-sample IV analyses (Figs 1 and 2, Supplementary Tables S8 and S9), but were imprecise and hazard ratios could not confidently be distinguished from the null. Conventional multivariable analyses of father’s BP in this subset were a little more precise and showed positive associations of SBP and DBP with CVD, CHD and stroke mortality. The results were weakly suggestive of negative associations with mortality from external causes, suicide and lung cancer. Conventional multivariable hazard ratios for all-cause mortality were close to the null, with reasonable precision (HR per SD: 1.03, 95% CI: 0.99, 1.07 for SBP and HR per SD: 1.01, 95% CI: 0.97, 1.06 for DBP). Estimation of hazard ratios by conventional multivariable and IV methods in the same dataset allows their comparison by Durbin-Wu-Hausman test. There was no evidence that the two methods gave different estimates, regardless of the outcome, exposure or adjustment, although this should be interpreted in the context of the very low precision of the one-sample IV estimates.

**Table 2:** Adjusted hazard ratios (HR) for paternal mortality (i) per standard deviation (SD) of own systolic blood pressure (SBP) and (ii) per SD of own SBP, using son’s SBP as an instrumental variable (IV) within the subset having data on own SBP.

Cause of death	Deaths	HR (95% CI) per SD of own SBP	IV HR (95% CI) per SD of own SBP	P_own vs IV_
All cause	2,332	1.03 (0.99, 1.07)	1.01 (0.74, 1.37)	0.873
Cardiovascular disease	423	1.21 (1.11, 1.33)	1.34 (0.65, 2.77)	0.779
Coronary heart disease	235	1.23 (1.09, 1.39)	1.91 (0.72, 5.04)	0.373
Stroke	86	1.21 (0.99, 1.48)	1.92 (0.39, 9.56)	0.568
External causes	1,065	0.97 (0.92, 1.03)	0.94 (0.60, 1.48)	0.884
Suicide	466	0.95 (0.87, 1.04)	0.87 (0.44, 1.72)	0.780
Cancer	428	1.04 (0.95, 1.15)	1.00 (0.48, 2.05)	0.898
Brain cancer	61	1.15 (0.91, 1.47)	0.31 (0.05, 2.09)	0.174
Lung cancer	59	0.85 (0.66, 1.10)	1.24 (0.18, 8.66)	0.698
Lymphatic cancer	64	1.02 (0.80, 1.30)	0.35 (0.05, 2.24)	0.255

SBP was pre-adjusted for regional patterns, secular trends and age at examination and its SD was 10.80 mmHg. Cox proportional hazards models with age as the time axis were adjusted for educational and occupational socioeconomic position. One-sample IV estimates were made using the ratio method. P_own vs IV_ was derived from Durbin-Wu-Hausman tests comparing the two HR. N = 66,567 fathers at risk of mortality. Rarer causes of death (<50 deaths in the data subset) are omitted.

**Table 3:** Adjusted hazard ratios (HR) for paternal mortality (i) per standard deviation (SD) of own diastolic blood pressure (DBP) and (ii) per SD of own DBP, using son’s DBP as an instrumental variable (IV) within the subset having data on own DBP.

Cause of death	Deaths	HR (95% CI) per SD of own DBP	IV HR (95% CI) per SD of own DBP	P_own vs IV_
All cause	2,332	1.01 (0.97, 1.06)	0.69 (0.35, 1.35)	0.264
Cardiovascular disease	423	1.11 (1.00, 1.23)	1.23 (0.25, 5.95)	0.901
Coronary heart disease	235	1.13 (0.98, 1.29)	2.70 (0.32, 22.66)	0.419
Stroke	86	1.14 (0.91, 1.43)	3.62 (0.11, 122.51)	0.520
External causes	1,065	0.97 (0.91, 1.04)	0.69 (0.25, 1.87)	0.496
Suicide	466	0.98 (0.89, 1.08)	0.90 (0.20, 4.05)	0.910
Cancer	428	1.03 (0.93, 1.14)	0.59 (0.12, 2.83)	0.486
Brain cancer	61	0.97 (0.74, 1.27)	0.29 (0.00, 18.49)	0.569
Lung cancer	59	0.84 (0.64, 1.11)	1.11 (0.02, 76.55)	0.900
Lymphatic cancer	64	0.94 (0.73, 1.22)	0.06 (0.00, 3.21)	0.171

DBP was pre-adjusted for regional patterns, secular trends and age at examination and its SD was 9.22 mmHg. Cox proportional hazards models with age as the time axis were adjusted for educational and occupational socioeconomic position. One-sample IV estimates were made using the ratio method. P_own vs IV_ was derived from Durbin-Wu-Hausman tests comparing the two HR. N = 66,567 fathers at risk of mortality. Rarer causes of death (<50 deaths in the data subset) are omitted.

### Shape of the associations between son’s blood pressure and parental mortality

**Figure 3:**
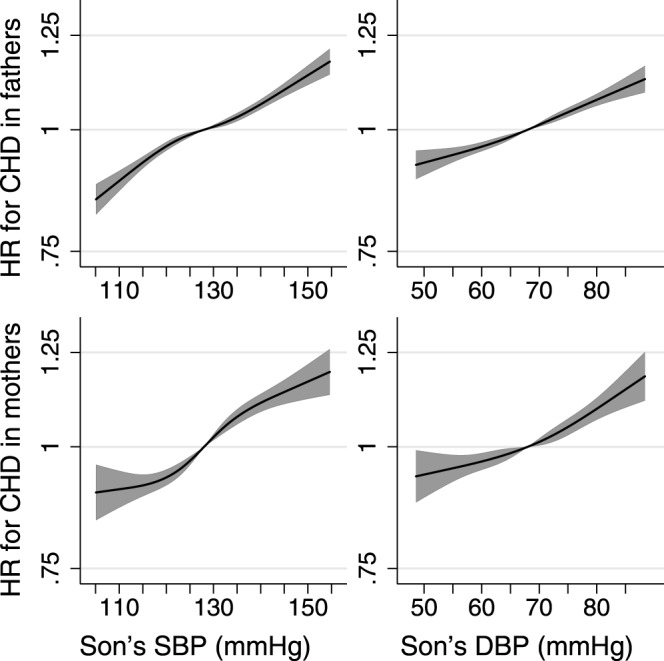
Plots of hazard ratio (HR; relative to the median blood pressure) for parental coronary heart disease (CHD) mortality against a son’s systolic (SBP) or diastolic (DBP) blood pressure. Son’s SBP and DBP were pre-adjusted for regional patterns, secular trends and age at examination. Cox regressions with parental age as the time axis modeled SBP or DBP as cubic splines with 4 knots and were adjusted for the parent’s educational and occupational socioeconomic position. Shaded areas represent 95% confidence intervals. For clarity, plots are truncated at the 1^st^ and 99^th^ percentiles of SBP or DBP.

As well as providing the numerator of the linear IV ratio (Figs 1 and 2, Supplementary Tables S8–S13) the association between son’s BP and parental mortality can be plotted to indicate the likely shape of the association between BP and mortality with some sources of confounding removed (Fig. 3, Supplementary Fig. S3). Paternal mortality from all-causes, CVD, CHD, stroke, external causes and suicide was approximately linear across the observed ranges of son’s SBP and DBP. In mothers, there was some suggestion that the positive associations of son’s SBP with mortality from all causes, CVD, CHD and stroke leveled off below approximately 120 mmHg, and that the association of DBP with diabetes mortality leveled off below about 70 mmHg. However, wide confidence intervals, particularly at lower BP, left some uncertainty in the shape of the associations. Plotted associations for the rarer causes of death were very imprecise.

## Discussion

Using a son’s BP as an IV for his parents’ BP, we confirmed clear positive associations of SBP and DBP with death from all-causes, CVD, CHD, stroke and diabetes. SBP (and to a lesser extent DBP) had strong, negative associations with suicide and external causes of death and weaker negative associations with death from lung cancer and respiratory disease. Several other causes of death, particularly from rarer cancers, showed suggestive associations which were estimated imprecisely and were often inconsistent between men and women, or between SBP and DBP.

### Do hazard ratios using son’s blood pressure as an IV estimate the causal effect of blood pressure on mortality?

IV methods give estimates which reflect the causal effect of the exposure (here, the parent’s own BP) on the outcome (here, the parent’s survival) if some important assumptions are met^39^. In particular, the untestable assumption is made that the instrument is not associated with any unmeasured factors confounding the relationship between the exposure (parents BP) and outcome (mortality). Here this assumption is likely to be violated because a son’s socioeconomic position and characteristics associated with it, such as smoking, are likely to be associated with these same characteristics in the parents.

Although adjustment for the few socioeconomic covariates which were measured did not greatly affect the IV estimates of the association between mortality and BP in parents (compare Supplementary Tables S8 and S9 with Supplementary Tables S10 and S11), we cannot assume that these reflect all unmeasured confounding. In particular, the inverse association of BP with respiratory diseases and lung cancer in our IV analyses is probably due to the association of son’s BP with smoking in the parents. Smoking is strongly associated with increased mortality from these causes and is positively associated between generations. Most studies have found smoking to be associated with reduced BP^40–42^, though it is not clear if this association is causal^43^. The limited dichotomous smoking data available in the present study also suggest a negative association with BP but were too sparse to be used in adjustment. Furthermore, we had no data at all on more detailed measures of smoking (e.g. pack-years or cotinine levels).

When effects of exposure on outcome are estimated in IV analyses, any biases in the association between instrument and outcome due to such confounding will be amplified in inverse proportion to the strength of the instrument-exposure association^44^. An IV analysis may therefore be more biased than the analogous conventional multivariable analysis even if the unmeasured confounder is more closely associated with the exposure than it is with the instrument^45^. Although son’s BP is a stronger instrument than most of the genetic instruments used in MR^46^, it is more likely to be associated with factors confounding the association between BP and mortality. It is also considerably weaker than other offspring characteristics (height, BMI) previously used as IVs for the same characteristic in their parents^47,48^. It therefore seems likely that these IV estimates for BP are subject to at least as much bias from socioeconomic confounding as conventional multivariable estimates are. We therefore present these IV estimates not as unconfounded, but as differently confounded. They may be interpreted alongside estimates from other methods to improve causal inference under the principles of triangulation^49^.

**Figure 4:**
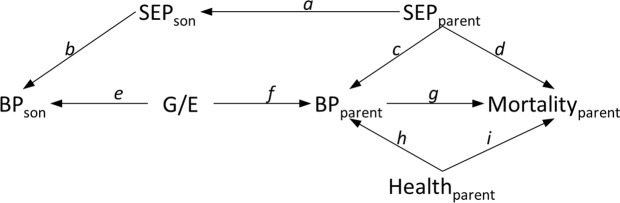
Directed acyclic graph illustrating potential confounders of the effect of blood pressure (BP) on mortality, when a son’s BP is used as an instrument. A conventional estimate of *g* (the effect of the exposure on the outcome) may be biased due to confounding via pathways such as *cd* and *hi*. An IV estimate of the same effect may be biased by confounding via pathway *bad*, and any such bias is magnified by the reciprocal of the association *ef* + *bac* between instrument and exposure. The IV estimate of *g* is likely to be biased by socio-economic position (SEP; and other environmental or behavioural factors such as smoking behaviour which are associated between generations) but we argue that reverse causation (i.e. confounding by the parent’s health) is unlikely to bias the IV estimate. An unbiased instrumental variables analysis also requires that there be no pathway from instrument to outcome (except via the exposure) and that the association between instrument and exposure is non-null. In the present case, a causal effect of son’s blood pressure on parental BP is implausible; we must further assume that there is no causal effect of parental BP on son’s BP and that the common genetic and environmental factors (G/E) causing the instrument and exposure to be associated (*ef*) are distinct from those confounding the exposure and outcome.

While the present estimates may be vulnerable to socioeconomic confounding, reverse causation (i.e. confounding from pre-existing disease) should not be a problem, since a medical condition in the parents is unlikely to have a major effect on their son’s adult BP (Fig. 4). There is evidence^30,50–52^ suggesting reverse causation acting on BP up to three years prior to death, but it is not clear whether chronic, sub-clinical conditions can lower BP earlier than this. Conventional multivariable analyses in which BP is measured at a young age may therefore have limited vulnerability to reverse causation, but we consider offspring BP to be an instrument for lifelong BP in the parents, including in old age when reverse causation is likely to be important in conventional multivariable analyses.

### Comparison with other studies

This study confirms the positive associations of high BP with mortality from CHD and stroke previously found using MR, RCTs and conventional multivariable analyses^2–12^. One previous study^53^, using Norwegian data from the HUNT cohort, has estimated associations between BP and mortality using offspring BP as an IV. In that study, the offspring’s BP was measured at a rather later age (mean 29.5 years compared to 18.3 here) which may account for the greater SD in SBP (16.6 mmHg in men, compared to 10.8 here; SD of DBP was similar in the two studies). Lower power meant that only four causes of death were considered in the earlier study and all four, as in the present study, were positively associated with BP. Considered per SD, they found similar HR for all-cause mortality to the present study, rather lower HR for CVD and CHD mortality, and for stroke mortality the HR per SD of SBP were similar to the present study but those per SD of DBP were rather lower. When the HR per SD of SBP were converted to HR per mmHg, those for CVD and CHD were considerably lower than those in the present study; for example the HR for CHD mortality per 10.8 mmHg SBP was 1.19 (95% CI: 1.10, 1.29) compared to the analogous HR here (Fig. 1, Supplementary Table S8) of 1.66 (95% CI: 1.57, 1.75). These differences may be due to the more comprehensive adjustment possible in the earlier study or to the more advanced age of both offspring at BP measurement and parents during follow-up.

Our results for SBP and CHD may be compared with those of Ference *et al*.^4^, who meta-analysed analogous results from RCTs (relative risk reduction (RRR) per 10 mmHg lower SBP: 17%; 95% CI: 10%, 24%), prospective cohort studies (RRR: 25%; 22%, 29%) and MR (RRR: 46%; 32%, 56%). Our result (RRR per 10 mmHg lower SBP: 37%; 34%, 40%) is intermediate between those for cohort studies and MR. Arguably, the duration of exposure in the present IV study is also intermediate between the values for cohort studies and MR in Ference *et al*., which would be consistent with the suggestion^4^ that effect estimates increase in proportion to the duration of exposure in different study types.

The associations with CHD were a little stronger than most results from conventional multivariable analyses. For example, we found an IV hazard ratio per SD of SBP of 1.66 (95% CI: 1.57, 1.75), compared with 1.39 (1.38, 1.41) in the largest meta-analysis to date^54^ (taking the 60–69 age group which most closely matches the mean age at death in our data, and converting hazard ratios to our units). In contrast, at 1.25 per SD of SBP (95% CI: 1.16, 1.35) our hazard ratio for stroke mortality was rather smaller than that in the same meta-analysis, which was 1.58 (1.54, 1.62). Besides the possible effect of duration of exposure mentioned above, these differences could reflect cause-specific differences in the bias remaining under each methodology or could simply be due to population differences. The hazard ratios found in the meta-analysis above were very similar to those found in an independent meta-analysis of Asia-pacific cohorts^11^, but a more recent study of a single large UK cohort^12^ found much smaller associations, which may be due to the increasing use of antihypertensive medication^50^.

High BP has previously been associated with increased mortality from external causes, including suicide^13^. In contrast, our IV analyses suggested a strong negative association with SBP in both sexes and with DBP in mothers; high BP appeared to be protective against external causes and suicide mortality. A previous conventional multivariable analysis using much of the same data as the present study^10^ found a positive association of DBP (but not SBP) with suicide but negative associations of SBP and DBP with other external causes mortality. Low BP is associated with impaired cognitive performance^55^, anxiety and depression^56^ and fainting^57^, all of which provide plausible causal pathways for a negative association between BP and external causes mortality. Positive associations in previous studies could be due to confounding by psychologic distress, socioeconomic position or alcohol consumption^13^. Some of these sources of confounding might be reduced in the present study and in the previous study of similar data by the young age at which BP was recorded. Alternatively, differences between populations, particularly regarding the age of follow-up, could be responsible for the difference.

It has been suggested^7^ that the proportional effect of high BP on mortality declines with age; we found weak evidence supporting this for CHD and stroke mortality. The increasing HR with age that we found for all-cause mortality were probably due to the decreasing contribution of external causes mortality (against which high BP appears protective in our study) to total mortality as the parents aged.

Previous studies have found an association between high BP and increased incidence of, or mortality from, kidney disease^14,58^. We found a similar association for DBP which was rather stronger in women than in men. The association with SBP, however, was close to the null, albeit with sufficient imprecision that a positive association of similar magnitude could not be ruled out (after conversion to our units, the Asia-Pacific Cohort Studies Collaboration found HR: 1.41, 95% CI: 1.31, 1.53 compared to our IV estimate of HR: 1.03, 95% CI: 0.79, 1.34). The extent to which high BP is a consequence, rather than a cause, of kidney disease remains uncertain^6,59,60^ but reverse causation is largely accounted for in our IV analysis. We also found positive, but rather imprecise, associations of BP with kidney cancer which supports previous studies suggesting that high BP can also cause cancer in the kidneys.

Our IV results for any-site cancer were close to the null, but this appears to have been a consequence of positive and negative associations for several different site-specific cancers cancelling each other out. Power was generally low for site-specific cancers. Those that appeared associated with BP in one sex only, or with only one of SBP and DBP, should be interpreted with particular caution given the number of tests conducted. Our IV results for SBP and DBP suggested a negative association with lung cancer that was consistent between the sexes. By contrast, previous studies^61,62^ have suggested a positive association. As discussed above, our IV estimates of the association between higher BP and lung cancer mortality are probably biased downwards by confounding by smoking, which previous studies adjusted for.

The substantial attenuation of the IV estimates for diabetes when adjusted for son’s BMI suggests that these particularly strong positive associations may have been confounded by BMI, which is a particularly strong risk factor for diabetes^63^ and is associated between generations. Nonetheless, the attenuated HR was similar in magnitude to the positive effect of higher SBP on diabetes risk (approximate odds ratio per 10.80 mmHg: 1.24, 95% CI: 1.11, 1.38) reported by an MR^27^.

### Is there a threshold for the benefits of reduced blood pressure?

If the associations between BP and mortality are taken to be causal, the presence or absence of a threshold below which reductions in BP are no longer beneficial is important for the optimal management of high BP^33,36,38^. Consistent with two major meta-analyses of prospective cohort studies^7,11^, we observed associations between son’s BP and paternal CHD mortality that were approximately log-linear across the observed range of BP. For maternal CHD mortality the confidence intervals at the lower end of the SBP distribution were wide enough to be consistent with a threshold at around 115/60 mmHg, or with an approximately linear association. No other causes of death showed clear evidence of a threshold. A previous study of this population^10^ found that CVD mortality ceased to decline with a subject’s own SBP and DBP below thresholds of about 120 mmHg and 70 mmHg, respectively. In the same study, the association with all-cause mortality reversed at low SBP such that the overall curve was U- or J-shaped. These differences may be attributed to the different patterns of confounding, or to the demographic changes in the population between the two studies. The fathers in our study were older on average than the men in the previous study (50 years old *versus* 42) and external causes mortality, negatively associated with BP in both studies, contributed a smaller proportion (9% *versus* 50%) of the total mortality.

When considering the treatment of high BP, it should be noted that associations between BP and mortality, even if causal, may not apply in the same way to drug-induced changes in BP^37^. Furthermore, reduced BP and increased medication may impact upon quality of life, which becomes increasingly important at older ages.

### Strengths and limitations of the study

The study followed survival among the parents of an almost complete cohort of Swedish men. This gave a very large sample which was highly representative of the recent population of Sweden. Those who do not have sons are the only substantial section of the population missing from the study. Biological mechanisms causally linking BP to mortality are likely to be similar in different populations, but patterns of socioeconomic confounding might be different in populations outside Europe or even Scandinavia (the setting for both studies to date using this method). There was some evidence of digit preference in the BP measurements, which may result in a slight attenuation of the results towards the null. The availability of BP data in a subsample of fathers allowed the use of a son’s BP as an IV and not just as a proxy and was a particularly valuable feature of these data. By using a son’s BP as an IV, we were able to increase the follow-up time and to avoid confounding by pre-existing disease. The two major limitations of the IV approach are the risk of amplifying any remaining confounding and the reduction in power for the same sample size. The subsample of fathers with measured BP, although large by normal epidemiological standards, was not sufficient to make usefully powerful comparisons of the IV and conventional multivariable methods on comparable data. The absence of any BP data for mothers meant that two-sample IV estimates for mothers required the additional assumption that the mother-son association in BP was the same as the father-son association. There is some evidence that this is the case for SBP, but that a son’s DBP may be better associated with his father’s DBP than his mothers^64,65^. When comparing IV estimates for maternal and paternal mortality it should also be noted that they could be differently biased if, for example, a son’s health-related behavior and hence BP are more affected by unhealthy behaviour in the mother than by similarly unhealthy behaviour in the father. The father-son correlation in SBP in the present study was similar to that found in other studies, while the correlation in DBP was rather lower, and less than that typically found elsewhere^64,65^. Besides the amplification of confounding considered above, a weak association between the exposure and the instrument can lead to IV estimates being biased towards the conventional multivariable estimates. The sample size, however, led to reassuringly large F-statistics for the father-son associations.

## Conclusions

Using a son’s BP as an IV for his parents’ BP, we found that son’s SBP and DBP were positively associated with mortality in both parents from all-causes, CVD, CHD, stroke and diabetes. SBP, but not DBP, was inversely associated with mortality from external causes and specifically suicide. These results are approximately consistent with other published studies using subjects’ own BP and suggest that hazard ratios estimated by conventional multivariable methods are not substantially confounded by pre-existing ill-health. The negative associations found with respiratory disease and lung cancer contrast with the positive associations found by most conventional multivariable studies and were probably confounded by smoking. The limited evidence available to us suggests that these IV estimates are at least as vulnerable to socioeconomic and behavioural confounding as conventional multivariable estimates are. Other causal inference methods using observational data, such as MR, may give more reliable estimates of the causal effect of long-term differences in BP. When a son’s BP was plotted as a proxy for his parents’ BP, we found no strong evidence of a threshold below which the positive association between BP and CVD mortality was nullified or reversed.

## Methods

### Data preparation

The Swedish Multi-Generation Register provided the unique national identity numbers and dates of birth of all 1,629,396 males born in Sweden between 1951 and 1980 (Supplementary Fig. S1), and those of their biological parents. The identity numbers of the sons and fathers were linked to records from conscription examinations held between 1969 and 2001. Conscription examinations were compulsory for young Swedish men during this period, except for those with severe handicap or chronic disease. They took place at a mean age of 18.3 years (range 16 to 25, with 91% aged 17 or 18) at one of six regional conscription centres and provided data on examinees’ SBP, DBP, height and weight. Blood pressures were measured after 5–10 minutes’ rest in a supine position, with an appropriately sized cuff at heart level. Manual cuffs were used until 1995 in most examination centres, before replacement with automatic ones. If SBP was less than or equal to 145 mmHg and DBP between 50 and 85 mmHg, a single measurement was made. Otherwise, a second measurement was made and this value was recorded and used for analysis^10^. Data were missing for 17% of examinees, mainly due to accidental loss following administrative changes at the conscription authority. Smoking habits were available for 29,485 examinations, mostly from 1969 or 1970.

The Swedish Cause of Death Register provided the underlying cause for the 281,489 paternal and 152,575 maternal deaths which occurred between 1961 and 2004. These were converted from international classification of diseases (ICD) codes into broad categories, some of which were nested within others (Supplementary Table S1). Emigrants were also identified, allowing the assumption that parents who were not dead or emigrated by 31^st^ December 2004 were still alive at this date. The Swedish Population and Housing Census provided data on parental educational level and occupational status in 1970 and 1990. We took the higher of the 1970 and 1990 values for educational level and classified it into five levels: <9 years; 9–10 years; full secondary education; tertiary education; and missing (2.0% of mothers and 6.1% of fathers). We also classified parents according to five mutually exclusive categories of occupational status: high/intermediate non-manual; lower non-manual; skilled manual; unskilled manual; and other/missing. We used the 1970 value for parents born before 1935 and the 1990 value for parents born later. 27.5% of mothers fell into the other/missing category, comprising 1.9% missing data, 0.8% farmers and 24.9% others (including housewives, students, pensioners and part-time workers). 17.2% of fathers were categorised as other/missing, comprising 4.7% missing, 4.0% farmers and 8.5% others.

To avoid pseudoreplication within families, only one son from each parent was retained in the database. Retention was random except that whenever possible, the same son was retained for both his parents. Our main analyses were thus conducted on 986,075 father-son pairs and 1,002,031 mother-son pairs. In 66,567 father-son pairs, the father’s BP (measured at his own conscription examination at age 18) was also available. This subset of the data was used to quantify the intergenerational association of BP and for other analyses requiring data on the father’s BP (Supplementary Fig. S1).

### Statistical analysis

Analyses described below were conducted separately for SBP and DBP (which we refer to collectively as BP). Before all further analysis, son’s BP was adjusted for regional patterns, secular trends and the effect of age at examination by taking residuals from a regression of son’s BP on regional conscription centre (categorical variable with six levels) and cubic splines of age at examination and date of birth (7 knots at percentiles of 2.5, 18.3, 34.2, 50, 65.8, 81.7 and 97.5)^66^. A similar adjustment was applied to father’s BP, where available, and both were divided by the SD of adjusted BP in sons. Sons’ and parents’ characteristics were summarised by quintiles of son’s adjusted BP and linear or logistic regression was used to examine associations as appropriate.

We estimated the effect of parents’ own BP on their mortality using the ratio method in two-sample instrumental variable (IV) analyses^39^. If certain assumptions are met, IV methods allow the estimation of causal effects free from confounding and reverse causation. The instrument was son’s BP. In IV estimates made by the ratio method the numerator is the association between the outcome and the instrument and the denominator is the association between the exposure and the instrument. Since BP was not measured in women, IV estimates for mother’s mortality used a denominator based on the father-son association in BP. The denominator was estimated in the subset of data for which the father’s BP was available (N = 66,567; Supplementary Fig. S1). Linear regression provided the mean difference in father’s BP (in SD units) per SD of son’s BP, with and without additional adjustment for the father’s occupational status and educational level, which we refer to collectively as socioeconomic position (SEP). The numerator was estimated in the main dataset in which father’s BP was not required (N = 986,075 father-son and 1,002,031 mother-son pairs; Supplementary Fig. S1). Cox proportional hazards models were used to estimate the natural logarithms of hazard ratios (HR) for all-cause and cause-specific parental mortality per standard deviation (SD) of son’s BP. Initial models analysed parents of both sexes together, with adjustment for parental sex and robust standard errors clustered by the son’s identity. Parental age was used as the time axis and models were run with and without additional adjustment for SEP. Observations were right-censored at the earliest of parent’s date of death or emigration, or on 31^st^ December 2004 (the end of follow-up). They were left-truncated at the latest of the son’s date of birth or 1^st^ January 1961 (the start of follow-up). To test whether the associations with son’s BP differed between mothers and fathers, parent’s sex was further allowed to interact with son’s BP and SEP (if included). Separate analyses were also conducted for mothers and fathers, omitting adjustment for parent’s sex and the clustered robust standard errors. Ratio method IV estimates made using similar adjustment in the numerator and denominator were exponentiated to provide IV estimates of the HR for parental mortality per SD of their own BP. Confidence intervals were calculated using Taylor series expansions^67^. To test the proportional hazards assumption, correlation coefficients between the parent’s age and the Schoenfeld residuals were calculated. Follow up was also split to estimate separate hazard ratios for parents before and after their 60^th^ birthday. To test whether associations were confounded by body mass index (BMI), we conducted a sensitivity analysis in which the IV numerator and denominator were additionally adjusted for son’s BMI (parental BMI was not available in the main dataset).

The IV estimates assume linearity in the associations making up both the numerator and the denominator. This assumption was tested in the denominator by plotting mean father’s BP in centiles of son’s BP. For the numerator, sex-specific Cox models were estimated as described above, except that son’s BP was represented by a cubic spline with knots at the 5^th^, 35^th^, 65^th^ and 95^th^ percentiles^66^ for comparison with previously published results for own BP^10^.

Two-sample IV optimises power, but we may also wish to test whether IV estimates differ from analogous estimates made by a conventional multivariable analysis of parents’ own BP. For this purpose, one-sample IV estimates (where the numerator and denominator are estimated from the same sample) were made using the subsample of data in which father’s BP was known (Supplementary Fig. S1) and compared with conventional multivariable HR for a father’s mortality against his own BP. The one-sample IV estimates were made in exactly the way described above for the father-specific two-sample IV estimates. The conventional multivariable estimates were made with the same time axis, truncation, censoring and adjustment and were compared to the one-sample IV estimates using Durbin-Wu-Hausman tests^68^.

Statistical analyses were performed using Stata 14.1 on the University of Bristol supercomputer Blue Crystal and Stata 15.1 on a desktop machine. The study was performed in accordance with relevant guidelines and regulations and was approved by the Research Ethics Committee of the University of Bristol Faculty of Health Sciences (55841). Swedish national law and European guidelines do not require informed consent for research based on non-identifiable register-based data.

## Supplementary information

Supplementary material

## Data Availability

Swedish privacy laws prohibit us from making individual-level data publicly available. Researchers who are interested in replicating our work using individual-level data should apply to the appropriate Swedish authorities e.g. Statistics Sweden. For more information, see https://www.scb.se/en/services/guidance-for-researchers-and-universities/.
