# Author Correction: Associations of mortality with own blood pressure using son’s blood pressure as an instrumental variable

**DOI:** 10.1038/s41598-021-84494-1

**Published:** 2021-03-03

**Authors:** David Carslake, Abigail Fraser, Margaret T. May, Tom Palmer, Karri Silventoinen, Per Tynelius, Debbie A. Lawlor, George Davey Smith

**Affiliations:** 1 MRC Integrative Epidemiology Unit at the University of Bristol, Bristol, UK; 2 Bristol Medical School, Population Health Sciences, Bristol, UK; 3 Department of Mathematics and Statistics, University of Lancaster, Lancaster, UK; 4 Population Research Unit, Department of Social Research, University of Helsinki, Helsinki, Finland; 5 Department of Public Health Sciences, Karolinska Institute, Stockholm, Sweden

Correction to: *Scientific Reports* 10.1038/s41598-019-45391-w, published online 20 June 2019

This Article contains errors in the confidence intervals and P-values associated with some hazard ratios. A coding error meant that the standard errors of estimated log-hazard ratios were slightly underestimated whenever the estimate itself was negative (i.e. the hazard ratio was less than one). The error arose from the use of Stata local macros to represent negative values. These remain negative when squared; a problem easily remedied by bracketing the local macro before squaring.

As a result of the error, the precision of hazard ratios less than one is overestimated in Figures 1 and 2 and in Tables 2 and 3. The correct Figures 1 and 2 and Tables 2 and 3 appear below (note that changes to Figures 1 and 2 may be below the plotted resolution).

Additionally, Supplementary Tables S8–S17 are incorrect as a result of the same error. The corrected Supplementary Tables S8–S17 are linked to this correction notice.

Finally, F statistics and R^2^ in adjusted models in Table 1 are from the whole model, when they should have been partial statistics for the instrument. The correct Table 1 and its legend appear below.

**Figure 1:**
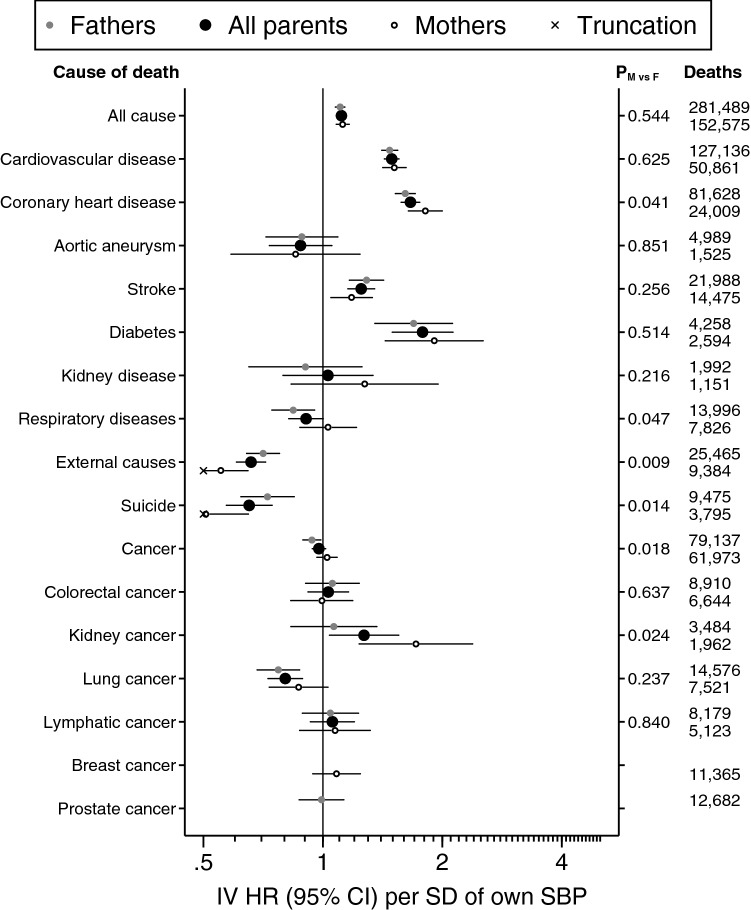
A correct version of the original Figure 1.

**Figure 2:**
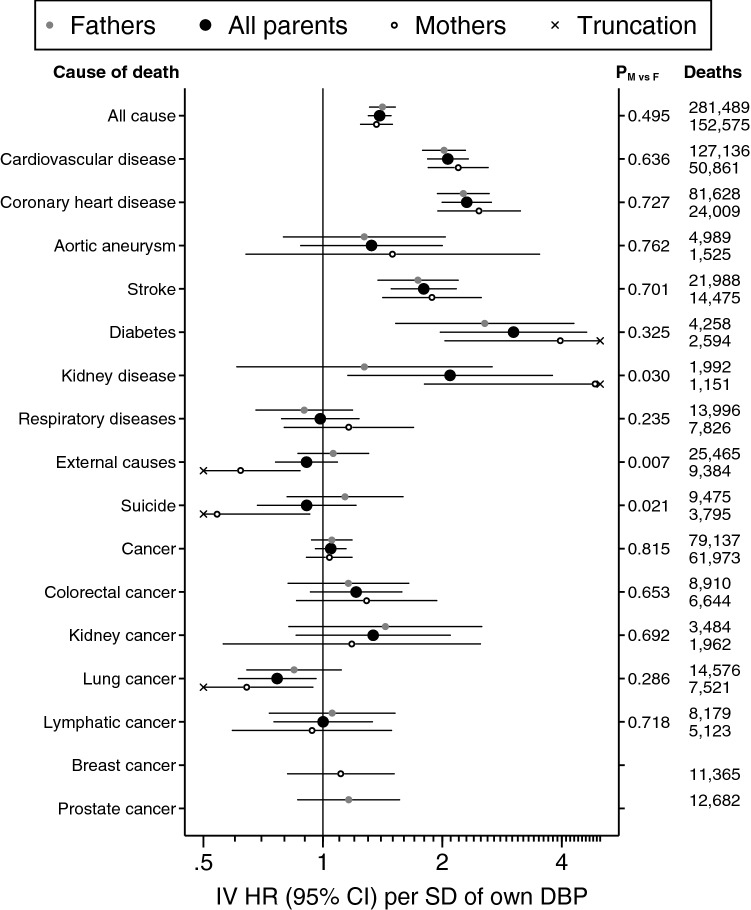
A correct version of the original Figure 2.

**Table 1:** Mean differences in father’s blood pressure per standard deviation (SD) of son’s blood pressure.

Blood pressure	Regression of father's blood pressure (SD) against son's blood pressure (SD)			
	Adjustment	Mean difference (95% CI)	F-statistic	R^2^
SBP	None	0.130 (0.122, 0.137)	1121.8	0.0166
SBP	Father's SEP	0.131 (0.123, 0.138)	1139.0	0.0168
SBP	Father's SEP, son's BMI	0.128 (0.120, 0.135)	1053.6	0.0156
DBP	None	0.060 (0.053, 0.067)	278.8	0.0042
DBP	Father's SEP	0.060 (0.053, 0.067)	274.8	0.0041
DBP	Father's SEP, son's BMI	0.059 (0.052, 0.066)	266.1	0.0040

Systolic blood pressure (SBP) and diastolic blood pressure (DBP) were each pre-adjusted for regional patterns, secular trends and age at examination. Blood pressure in fathers and sons was analysed in SD units (10.80 mmHg SBP and 9.22 mmHg DBP). Mean differences were obtained from linear regression and provide the denominators for the ratio method instrumental variable estimates. N = 66,567. Partial F-statistics and R^2^ are provided as measures of instrument strength.

**Table 2:** Adjusted hazard ratios (HR) for paternal mortality (i) per standard deviation (SD) of own systolic blood pressure (SBP) and (ii) per SD of own SBP, using son’s SBP as an instrumental variable (IV) within the subset having data on own SBP.

Cause of death	Deaths	HR (95% CI) per SD of own SBP	IV HR (95% CI) per SD of own SBP	P_own vs IV_
All cause	2332	1.03 (0.99, 1.07)	1.01 (0.74, 1.37)	0.873
Cardiovascular disease	423	1.21 (1.11, 1.33)	1.34 (0.65, 2.77)	0.779
Coronary heart disease	235	1.23 (1.09, 1.39)	1.91 (0.72, 5.04)	0.373
Stroke	86	1.21 (0.99, 1.48)	1.92 (0.39, 9.56)	0.568
External causes	1065	0.97 (0.92, 1.03)	0.94 (0.60, 1.48)	0.884
Suicide	466	0.95 (0.87, 1.04)	0.87 (0.44, 1.72)	0.780
Cancer	428	1.04 (0.95, 1.15)	1.00 (0.48, 2.05)	0.898
Brain cancer	61	1.15 (0.91, 1.47)	0.31 (0.05, 2.10)	0.175
Lung cancer	59	0.85 (0.66, 1.10)	1.24 (0.18, 8.66)	0.698
Lymphatic cancer	64	1.02 (0.80, 1.30)	0.35 (0.05, 2.24)	0.256

SBP was pre-adjusted for regional patterns, secular trends and age at examination and its SD was 10.80 mmHg. Cox proportional hazards models with age as the time axis were adjusted for educational and occupational socioeconomic position. One-sample IV estimates were made using the ratio method. P_own vs IV_ was derived from Durbin-Wu-Hausman tests comparing the two HR. N = 66,567 fathers at risk of mortality. Rarer causes of death (< 50 deaths in the data subset) are omitted.

**Table 3:** Adjusted hazard ratios (HR) for paternal mortality (i) per standard deviation (SD) of own diastolic blood pressure (DBP) and (ii) per SD of own DBP, using son’s DBP as an instrumental variable (IV) within the subset having data on own DBP.

Cause of death	Deaths	HR (95% CI) per SD of own DBP	IV HR (95% CI) per SD of own DBP	P_own vs IV_
All cause	2,332	1.01 (0.97, 1.06)	0.69 (0.35, 1.36)	0.266
Cardiovascular disease	423	1.11 (1.00, 1.23)	1.23 (0.25, 5.95)	0.901
Coronary heart disease	235	1.13 (0.98, 1.29)	2.70 (0.32, 22.66)	0.419
Stroke	86	1.14 (0.91, 1.43)	3.62 (0.11, 122.51)	0.520
External causes	1,065	0.97 (0.91, 1.04)	0.69 (0.25, 1.87)	0.497
Suicide	466	0.98 (0.89, 1.08)	0.90 (0.20, 4.05)	0.910
Cancer	428	1.03 (0.93, 1.14)	0.59 (0.12, 2.83)	0.487
Brain cancer	61	0.97 (0.74, 1.27)	0.29 (0.00, 18.59)	0.570
Lung cancer	59	0.84 (0.64, 1.11)	1.11 (0.02, 76.55)	0.900
Lymphatic cancer	64	0.94 (0.73, 1.22)	0.06 (0.00, 3.30)	0.174

DBP was pre-adjusted for regional patterns, secular trends and age at examination and its SD was 9.22 mmHg. Cox proportional hazards models with age as the time axis were adjusted for educational and occupational socioeconomic position. One-sample IV estimates were made using the ratio method. P_own vs IV_ was derived from Durbin-Wu-Hausman tests comparing the two HR. N = 66,567 fathers at risk of mortality. Rarer causes of death (< 50 deaths in the data subset) are omitted.

## Supplementary Information

Supplementary Information 1.

