# Supplementary material for: Associations of mortality with own blood pressure using son’s blood pressure as an instrumental variable
Source: Sci Rep. 2019 Jun 20;9:8986. doi: 10.1038/s41598-019-45391-w (PMC6586810; doi:10.1038/s41598-019-45391-w)

Supplementary material

Associations of mortality with own blood pressure using son's blood pressure as an instrumental variable

David Carslake[1,2]* Abigail Fraser[1,2], Margaret T May[2], Tom Palmer[3], Karri Silventoinen[4], Per Tynelius[5], Debbie A Lawlor[1,2], George Davey Smith[1,2]

[1]MRC Integrative Epidemiology Unit at the University of Bristol, Bristol, UK, [2]Population Health Sciences, Bristol Medical School, Bristol, UK, [3]Department of Mathematics and Statistics, University of Lancaster, Lancaster, UK 4 Population Research Unit, Department of Social Research, University of Helsinki, Helsinki, Finland, [5]Department of Public Health Sciences, Karolinska Institute, Stockholm, Sweden.
*Correspondence and requests for materials should be addressed to D. C. (email: David.Carslake@bristol.ac.uk)

Supplementary Table S1. International classification of diseases (ICD) codes comprising each cause of death.
Supplementary Table S2. Characteristics of sons and parents in the main dataset according to quintiles of son's systolic blood pressure (SBP), adjusted for regional patterns, secular trends and age at examination.
Supplementary Table S3. Characteristics of sons and parents in the main dataset according to quintiles of son's diastolic blood pressure (DBP), adjusted for regional patterns, secular trends and age at examination.
Supplementary Table S4. Characteristics of sons and parents in the subset with data on father's systolic blood pressure (SBP), according to quintiles of son's SBP, adjusted for regional patterns, secular trends and age at examination.
Supplementary Table S5. Characteristics of sons and parents in the subset with data on father's diastolic blood pressure (DBP), according to quintiles of son's DBP, adjusted for regional patterns, secular trends and age at examination.
Supplementary Table S6. Characteristics of sons and parents in the subset with data on father's systolic blood pressure (SBP), according to quintiles of father's own SBP, adjusted for regional patterns, secular trends and age at examination.
Supplementary Table S7. Characteristics of sons and parents in the subset with data on father's diastolic blood pressure (DBP), according to quintiles of father's own DBP, adjusted for regional patterns, secular trends and age at examination.
Supplementary Table S8. Adjusted hazard ratios (HR) for parental mortality (i) per standard deviation (SD) of a son's systolic blood pressure (SBP) and (ii) per SD of own SBP, using son's SBP as an instrumental variable (IV).
Supplementary Table S9. Adjusted hazard ratios (HR) for parental mortality (i) per standard deviation (SD) of a son's diastolic blood pressure (DBP) and (ii) per SD of own DBP, using son's DBP as an instrumental variable (IV).
Supplementary Table S10. Unadjusted (cf. Supplementary Table S8) hazard ratios (HR) for parental mortality (i) per standard deviation (SD) of a son's systolic blood pressure (SBP) and (ii) per SD of own SBP, using son's SBP as an instrumental variable (IV).
Supplementary Table S11. Unadjusted (cf. Supplementary Table S9) hazard ratios (HR) for parental mortality (i) per standard deviation (SD) of a son's diastolic blood pressure (DBP) and (ii) per SD of own DBP, using son's DBP as an instrumental variable (IV).

Supplementary Table S12. BMI-adjusted (cf. Supplementary Table S8) hazard ratios (HR) for parental mortality (i) per standard deviation (SD) of a son's systolic blood pressure (SBP) and (ii) per SD of own SBP, using son's SBP as an instrumental variable (IV).

Supplementary Table S13. BMI-adjusted (cf. Supplementary Table S9) hazard ratios (HR) for parental mortality (i) per standard deviation (SD) of a son's diastolic blood pressure (DBP) and (ii) per SD of own DBP, using son's DBP as an instrumental variable (IV).

Supplementary Table S14. Adjusted hazard ratios (HR) for parental cancer mortality (i) per standard deviation (SD) of a son's systolic blood pressure (SBP) and (ii) per SD of own SBP, using son's SBP as an instrumental variable (IV).

Supplementary Table S15. Adjusted hazard ratios (HR) for parental cancer mortality (i) per standard deviation (SD) of a son's diastolic blood pressure (DBP) and (ii) per SD of own DBP, using son's DBP as an instrumental variable (IV).

Supplementary Table S16. Unadjusted (cf. Table 2) hazard ratios (HR) for paternal mortality (i) per standard deviation (SD) of own systolic blood pressure (SBP) and (ii) per SD of own SBP, using son's SBP as an instrumental variable (IV) within the subset having data on own SBP.

Supplementary Table S17. Unadjusted (cf. Table 3) hazard ratios (HR) for paternal mortality (i) per standard deviation (SD) of own diastolic blood pressure (DBP) and (ii) per SD of own DBP, using son's DBP as an instrumental variable (IV) within the subset having data on own DBP.

Supplementary Table S18. Proportional hazards tests and adjusted age-specific hazard ratios (HR) for parental mortality per standard deviation (SD) of a son's systolic blood pressure (SBP).

Supplementary Table S19. Proportional hazards tests and adjusted age-specific hazard ratios (HR) for parental mortality per standard deviation (SD) of a son's diastolic blood pressure (DBP).

Supplementary Figure S1. Flow of participants through the study.

Supplementary Figure S2. Association of systolic (SBP) and diastolic (DBP) blood pressure between fathers and sons.

Supplementary Figure S3. Plots of hazard ratio (relative to the minimum hazard) for parental mortality against a son's systolic (SBP) or diastolic (DBP) blood pressure.

Supplementary Table S1. International classification of diseases (ICD) codes comprising each cause of death. ICD codes were recorded in 3-5 characters. Ranges are alphabetical, not numerical. For example, 12288<12289<1229<123<1230<12300<12301 and the range “123-12399” includes any 3-5 digit codes starting with “123”.

Diagnosis	ICD 7	ICD 8	ICD 9	ICD 10
Cardiovascular disease	330-33499<br>400-41699<br>420-42299<br>430-44799<br>450-46899<br>782-78299	390-40499<br>410-41499<br>420-42909<br>4299-43899<br>440-45899<br>782-78299	390-40599<br>410-43899<br>440-45999	G45-G4599<br>I00-I1599<br>I20-I5299<br>I60-I9999
Coronary heart disease	420-42099<br>422-42299<br>450-45099	410-41499<br>4299-42999	410-41499<br>4292-42929	I20-I2599<br>I516-I5169
Aortic aneurysm	451-45199	441-44199	441-44199	I71-I7199
Stroke	306-30699<br>330-33499<br>352-35299	2930-29319<br>344-34499<br>430-43899	2904-29049<br>342-34299<br>344-34499<br>430-43899	F01-F0199<br>G45-G4599<br>G81-G8199<br>G83-G8399<br>I60-I6999
Diabetes	260-26099	250-25099	250-25099	E10-E1499
Kidney disease	590-60499	580-59499	580-59499	N00-N2999
Respiratory diseases	240-24199<br>470-52799	460-51999	460-51999	J00-J9999
External causes	E800-E9999	E800-E9999	E800-E9999	V01-Y9899
Suicide	E970-E9799	E950-E9599	E950-E9599	X60-X8499
Cancer	140-20799	140-20799	140-20939	C00-C9799
Bladder cancer	1810-18109	188-18899	188-18899	C67-C6799
Brain cancer	193-19399	191-19299	191-19299	C71-C7299
Breast cancer	170-17099	174-17499	174-17599	C50-C5099
Colorectal cancer	153-15499	153-15499	153-15499	C18-C2199
Gallbladder cancer	1551-15519	156-15699	156-15699	C23-C2499
Kidney cancer	180-18099	1890-18929	1890-18929	C64-C6699
Liver cancer	155-15509<br>1552-15599	155-15599	155-15599	C22-C2299
Lung cancer	162-16219<br>1628-16399	162-16299	162-16299	C33-C3499
Lymphatic cancer	200-20799	200-20799	200-20899	C81-C9699
Malignant melanoma	190-19099	172-17299	172-17299	C43-C4399
Oesophageal cancer	150-15099	150-15099	150-15099	C15-C1599
Ovarian cancer	175-17599	183-18399	183-18399	C56-C5699<br>C570-C5749
Prostate cancer	177-17799	185-18599	185-18599	C61-C6199
Pancreatic cancer	157-15799	157-15799	157-15799	C25-C2599
Stomach cancer	151-15199	151-15199	151-15199	C16-C1699
Testicular cancer	178-17899	186-18699	186-18699	C62-C6299
Thyroid cancer	194-19499	193-19399	193-19399	C73-C7399
Uterine cancer	171-17499	180-18299	179-18289	C53-C5599
Cervical cancer	171-17199	180-18099	180-18099	C53-C5399
Endometrial cancer	172-17299	1820-18209	1820-18209	C54-C5499

Supplementary Table S2. Characteristics of sons and parents in the main dataset according to quintiles of son's systolic blood pressure (SBP), adjusted for regional patterns, secular trends and age at examination. [a]Measured at pre-conscription medical examination. Smoking was only recorded at examinations in 1969-1970. [b]Continuous variables are summarised as means in each quintile and mean differences per standard deviation (10.80 mmHg) of son's adjusted SBP. [c]Binary variables are summarised as percentages in each quintile and odds ratios per standard deviation of son's adjusted SBP.

Person and variable	Quintile of son's SBP 1st	2nd	3rd	4th	5th	Mean difference or odds ratio (95% CI)	N
Variables measured in the son:							
Unadjusted BMI[a,b] (kg m^{-2})	21.1	21.5	21.8	22.0	22.5	0.49 (0.48, 0.49)	1,023,232
Height[a,b] (cm)	178.6	179.1	179.3	179.6	179.9	0.48 (0.47, 0.49)	1,023,440
Systolic BP[a,b] (mm Hg)	113.8	122.1	128.5	134.3	143.7	10.80 (10.80, 10.80)	1,025,224
Diastolic BP[a,b] (mm Hg)	64.6	66.7	68.0	68.7	70.3	1.99 (1.97, 2.01)	1,025,224
Date of birth[b]	1965.6	1965.5	1966.0	1965.7	1966.1	0.00 (-0.02, 0.02)	1,025,224
Smokers[a,c] (%)	63%	61%	61%	58%	54%	0.87 (0.85, 0.89)	29,495
Variables measured in the father:							
Unadjusted BMI[a,b] (kg m^{-2})	21.1	21.2	21.2	21.3	21.4	0.09 (0.07, 0.11)	71,498
Height[a,b] (cm)	177.8	178.2	178.4	178.4	178.6	0.28 (0.24, 0.33)	71,516
Systolic BP[a,b] (mm Hg)	124.8	126.0	126.8	127.4	128.5	1.33 (1.25, 1.41)	71,501
Diastolic BP[a,b] (mm Hg)	70.8	71.2	71.5	71.5	72.3	0.51 (0.44, 0.57)	71,493
Date of birth[b]	1935.4	1935.1	1935.7	1935.2	1935.3	-0.19 (-0.21, -0.16)	1,014,139
Smokers[a,c] (%)	66%	63%	64%	65%	61%	0.95 (0.92, 0.98)	16,739
Age at son's birth (years)[b]	30.3	30.4	30.4	30.5	30.8	0.19 (0.17, 0.20)	1,014,139
Educated > 10 years[c] (%)	54%	54%	54%	53%	53%	0.97 (0.97, 0.97)	962,585
In non-manual work[c] (%)	49%	48%	49%	48%	48%	0.98 (0.97, 0.98)	849,027
Variables measured in the mother:							
Date of birth[b]	1938.5	1938.2	1938.7	1938.3	1938.4	-0.18 (-0.20, -0.16)	1,024,267
Age at son's birth (years)[b]	27.2	27.3	27.3	27.4	27.6	0.18 (0.17, 0.19)	1,024,267
Educated > 10 years[c] (%)	54%	53%	54%	53%	53%	0.96 (0.96, 0.97)	1,004,985
In non-manual work[c] (%)	48%	47%	47%	47%	47%	0.98 (0.97, 0.98)	743,386

Supplementary Table S3. Characteristics of sons and parents in the main dataset according to quintiles of son's diastolic blood pressure (DBP), adjusted for regional patterns, secular trends and age at examination. [a]Measured at pre-conscription medical examination. Smoking was only recorded at examinations in 1969-1970. [b]Continuous variables are summarised as means in each quintile and mean differences per standard deviation (9.22 mmHg) of son's adjusted DBP. [c]Binary variables are summarised as percentages in each quintile and odds ratios per standard deviation of son's adjusted DBP.

	Quintile of son's DBP					Mean difference or	
Person and variable	1st	2nd	3rd	4th	5th	odds ratio (95% CI)	N
Variables measured in the son:							
Unadjusted BMI[a,b] (kg m^{-2})	21.5	21.6	21.6	21.8	22.2	0.25 (0.24, 0.25)	1,023,232
Height[a,b] (cm)	179.1	179.2	179.3	179.4	179.4	0.11 (0.10, 0.12)	1,023,440
Systolic BP[a,b] (mm Hg)	126.2	126.6	128.0	129.0	132.6	2.33 (2.31, 2.35)	1,025,224
Diastolic BP[a,b] (mm Hg)	54.4	62.3	67.9	72.9	80.6	9.22 (9.22, 9.23)	1,025,224
Date of birth[b]	1966.1	1965.8	1965.6	1965.4	1966.1	0.00 (-0.02, 0.02)	1,025,224
Smokers[a,c] (%)	63%	61%	60%	58%	55%	0.88 (0.86, 0.90)	29,495
Variables measured in the father:							
Unadjusted BMI[a,b] (kg m^{-2})	21.2	21.3	21.2	21.2	21.3	0.01 (-0.01, 0.02)	71,498
Height[a,b] (cm)	178.1	178.1	178.5	178.2	178.4	0.08 (0.04, 0.13)	71,516
Systolic BP[a,b] (mm Hg)	126.0	126.5	126.5	126.8	127.7	0.51 (0.43, 0.59)	71,501
Diastolic BP[a,b] (mm Hg)	70.9	71.2	71.3	71.6	72.3	0.43 (0.36, 0.49)	71,493
Date of birth[b]	1935.8	1935.4	1935.1	1934.9	1935.6	-0.07 (-0.10, -0.05)	1,014,139
Smokers[a,c] (%)	65%	64%	62%	65%	63%	0.99 (0.96, 1.02)	16,739
Age at son's birth (years)[b]	30.4	30.4	30.5	30.6	30.6	0.07 (0.06, 0.09)	1,014,139
Educated > 10 years[c] (%)	55%	54%	54%	52%	53%	0.97 (0.96, 0.97)	962,585
In non-manual work[c] (%)	49%	49%	49%	48%	47%	0.97 (0.97, 0.98)	849,027
Variables measured in the mother:							
Date of birth[b]	1938.9	1938.5	1938.2	1938.0	1938.7	-0.05 (-0.08, -0.03)	1,024,267
Age at son's birth (years)[b]	27.3	27.3	27.4	27.4	27.4	0.05 (0.04, 0.07)	1,024,267
Educated > 10 years[c] (%)	55%	54%	53%	52%	53%	0.97 (0.96, 0.97)	1,004,985
In non-manual work[c] (%)	48%	48%	47%	46%	47%	0.97 (0.97, 0.98)	743,386

Supplementary Table S4. Characteristics of sons and parents in the subset with data on father's systolic blood pressure (SBP), according to quintiles of son's SBP, adjusted for regional patterns, secular trends and age at examination. [a]Measured at pre-conscription medical examination. Smoking was only recorded at examinations in 1969-1970. [b]Continuous variables are summarised as means in each quintile and mean differences per standard deviation (10.80 mmHg) of son's adjusted SBP. [c]Binary variables are summarised as percentages in each quintile and odds ratios per standard deviation of son's adjusted SBP.

Person and variable	Quintile of son's SBP 1st	2nd	3rd	4th	5th	Mean difference or odds ratio (95% CI)	N
Variables measured in the son:							
Unadjusted BMI[a,b] (kg m^{-2})	21.7	22.2	22.4	22.7	23.1	0.51 (0.49, 0.53)	66,329
Height[a,b] (cm)	178.6	179.2	179.5	179.8	180.1	0.52 (0.47, 0.57)	66,335
Systolic BP[a,b] (mm Hg)	113.3	122.0	128.8	134.7	142.7	10.74 (10.73, 10.75)	66,567
Diastolic BP[a,b] (mm Hg)	62.6	65.5	67.5	68.7	71.0	2.99 (2.92, 3.05)	66,567
Date of birth[b]	1977.6	1977.8	1977.8	1977.6	1977.9	0.06 (0.04, 0.08)	66,567
Variables measured in the father:							
Unadjusted BMI[a,b] (kg m^{-2})	21.1	21.2	21.2	21.3	21.4	0.09 (0.07, 0.11)	66,539
Height[a,b] (cm)	177.8	178.1	178.4	178.4	178.6	0.29 (0.24, 0.34)	66,554
Systolic BP[a,b] (mm Hg)	124.9	126.0	126.8	127.5	128.6	1.32 (1.24, 1.40)	66,567
Diastolic BP[a,b] (mm Hg)	70.8	71.1	71.4	71.5	72.3	0.50 (0.43, 0.56)	66,567
Date of birth[b]	1953.6	1953.7	1953.6	1953.6	1953.6	-0.03 (-0.04, -0.01)	66,567
Smokers[a,c] (%)	67%	64%	65%	65%	63%	0.96 (0.92, 0.99)	13,450
Age at son's birth (years)[b]	23.9	24.1	24.1	24.0	24.2	0.09 (0.07, 0.11)	66,567
Educated > 10 years[c] (%)	64%	66%	66%	65%	68%	1.04 (1.02, 1.06)	65,048
In non-manual work[c] (%)	41%	43%	43%	43%	44%	1.03 (1.02, 1.05)	57,105
Variables measured in the mother:							
Date of birth[b]	1954.8	1954.9	1954.8	1954.7	1954.7	-0.04 (-0.06, -0.01)	66,567
Age at son's birth (years)[b]	22.8	23.0	23.0	22.8	23.1	0.09 (0.07, 0.12)	66,567
Educated > 10 years[c] (%)	70%	72%	72%	71%	72%	1.03 (1.02, 1.05)	65,262
In non-manual work[c] (%)	42%	44%	44%	44%	45%	1.03 (1.02, 1.05)	55,253

Supplementary Table S5. Characteristics of sons and parents in the subset with data on father's diastolic blood pressure (DBP), according to quintiles of son's DBP, adjusted for regional patterns, secular trends and age at examination. [a]Measured at pre-conscription medical examination. Smoking was only recorded at examinations in 1969-1970. [b]Continuous variables are summarised as means in each quintile and mean differences per standard deviation (9.22 mmHg) of son's adjusted DBP. [c]Binary variables are summarised as percentages in each quintile and odds ratios per standard deviation of son's adjusted DBP.

	Quintile of son's DBP					Mean difference or	
Person and variable	1st	2nd	3rd	4th	5th	odds ratio (95% CI)	N
Variables measured in the son:							
Unadjusted BMI[a,b] (kg m^{-2})	22.3	22.3	22.3	22.4	22.7	0.14 (0.12, 0.17)	66,329
Height[a,b] (cm)	179.3	179.3	179.6	179.5	179.7	0.16 (0.11, 0.20)	66,335
Systolic BP[a,b] (mm Hg)	124.0	125.6	128.1	129.7	133.9	3.48 (3.40, 3.56)	66,567
Diastolic BP[a,b] (mm Hg)	53.8	61.3	67.8	71.9	79.8	9.14 (9.13, 9.16)	66,567
Date of birth[b]	1977.5	1977.7	1978.0	1977.7	1977.7	0.01 (-0.01, 0.03)	66,567
Variables measured in the father:							
Unadjusted BMI[a,b] (kg m^{-2})	21.2	21.3	21.2	21.2	21.3	0.01 (-0.01, 0.02)	66,539
Height[a,b] (cm)	178.1	178.1	178.5	178.2	178.4	0.07 (0.03, 0.12)	66,554
Systolic BP[a,b] (mm Hg)	126.1	126.5	126.6	126.8	127.8	0.52 (0.44, 0.60)	66,567
Diastolic BP[a,b] (mm Hg)	70.9	71.2	71.3	71.6	72.2	0.43 (0.37, 0.50)	66,567
Date of birth[b]	1953.6	1953.6	1953.7	1953.6	1953.6	-0.01 (-0.03, 0.00)	66,567
Smokers[a,c] (%)	66%	65%	63%	65%	65%	0.99 (0.95, 1.02)	13,450
Age at son's birth (years)[b]	23.9	24.1	24.3	24.1	24.1	0.03 (0.01, 0.05)	66,567
Educated > 10 years[c] (%)	68%	66%	66%	65%	64%	0.95 (0.94, 0.97)	65,048
In non-manual work[c] (%)	43%	42%	44%	43%	43%	1.00 (0.99, 1.02)	57,105
Variables measured in the mother:							
Date of birth[b]	1954.7	1954.8	1954.9	1954.8	1954.7	-0.02 (-0.04, 0.01)	66,567
Age at son's birth (years)[b]	22.8	22.9	23.1	22.9	23.0	0.03 (0.00, 0.06)	66,567
Educated > 10 years[c] (%)	72%	72%	73%	71%	70%	0.97 (0.96, 0.99)	65,262
In non-manual work[c] (%)	44%	43%	45%	43%	43%	0.99 (0.97, 1.01)	55,253

Supplementary Table S6. Characteristics of sons and parents in the subset with data on father's systolic blood pressure (SBP), according to quintiles of father's own SBP, adjusted for regional patterns, secular trends and age at examination. [a]Measured at pre-conscription medical examination. Smoking was only recorded at examinations in 1969-1970. [b]Continuous variables are summarised as means in each quintile and mean differences per 10.80 mmHg of father's own adjusted SBP. [c]Binary variables are summarised as percentages in each quintile and odds ratios per 10.80 mmHg of father's own adjusted SBP.

	Quintile of father's SBP					Mean difference or	
Person and variable	1st	2nd	3rd	4th	5th	odds ratio (95% CI)	N
Variables measured in the son:							
Unadjusted BMI[a,b] (kg m^{-2})	22.2	22.3	22.4	22.5	22.6	0.14 (0.12, 0.16)	66,329
Height[a,b] (cm)	179.2	179.4	179.3	179.6	179.8	0.21 (0.16, 0.26)	66,335
Systolic BP[a,b] (mm Hg)	126.5	127.7	128.1	129.0	130.4	1.36 (1.28, 1.45)	66,567
Diastolic BP[a,b] (mm Hg)	66.6	66.8	67.0	67.2	67.9	0.45 (0.38, 0.52)	66,567
Date of birth[b]	1977.6	1977.7	1977.8	1977.7	1977.8	0.06 (0.04, 0.08)	66,567
Variables measured in the father:							
Unadjusted BMI[a,b] (kg m^{-2})	20.7	21.0	21.2	21.5	21.9	0.43 (0.41, 0.44)	66,539
Height[a,b] (cm)	177.6	178.1	178.3	178.4	178.8	0.41 (0.36, 0.46)	66,554
Systolic BP[a,b] (mm Hg)	112.1	120.4	126.4	132.2	142.7	10.80 (10.79, 10.81)	66,567
Diastolic BP[a,b] (mm Hg)	67.6	69.9	71.6	73.0	75.0	2.58 (2.52, 2.64)	66,567
Date of birth[b]	1953.6	1953.6	1953.8	1953.5	1953.6	0.00 (-0.02, 0.02)	66,567
Smokers[a,c] (%)	68%	67%	65%	64%	59%	0.87 (0.84, 0.90)	13,450
Age at son's birth (years)[b]	24.1	24.1	24.0	24.1	24.2	0.06 (0.04, 0.08)	66,567
Educated > 10 years[c] (%)	67%	66%	66%	65%	64%	0.95 (0.93, 0.96)	65,048
In non-manual work[c] (%)	45%	43%	42%	42%	41%	0.96 (0.94, 0.97)	57,105
Variables measured in the mother:							
Date of birth[b]	1954.7	1954.7	1954.9	1954.7	1954.8	-0.01 (-0.03, 0.02)	66,567
Age at son's birth (years)[b]	22.9	23.0	22.8	23.0	23.0	0.06 (0.04, 0.09)	66,567
Educated > 10 years[c] (%)	72%	72%	71%	72%	71%	0.99 (0.97, 1.00)	65,262
In non-manual work[c] (%)	45%	44%	43%	43%	42%	0.97 (0.95, 0.98)	55,253

Supplementary Table S7. Characteristics of sons and parents in the subset with data on father's diastolic blood pressure (DBP), according to quintiles of father's own DBP, adjusted for regional patterns, secular trends and age at examination. [a]Measured at pre-conscription medical examination. Smoking was only recorded at examinations in 1969-1970. [b]Continuous variables are summarised as means in each quintile and mean differences per 9.22 mmHg of father's own adjusted DBP. [c]Binary variables are summarised as percentages in each quintile and odds ratios per 9.22 mmHg of father's own adjusted DBP.

	Quintile of father's DBP					Mean difference or	
Person and variable	1st	2nd	3rd	4th	5th	odds ratio (95% CI)	N
Variables measured in the son:							
Unadjusted BMI[a,b] (kg m^{-2})	22.3	22.4	22.3	22.5	22.6	0.11 (0.08, 0.14)	66,329
Height[a,b] (cm)	179.3	179.4	179.5	179.5	179.6	0.10 (0.05, 0.15)	66,335
Systolic BP[a,b] (mm Hg)	127.7	128.1	128.2	128.5	129.2	0.60 (0.51, 0.68)	66,567
Diastolic BP[a,b] (mm Hg)	66.4	66.8	67.0	67.2	68.0	0.64 (0.56, 0.71)	66,567
Date of birth[b]	1977.7	1977.7	1977.8	1977.7	1977.7	0.04 (0.02, 0.06)	66,567
Variables measured in the father:							
Unadjusted BMI[a,b] (kg m^{-2})	20.9	21.0	21.2	21.4	21.8	0.36 (0.34, 0.38)	66,539
Height[a,b] (cm)	177.9	178.1	178.3	178.4	178.5	0.22 (0.17, 0.27)	66,554
Systolic BP[a,b] (mm Hg)	123.0	124.8	125.9	127.9	132.2	3.53 (3.44, 3.61)	66,567
Diastolic BP[a,b] (mm Hg)	59.1	67.3	71.0	76.5	83.3	9.22 (9.21, 9.23)	66,567
Date of birth[b]	1953.6	1953.6	1953.7	1953.6	1953.6	0.00 (-0.02, 0.02)	66,567
Smokers[a,c] (%)	69%	66%	64%	64%	61%	0.89 (0.86, 0.93)	13,450
Age at son's birth (years)[b]	24.0	24.1	24.1	24.1	24.1	0.04 (0.02, 0.06)	66,567
Educated > 10 years[c] (%)	66%	67%	66%	66%	64%	0.97 (0.95, 0.99)	65,048
In non-manual work[c] (%)	43%	43%	43%	43%	42%	0.99 (0.97, 1.01)	57,105
Variables measured in the mother:							
Date of birth[b]	1954.8	1954.8	1954.8	1954.7	1954.7	-0.02 (-0.05, 0.01)	66,567
Age at son's birth (years)[b]	22.9	22.9	22.9	23.0	23.0	0.06 (0.03, 0.09)	66,567
Educated > 10 years[c] (%)	71%	72%	72%	72%	70%	0.98 (0.97, 1.00)	65,262
In non-manual work[c] (%)	44%	44%	44%	44%	43%	0.99 (0.97, 1.00)	55,253

Supplementary Table S8. Adjusted hazard ratios (HR) for parental mortality (i) per standard deviation (SD) of a son's systolic blood pressure (SBP) and (ii) per SD of own SBP, using son's SBP as an instrumental variable (IV). SBP was pre-adjusted for regional patterns, secular trends and age at examination and its SD was 10.80 mmHg. Cox proportional hazards models with age as the time axis were adjusted for parental sex and for educational and occupational socioeconomic position. Robust standard errors were clustered by the son's identity. $P_{M\ vs\ F}$ was derived from a Z-test of an additional interaction term between parental sex and son's SBP. Two-sample IV estimates were made using the ratio method. Mothers and fathers were also modelled separately without the robust standard errors or the adjustment for parental sex. N = 1,002,031 mothers and 986,075 fathers at risk of mortality.

	Deaths		HR (95% CI) per SD of son's SBP			IV HR (95% CI) per SD of own SBP			
Cause of death	Fathers	Mothers	Fathers	Mothers	All parents	Fathers	Mothers	All parents	$P_{F\ vs\ M}$
All cause	281,489	152,575	1.01 (1.01, 1.02)	1.02 (1.01, 1.02)	1.01 (1.01, 1.02)	1.11 (1.07, 1.14)	1.12 (1.08, 1.17)	1.11 (1.09, 1.14)	0.544
Cardiovascular disease	127,136	50,861	1.05 (1.05, 1.06)	1.06 (1.05, 1.06)	1.05 (1.05, 1.06)	1.47 (1.41, 1.54)	1.51 (1.41, 1.62)	1.49 (1.43, 1.56)	0.625
Coronary heart disease	81,628	24,009	1.06 (1.06, 1.07)	1.08 (1.07, 1.09)	1.07 (1.06, 1.08)	1.61 (1.52, 1.71)	1.81 (1.64, 2.00)	1.66 (1.57, 1.75)	0.041
Aortic aneurysm	4,989	1,525	0.98 (0.96, 1.01)	0.98 (0.93, 1.03)	0.98 (0.96, 1.01)	0.89 (0.72, 1.09)	0.85 (0.59, 1.24)	0.88 (0.73, 1.05)	0.851
Stroke	21,988	14,475	1.03 (1.02, 1.05)	1.02 (1.01, 1.04)	1.03 (1.02, 1.04)	1.29 (1.17, 1.42)	1.18 (1.05, 1.33)	1.25 (1.16, 1.35)	0.256
Diabetes	4,258	2,594	1.07 (1.04, 1.10)	1.09 (1.05, 1.13)	1.08 (1.05, 1.10)	1.69 (1.35, 2.12)	1.91 (1.44, 2.54)	1.78 (1.49, 2.13)	0.514
Kidney disease	1,992	1,151	0.99 (0.95, 1.03)	1.03 (0.98, 1.09)	1.00 (0.97, 1.04)	0.90 (0.65, 1.26)	1.27 (0.83, 1.95)	1.03 (0.79, 1.34)	0.216
Respiratory diseases	13,996	7,826	0.98 (0.96, 0.99)	1.00 (0.98, 1.03)	0.99 (0.97, 1.00)	0.84 (0.74, 0.95)	1.03 (0.87, 1.22)	0.91 (0.82, 1.00)	0.047
External causes	25,465	9,384	0.96 (0.94, 0.97)	0.93 (0.91, 0.94)	0.95 (0.94, 0.96)	0.71 (0.64, 0.77)	0.55 (0.48, 0.64)	0.66 (0.61, 0.71)	0.009
Suicide	9,475	3,795	0.96 (0.94, 0.98)	0.92 (0.89, 0.94)	0.95 (0.93, 0.96)	0.73 (0.62, 0.84)	0.51 (0.40, 0.64)	0.65 (0.57, 0.74)	0.014
Cancer	79,137	61,973	0.99 (0.98, 1.00)	1.00 (1.00, 1.01)	1.00 (0.99, 1.00)	0.94 (0.89, 0.99)	1.02 (0.97, 1.09)	0.98 (0.94, 1.02)	0.018
Colorectal cancer	8,910	6,644	1.01 (0.99, 1.03)	1.00 (0.98, 1.02)	1.00 (0.99, 1.02)	1.06 (0.90, 1.24)	0.99 (0.83, 1.19)	1.03 (0.92, 1.16)	0.637
Kidney cancer	3,484	1,962	1.01 (0.98, 1.04)	1.07 (1.03, 1.12)	1.03 (1.00, 1.06)	1.07 (0.83, 1.37)	1.71 (1.23, 2.39)	1.27 (1.04, 1.55)	0.024
Lung cancer	14,576	7,521	0.97 (0.95, 0.98)	0.98 (0.96, 1.00)	0.97 (0.96, 0.98)	0.77 (0.68, 0.87)	0.87 (0.73, 1.03)	0.80 (0.73, 0.89)	0.237
Lymphatic cancer	8,179	5,123	1.01 (0.98, 1.03)	1.01 (0.98, 1.04)	1.01 (0.99, 1.02)	1.04 (0.89, 1.23)	1.07 (0.87, 1.32)	1.06 (0.93, 1.20)	0.840
Breast cancer		11,365		1.01 (0.99, 1.03)			1.08 (0.94, 1.24)		
Prostate cancer	12,682		1.00 (0.98, 1.02)			0.99 (0.87, 1.13)			

Supplementary Table S9. Adjusted hazard ratios (HR) for parental mortality (i) per standard deviation (SD) of a son's diastolic blood pressure (DBP) and (ii) per SD of own DBP, using son's DBP as an instrumental variable (IV). DBP was pre-adjusted for regional patterns, secular trends and age at examination and its SD was 9.22 mmHg. Cox proportional hazards models with age as the time axis were adjusted for parental sex and for educational and occupational socioeconomic position. Robust standard errors were clustered by the son's identity. $P_{M\ vs\ F}$ was derived from a Z-test of an additional interaction term between parental sex and son's DBP. Two-sample IV estimates were made using the ratio method. Mothers and fathers were also modelled separately without the robust standard errors or the adjustment for parental sex. N = 1,002,031 mothers and 986,075 fathers at risk of mortality.

	Deaths		HR (95% CI) per SD of son's DBP			IV HR (95% CI) per SD of own DBP			
Cause of death	Fathers	Mothers	Fathers	Mothers	All parents	Fathers	Mothers	All parents	$P_{F\ vs\ M}$
All cause	281,489	152,575	1.02 (1.02, 1.02)	1.02 (1.01, 1.02)	1.02 (1.02, 1.02)	1.41 (1.31, 1.52)	1.37 (1.24, 1.50)	1.39 (1.30, 1.48)	0.495
Cardiovascular disease	127,136	50,861	1.04 (1.04, 1.05)	1.05 (1.04, 1.06)	1.04 (1.04, 1.05)	2.02 (1.78, 2.29)	2.19 (1.84, 2.61)	2.07 (1.84, 2.32)	0.636
Coronary heart disease	81,628	24,009	1.05 (1.04, 1.06)	1.06 (1.04, 1.07)	1.05 (1.04, 1.06)	2.26 (1.94, 2.63)	2.48 (1.95, 3.15)	2.30 (2.00, 2.66)	0.727
Aortic aneurysm	4,989	1,525	1.01 (0.99, 1.04)	1.02 (0.97, 1.08)	1.02 (0.99, 1.04)	1.27 (0.80, 2.03)	1.50 (0.64, 3.52)	1.33 (0.88, 2.00)	0.762
Stroke	21,988	14,475	1.03 (1.02, 1.05)	1.04 (1.02, 1.06)	1.04 (1.02, 1.05)	1.74 (1.38, 2.19)	1.88 (1.42, 2.51)	1.80 (1.49, 2.17)	0.701
Diabetes	4,258	2,594	1.06 (1.03, 1.09)	1.09 (1.04, 1.13)	1.07 (1.04, 1.09)	2.56 (1.52, 4.29)	3.97 (2.03, 7.76)	3.02 (1.98, 4.62)	0.325
Kidney disease	1,992	1,151	1.01 (0.97, 1.06)	1.10 (1.04, 1.17)	1.05 (1.01, 1.08)	1.27 (0.61, 2.67)	4.86 (1.80, 13.10)	2.09 (1.16, 3.79)	0.030
Respiratory diseases	13,996	7,826	0.99 (0.98, 1.01)	1.01 (0.99, 1.03)	1.00 (0.99, 1.01)	0.90 (0.68, 1.19)	1.16 (0.80, 1.69)	0.99 (0.79, 1.23)	0.235
External causes	25,465	9,384	1.00 (0.99, 1.02)	0.97 (0.95, 0.99)	0.99 (0.98, 1.01)	1.06 (0.87, 1.30)	0.62 (0.44, 0.87)	0.91 (0.76, 1.09)	0.007
Suicide	9,475	3,795	1.01 (0.99, 1.03)	0.96 (0.93, 1.00)	0.99 (0.98, 1.01)	1.14 (0.81, 1.59)	0.54 (0.32, 0.92)	0.91 (0.68, 1.21)	0.021
Cancer	79,137	61,973	1.00 (1.00, 1.01)	1.00 (0.99, 1.01)	1.00 (1.00, 1.01)	1.05 (0.94, 1.18)	1.04 (0.91, 1.18)	1.05 (0.96, 1.14)	0.815
Colorectal cancer	8,910	6,644	1.01 (0.99, 1.03)	1.02 (0.99, 1.04)	1.01 (1.00, 1.03)	1.16 (0.82, 1.65)	1.29 (0.86, 1.94)	1.21 (0.93, 1.58)	0.653
Kidney cancer	3,484	1,962	1.02 (0.99, 1.06)	1.01 (0.97, 1.06)	1.02 (0.99, 1.05)	1.44 (0.82, 2.52)	1.18 (0.56, 2.49)	1.34 (0.86, 2.09)	0.692
Lung cancer	14,576	7,521	0.99 (0.97, 1.01)	0.97 (0.95, 1.00)	0.98 (0.97, 1.00)	0.85 (0.64, 1.11)	0.64 (0.44, 0.94)	0.77 (0.61, 0.96)	0.286
Lymphatic cancer	8,179	5,123	1.00 (0.98, 1.03)	1.00 (0.97, 1.02)	1.00 (0.98, 1.02)	1.06 (0.73, 1.52)	0.94 (0.59, 1.49)	1.00 (0.75, 1.33)	0.718
Breast cancer		11,365		1.01 (0.99, 1.03)			1.11 (0.81, 1.51)		
Prostate cancer	12,682		1.01 (0.99, 1.03)			1.16 (0.86, 1.56)			

Supplementary Table S10. Unadjusted (cf. Supplementary Table S8) hazard ratios (HR) for parental mortality (i) per standard deviation (SD) of a son's systolic blood pressure (SBP) and (ii) per SD of own SBP, using son's SBP as an instrumental variable (IV). SBP was pre-adjusted for regional patterns, secular trends and age at examination and its SD was 10.80 mmHg. Cox proportional hazards models with age as the time axis were adjusted for parental sex only. Robust standard errors were clustered by the son's identity. $P_{M\ vs\ F}$ was derived from a Z-test of an additional interaction term between parental sex and son's SBP. Two-sample IV estimates were made using the ratio method. Mothers and fathers were also modelled separately without the robust standard errors or the adjustment for parental sex. N = 1,002,031 mothers and 986,075 fathers at risk of mortality.

	Deaths		HR (95% CI) per SD of son's SBP			IV HR (95% CI) per SD of own SBP			
Cause of death	Fathers	Mothers	Fathers	Mothers	All parents	Fathers	Mothers	All parents	$P_{F\ vs\ M}$
All cause	281,489	152,575	1.02 (1.02, 1.02)	1.03 (1.02, 1.03)	1.02 (1.02, 1.03)	1.17 (1.14, 1.20)	1.22 (1.17, 1.26)	1.19 (1.16, 1.22)	0.093
Cardiovascular disease	127,136	50,861	1.06 (1.06, 1.07)	1.07 (1.06, 1.08)	1.06 (1.06, 1.07)	1.58 (1.50, 1.66)	1.69 (1.58, 1.82)	1.62 (1.54, 1.69)	0.045
Coronary heart disease	81,628	24,009	1.07 (1.07, 1.08)	1.10 (1.08, 1.11)	1.08 (1.07, 1.09)	1.74 (1.64, 1.85)	2.05 (1.85, 2.27)	1.81 (1.71, 1.92)	<0.001
Aortic aneurysm	4,989	1,525	0.99 (0.96, 1.01)	0.98 (0.94, 1.03)	0.99 (0.96, 1.01)	0.90 (0.73, 1.11)	0.89 (0.61, 1.30)	0.90 (0.75, 1.08)	0.943
Stroke	21,988	14,475	1.04 (1.03, 1.05)	1.04 (1.02, 1.05)	1.04 (1.03, 1.05)	1.36 (1.23, 1.51)	1.31 (1.16, 1.47)	1.34 (1.24, 1.45)	0.562
Diabetes	4,258	2,594	1.08 (1.05, 1.11)	1.11 (1.07, 1.15)	1.09 (1.07, 1.12)	1.83 (1.46, 2.29)	2.25 (1.69, 3.00)	1.98 (1.66, 2.37)	0.237
Kidney disease	1,992	1,151	1.00 (0.95, 1.04)	1.05 (1.00, 1.12)	1.02 (0.98, 1.05)	0.97 (0.70, 1.35)	1.51 (0.98, 2.33)	1.14 (0.88, 1.49)	0.115
Respiratory diseases	13,996	7,826	0.99 (0.97, 1.00)	1.02 (0.99, 1.04)	1.00 (0.98, 1.01)	0.91 (0.80, 1.03)	1.13 (0.96, 1.34)	0.98 (0.89, 1.09)	0.036
External causes	25,465	9,384	0.96 (0.94, 0.97)	0.93 (0.91, 0.95)	0.95 (0.94, 0.96)	0.71 (0.64, 0.77)	0.58 (0.49, 0.67)	0.67 (0.62, 0.72)	0.035
Suicide	9,475	3,795	0.96 (0.94, 0.98)	0.92 (0.89, 0.95)	0.94 (0.93, 0.96)	0.71 (0.61, 0.83)	0.51 (0.40, 0.65)	0.65 (0.57, 0.74)	0.027
Cancer	79,137	61,973	1.00 (0.99, 1.00)	1.01 (1.00, 1.02)	1.00 (1.00, 1.01)	0.96 (0.91, 1.02)	1.08 (1.01, 1.14)	1.01 (0.97, 1.05)	0.011
Colorectal cancer	8,910	6,644	1.01 (0.99, 1.03)	1.01 (0.98, 1.03)	1.01 (0.99, 1.02)	1.08 (0.92, 1.26)	1.04 (0.87, 1.25)	1.06 (0.94, 1.20)	0.771
Kidney cancer	3,484	1,962	1.01 (0.98, 1.05)	1.08 (1.04, 1.13)	1.04 (1.01, 1.07)	1.10 (0.85, 1.41)	1.86 (1.33, 2.59)	1.33 (1.09, 1.63)	0.014
Lung cancer	14,576	7,521	0.97 (0.96, 0.99)	0.98 (0.96, 1.01)	0.98 (0.96, 0.99)	0.80 (0.71, 0.91)	0.88 (0.74, 1.05)	0.83 (0.75, 0.92)	0.410
Lymphatic cancer	8,179	5,123	1.01 (0.99, 1.03)	1.02 (0.99, 1.04)	1.01 (1.00, 1.03)	1.07 (0.91, 1.26)	1.14 (0.92, 1.40)	1.10 (0.96, 1.25)	0.659
Breast cancer		11,365		1.01 (1.00, 1.03)			1.12 (0.97, 1.28)		
Prostate cancer	12,682		1.00 (0.98, 1.02)			0.99 (0.87, 1.13)			

Supplementary Table S11. Unadjusted (cf. Supplementary Table S9) hazard ratios (HR) for parental mortality (i) per standard deviation (SD) of a son's diastolic blood pressure (DBP) and (ii) per SD of own DBP, using son's DBP as an instrumental variable (IV). DBP was pre-adjusted for regional patterns, secular trends and age at examination and its SD was 9.22 mmHg. Cox proportional hazards models with age as the time axis were adjusted for parental sex only. Robust standard errors were clustered by the son's identity. $P_{M\ vs\ F}$ was derived from a Z-test of an additional interaction term between parental sex and son's DBP. Two-sample IV estimates were made using the ratio method. Mothers and fathers were also modelled separately without the robust standard errors or the adjustment for parental sex. N = 1,002,031 mothers and 986,075 fathers at risk of mortality.

	Deaths		HR (95% CI) per SD of son's DBP			IV HR (95% CI) per SD of own DBP			
Cause of death	Fathers	Mothers	Fathers	Mothers	All parents	Fathers	Mothers	All parents	$P_{F\ vs\ M}$
All cause	281,489	152,575	1.02 (1.02, 1.03)	1.03 (1.02, 1.03)	1.02 (1.02, 1.03)	1.49 (1.38, 1.61)	1.53 (1.39, 1.69)	1.50 (1.40, 1.61)	0.617
Cardiovascular disease	127,136	50,861	1.05 (1.04, 1.05)	1.06 (1.05, 1.07)	1.05 (1.05, 1.06)	2.18 (1.91, 2.48)	2.58 (2.14, 3.09)	2.29 (2.02, 2.60)	0.101
Coronary heart disease	81,628	24,009	1.06 (1.05, 1.06)	1.07 (1.05, 1.08)	1.06 (1.05, 1.07)	2.47 (2.11, 2.88)	2.96 (2.31, 3.80)	2.57 (2.21, 3.00)	0.194
Aortic aneurysm	4,989	1,525	1.02 (0.99, 1.05)	1.03 (0.98, 1.08)	1.02 (0.99, 1.04)	1.31 (0.82, 2.09)	1.59 (0.68, 3.71)	1.37 (0.91, 2.07)	0.712
Stroke	21,988	14,475	1.04 (1.02, 1.05)	1.05 (1.03, 1.07)	1.04 (1.03, 1.05)	1.84 (1.46, 2.32)	2.18 (1.63, 2.91)	1.97 (1.62, 2.38)	0.396
Diabetes	4,258	2,594	1.06 (1.03, 1.09)	1.10 (1.06, 1.15)	1.08 (1.05, 1.10)	2.71 (1.62, 4.54)	5.08 (2.59, 9.96)	3.44 (2.24, 5.27)	0.150
Kidney disease	1,992	1,151	1.02 (0.97, 1.06)	1.12 (1.05, 1.18)	1.05 (1.02, 1.09)	1.36 (0.65, 2.84)	6.18 (2.29, 16.68)	2.36 (1.30, 4.28)	0.015
Respiratory diseases	13,996	7,826	1.00 (0.98, 1.02)	1.02 (1.00, 1.04)	1.01 (0.99, 1.02)	0.98 (0.74, 1.29)	1.34 (0.92, 1.95)	1.10 (0.88, 1.37)	0.170
External causes	25,465	9,384	1.00 (0.99, 1.02)	0.98 (0.96, 1.00)	1.00 (0.99, 1.01)	1.08 (0.88, 1.33)	0.70 (0.50, 0.98)	0.96 (0.81, 1.15)	0.029
Suicide	9,475	3,795	1.01 (0.99, 1.03)	0.97 (0.94, 1.00)	1.00 (0.98, 1.01)	1.14 (0.81, 1.59)	0.60 (0.35, 1.01)	0.95 (0.71, 1.26)	0.044
Cancer	79,137	61,973	1.00 (1.00, 1.01)	1.01 (1.00, 1.01)	1.01 (1.00, 1.01)	1.08 (0.96, 1.21)	1.12 (0.98, 1.27)	1.09 (1.00, 1.19)	0.637
Colorectal cancer	8,910	6,644	1.01 (0.99, 1.03)	1.02 (1.00, 1.04)	1.01 (1.00, 1.03)	1.17 (0.83, 1.66)	1.38 (0.92, 2.07)	1.26 (0.97, 1.64)	0.536
Kidney cancer	3,484	1,962	1.02 (0.99, 1.06)	1.02 (0.97, 1.06)	1.02 (0.99, 1.05)	1.48 (0.85, 2.58)	1.32 (0.63, 2.78)	1.42 (0.91, 2.22)	0.818
Lung cancer	14,576	7,521	0.99 (0.98, 1.01)	0.98 (0.95, 1.00)	0.99 (0.97, 1.00)	0.90 (0.68, 1.18)	0.66 (0.45, 0.96)	0.81 (0.65, 1.01)	0.198
Lymphatic cancer	8,179	5,123	1.00 (0.98, 1.03)	1.00 (0.97, 1.03)	1.00 (0.99, 1.02)	1.05 (0.73, 1.51)	1.02 (0.65, 1.62)	1.04 (0.78, 1.38)	0.922
Breast cancer		11,365		1.01 (0.99, 1.03)			1.16 (0.85, 1.58)		
Prostate cancer	12,682		1.01 (0.99, 1.03)			1.16 (0.86, 1.55)			

Supplementary Table S12. BMI-adjusted (cf. Supplementary Table S8) hazard ratios (HR) for parental mortality (i) per standard deviation (SD) of a son's systolic blood pressure (SBP) and (ii) per SD of own SBP, using son's SBP as an instrumental variable (IV). SBP was pre-adjusted for regional patterns, secular trends and age at examination and its SD was 10.80 mmHg. Cox proportional hazards models with age as the time axis were adjusted for parental sex, son's BMI and educational and occupational socioeconomic position. Robust standard errors were clustered by the son's identity. $P_{M\ vs\ F}$ was derived from a Z-test of an additional interaction term between parental sex and son's SBP. Two-sample IV estimates were made using the ratio method. Mothers and fathers were also modelled separately without the robust standard errors or the adjustment for parental sex. N = 1,002,031 mothers and 986,075 fathers at risk of mortality.

	Deaths		HR (95% CI) per SD of son's SBP			IV HR (95% CI) per SD of own SBP			
Cause of death	Fathers	Mothers	Fathers	Mothers	All parents	Fathers	Mothers	All parents	$P_{F\ vs\ M}$
All cause	281,212	152,429	1.01 (1.00, 1.01)	1.00 (1.00, 1.01)	1.01 (1.00, 1.01)	1.05 (1.02, 1.08)	1.03 (0.99, 1.07)	1.04 (1.02, 1.07)	0.426
Cardiovascular disease	127,017	50,823	1.04 (1.04, 1.05)	1.04 (1.03, 1.05)	1.04 (1.04, 1.05)	1.37 (1.31, 1.44)	1.36 (1.27, 1.45)	1.37 (1.32, 1.43)	0.965
Coronary heart disease	81,547	23,996	1.05 (1.05, 1.06)	1.06 (1.05, 1.07)	1.05 (1.05, 1.06)	1.49 (1.41, 1.58)	1.58 (1.43, 1.74)	1.52 (1.44, 1.60)	0.149
Aortic aneurysm	4,985	1,525	0.97 (0.95, 1.00)	0.96 (0.92, 1.01)	0.97 (0.95, 0.99)	0.80 (0.64, 0.99)	0.74 (0.50, 1.09)	0.79 (0.65, 0.95)	0.744
Stroke	21,965	14,463	1.03 (1.02, 1.04)	1.02 (1.00, 1.03)	1.02 (1.01, 1.03)	1.25 (1.12, 1.38)	1.13 (0.99, 1.28)	1.20 (1.11, 1.31)	0.204
Diabetes	4,252	2,588	1.04 (1.01, 1.07)	1.03 (0.99, 1.07)	1.03 (1.01, 1.06)	1.32 (1.05, 1.67)	1.22 (0.91, 1.65)	1.29 (1.08, 1.55)	0.747
Kidney disease	1,992	1,151	0.98 (0.94, 1.03)	1.01 (0.95, 1.07)	0.99 (0.96, 1.03)	0.88 (0.63, 1.24)	1.05 (0.67, 1.64)	0.95 (0.72, 1.24)	0.585
Respiratory diseases	13,987	7,821	0.97 (0.96, 0.99)	0.99 (0.97, 1.01)	0.98 (0.97, 0.99)	0.82 (0.72, 0.93)	0.93 (0.78, 1.10)	0.86 (0.77, 0.95)	0.276
External causes	25,411	9,373	0.96 (0.94, 0.97)	0.93 (0.91, 0.95)	0.95 (0.94, 0.96)	0.70 (0.63, 0.77)	0.55 (0.47, 0.64)	0.65 (0.60, 0.70)	0.021
Suicide	9,456	3,791	0.96 (0.94, 0.98)	0.92 (0.89, 0.95)	0.95 (0.93, 0.97)	0.73 (0.63, 0.86)	0.52 (0.41, 0.67)	0.66 (0.58, 0.76)	0.025
Cancer	79,069	61,906	0.99 (0.98, 0.99)	0.99 (0.99, 1.00)	0.99 (0.98, 0.99)	0.89 (0.85, 0.94)	0.95 (0.90, 1.01)	0.92 (0.88, 0.96)	0.366
Colorectal cancer	8,904	6,643	1.00 (0.98, 1.02)	1.00 (0.97, 1.02)	1.00 (0.98, 1.01)	0.99 (0.85, 1.17)	0.97 (0.80, 1.17)	0.98 (0.87, 1.11)	0.759
Kidney cancer	3,483	1,961	1.00 (0.97, 1.03)	1.06 (1.01, 1.10)	1.02 (0.99, 1.05)	0.99 (0.77, 1.29)	1.53 (1.09, 2.16)	1.17 (0.94, 1.44)	0.052
Lung cancer	14,565	7,512	0.96 (0.95, 0.98)	0.96 (0.94, 0.98)	0.96 (0.95, 0.97)	0.74 (0.65, 0.84)	0.73 (0.61, 0.87)	0.73 (0.66, 0.81)	0.805
Lymphatic cancer	8,173	5,121	1.00 (0.98, 1.02)	1.00 (0.97, 1.03)	1.00 (0.98, 1.02)	1.00 (0.84, 1.18)	1.01 (0.82, 1.25)	1.00 (0.88, 1.15)	0.986
Breast cancer		11,345		1.01 (0.99, 1.03)			1.08 (0.93, 1.25)		
Prostate cancer	12,673		1.00 (0.98, 1.01)			0.98 (0.85, 1.12)			

Supplementary Table S13. BMI-adjusted (cf. Supplementary Table S9) hazard ratios (HR) for parental mortality (i) per standard deviation (SD) of a son's diastolic blood pressure (DBP) and (ii) per SD of own DBP, using son's DBP as an instrumental variable (IV). DBP was pre-adjusted for regional patterns, secular trends and age at examination and its SD was 9.22 mmHg. Cox proportional hazards models with age as the time axis were adjusted for parental sex, son's BMI and educational and occupational socioeconomic position. Robust standard errors were clustered by the son's identity. $P_{M\ vs\ F}$ was derived from a Z-test of an additional interaction term between parental sex and son's DBP. Two-sample IV estimates were made using the ratio method. Mothers and fathers were also modelled separately without the robust standard errors or the adjustment for parental sex. N = 1,002,031 mothers and 986,075 fathers at risk of mortality.

	Deaths		HR (95% CI) per SD of son's DBP			IV HR (95% CI) per SD of own DBP			
Cause of death	Fathers	Mothers	Fathers	Mothers	All parents	Fathers	Mothers	All parents	$P_{F\ vs\ M}$
All cause	281,212	152,429	1.02 (1.01, 1.02)	1.01 (1.01, 1.02)	1.01 (1.01, 1.02)	1.31 (1.22, 1.41)	1.20 (1.10, 1.32)	1.27 (1.19, 1.35)	0.089
Cardiovascular disease	127,017	50,823	1.04 (1.03, 1.04)	1.04 (1.03, 1.05)	1.04 (1.03, 1.04)	1.80 (1.60, 2.02)	1.83 (1.55, 2.16)	1.81 (1.62, 2.02)	0.907
Coronary heart disease	81,547	23,996	1.04 (1.03, 1.05)	1.04 (1.03, 1.05)	1.04 (1.03, 1.05)	1.98 (1.72, 2.29)	1.94 (1.54, 2.45)	1.98 (1.73, 2.26)	0.887
Aortic aneurysm	4,985	1,525	1.01 (0.98, 1.04)	1.01 (0.96, 1.07)	1.01 (0.98, 1.03)	1.12 (0.69, 1.80)	1.25 (0.52, 2.97)	1.15 (0.76, 1.75)	0.831
Stroke	21,965	14,463	1.03 (1.02, 1.04)	1.03 (1.02, 1.05)	1.03 (1.02, 1.04)	1.65 (1.30, 2.08)	1.76 (1.32, 2.35)	1.69 (1.40, 2.05)	0.765
Diabetes	4,252	2,588	1.03 (1.00, 1.07)	1.04 (1.00, 1.08)	1.04 (1.01, 1.06)	1.79 (1.06, 3.01)	1.88 (0.97, 3.66)	1.84 (1.22, 2.78)	0.890
Kidney disease	1,992	1,151	1.01 (0.97, 1.06)	1.08 (1.02, 1.15)	1.04 (1.00, 1.08)	1.25 (0.59, 2.66)	3.69 (1.35, 10.07)	1.89 (1.03, 3.45)	0.092
Respiratory diseases	13,987	7,821	0.99 (0.98, 1.01)	1.00 (0.98, 1.02)	0.99 (0.98, 1.01)	0.87 (0.66, 1.16)	1.00 (0.68, 1.47)	0.92 (0.73, 1.15)	0.572
External causes	25,411	9,373	1.00 (0.99, 1.02)	0.97 (0.95, 0.99)	0.99 (0.98, 1.01)	1.07 (0.87, 1.32)	0.63 (0.45, 0.89)	0.92 (0.76, 1.10)	0.011
Suicide	9,456	3,791	1.01 (0.99, 1.03)	0.97 (0.94, 1.00)	1.00 (0.98, 1.01)	1.17 (0.83, 1.65)	0.58 (0.34, 0.99)	0.94 (0.71, 1.26)	0.030
Cancer	79,069	61,906	1.00 (0.99, 1.01)	1.00 (0.99, 1.00)	1.00 (0.99, 1.00)	0.99 (0.88, 1.12)	0.94 (0.82, 1.07)	0.97 (0.89, 1.06)	0.448
Colorectal cancer	8,904	6,643	1.00 (0.98, 1.02)	1.01 (0.99, 1.04)	1.01 (0.99, 1.02)	1.06 (0.74, 1.52)	1.25 (0.83, 1.90)	1.14 (0.87, 1.49)	0.563
Kidney cancer	3,483	1,961	1.02 (0.98, 1.05)	1.00 (0.95, 1.04)	1.01 (0.98, 1.04)	1.31 (0.74, 2.32)	0.95 (0.44, 2.03)	1.17 (0.74, 1.85)	0.503
Lung cancer	14,565	7,512	0.99 (0.97, 1.00)	0.96 (0.94, 0.98)	0.98 (0.96, 0.99)	0.81 (0.61, 1.07)	0.50 (0.34, 0.74)	0.68 (0.55, 0.86)	0.046
Lymphatic cancer	8,173	5,121	1.00 (0.98, 1.02)	0.99 (0.96, 1.02)	1.00 (0.98, 1.01)	0.99 (0.68, 1.44)	0.86 (0.54, 1.37)	0.93 (0.69, 1.24)	0.633
Breast cancer		11,345		1.01 (0.99, 1.03)			1.11 (0.81, 1.52)		
Prostate cancer	12,673		1.01 (0.99, 1.03)			1.15 (0.85, 1.56)			

Supplementary Table S14. Adjusted hazard ratios (HR) for parental <u>cancer</u> mortality (i) per standard deviation (SD) of a son's systolic blood pressure (<u>SBP</u>) and (ii) per SD of own SBP, using son's SBP as an instrumental variable (IV). SBP was pre-adjusted for regional patterns, secular trends and age at examination and its SD was 10.80 mmHg. Cox proportional hazards models with age as the time axis were adjusted for parental sex and for educational and occupational socioeconomic position. Robust standard errors were clustered by the son's identity. $P_{M\ vs\ F}$ was derived from a Z-test of an additional interaction term between parental sex and son's SBP. Two-sample IV estimates were made using the ratio method. Mothers and fathers were also modelled separately without the robust standard errors or the adjustment for parental sex. N = 1,002,031 mothers and 986,075 fathers at risk of mortality.

	Deaths		HR (95% CI) per SD of son's SBP			IV HR (95% CI) per SD of own SBP			
Cause of death	Fathers	Mothers	Fathers	Mothers	All parents	Fathers	Mothers	All parents	$P_{F\ vs\ M}$
Cancer	79,137	61,973	0.99 (0.98, 1.00)	1.00 (1.00, 1.01)	1.00 (0.99, 1.00)	0.94 (0.89, 0.99)	1.02 (0.97, 1.09)	0.98 (0.94, 1.02)	0.018
Bladder cancer	2,469	632	0.96 (0.92, 1.00)	1.00 (0.93, 1.08)	0.97 (0.94, 1.00)	0.74 (0.55, 0.99)	1.03 (0.58, 1.84)	0.79 (0.61, 1.03)	0.309
Brain cancer	3,072	2,232	1.01 (0.98, 1.05)	0.99 (0.95, 1.03)	1.00 (0.98, 1.03)	1.10 (0.84, 1.44)	0.90 (0.66, 1.23)	1.01 (0.83, 1.24)	0.329
Breast cancer		11,365		1.01 (0.99, 1.03)			1.08 (0.94, 1.24)		
Breast cancer, <50 y.o.		2,538		1.03 (0.99, 1.07)			1.22 (0.91, 1.64)		
Breast cancer, ≥50 y.o.		8,827		1.01 (0.99, 1.03)			1.09 (0.93, 1.27)		
Colorectal cancer	8,910	6,644	1.01 (0.99, 1.03)	1.00 (0.98, 1.02)	1.00 (0.99, 1.02)	1.06 (0.90, 1.24)	0.99 (0.83, 1.19)	1.03 (0.92, 1.16)	0.637
Gallbladder cancer	1,359	2,446	0.97 (0.92, 1.02)	1.01 (0.97, 1.05)	0.99 (0.96, 1.02)	0.78 (0.52, 1.17)	1.05 (0.78, 1.41)	0.95 (0.75, 1.20)	0.244
Kidney cancer	3,484	1,962	1.01 (0.98, 1.04)	1.07 (1.03, 1.12)	1.03 (1.00, 1.06)	1.07 (0.83, 1.37)	1.71 (1.23, 2.39)	1.27 (1.04, 1.55)	0.024
Liver cancer	2,217	1,356	0.99 (0.95, 1.04)	1.02 (0.97, 1.07)	1.00 (0.97, 1.04)	0.96 (0.70, 1.31)	1.15 (0.77, 1.71)	1.03 (0.81, 1.32)	0.480
Lung cancer	14,576	7,521	0.97 (0.95, 0.98)	0.98 (0.96, 1.00)	0.97 (0.96, 0.98)	0.77 (0.68, 0.87)	0.87 (0.73, 1.03)	0.80 (0.73, 0.89)	0.237
Lymphatic cancer	8,179	5,123	1.01 (0.98, 1.03)	1.01 (0.98, 1.04)	1.01 (0.99, 1.02)	1.04 (0.89, 1.23)	1.07 (0.87, 1.32)	1.06 (0.93, 1.20)	0.840
Malignant melanoma	1,852	1,092	1.01 (0.97, 1.06)	1.02 (0.96, 1.08)	1.02 (0.98, 1.05)	1.10 (0.78, 1.55)	1.16 (0.74, 1.81)	1.12 (0.86, 1.47)	0.862
Oesophageal cancer	1,721	429	0.95 (0.90, 0.99)	1.15 (1.05, 1.26)	0.99 (0.94, 1.03)	0.66 (0.46, 0.94)	2.93 (1.46, 5.89)	0.89 (0.65, 1.23)	<0.001
Ovarian cancer		4,959		1.00 (0.97, 1.03)			0.98 (0.80, 1.21)		
Prostate cancer	12,682		1.00 (0.98, 1.02)			0.99 (0.87, 1.13)			
Pancreatic cancer	5,259	4,201	0.99 (0.96, 1.02)	0.99 (0.96, 1.02)	0.99 (0.97, 1.01)	0.92 (0.75, 1.12)	0.90 (0.72, 1.13)	0.91 (0.78, 1.06)	0.889
Stomach cancer	4,908	2,448	1.01 (0.98, 1.04)	0.96 (0.92, 1.00)	0.99 (0.97, 1.02)	1.08 (0.88, 1.33)	0.74 (0.55, 1.00)	0.95 (0.80, 1.13)	0.043
Testicular cancer	205		0.91 (0.79, 1.05)			0.49 (0.17, 1.40)			
Thyroid cancer	222	272	1.07 (0.94, 1.21)	1.10 (0.98, 1.24)	1.09 (1.00, 1.18)	1.62 (0.61, 4.34)	2.10 (0.88, 5.05)	1.88 (1.01, 3.51)	0.688
Uterine cancer		3,509		0.99 (0.96, 1.02)			0.94 (0.73, 1.20)		
Cervical cancer		1,929		0.97 (0.93, 1.02)			0.80 (0.57, 1.12)		
Endometrial cancer		833		1.05 (0.98, 1.12)			1.44 (0.87, 2.40)		

Supplementary Table S15. Adjusted hazard ratios (HR) for parental cancer mortality (i) per standard deviation (SD) of a son's diastolic blood pressure (DBP) and (ii) per SD of own DBP, using son's DBP as an instrumental variable (IV). DBP was pre-adjusted for regional patterns, secular trends and age at examination and its SD was 9.22 mmHg. Cox proportional hazards models with age as the time axis were adjusted for parental sex and for educational and occupational socioeconomic position. Robust standard errors were clustered by the son's identity. $P_{M\ vs\ F}$ was derived from a Z-test of an additional interaction term between parental sex and son's DBP. Two-sample IV estimates were made using the ratio method. Mothers and fathers were also modelled separately without the robust standard errors or the adjustment for parental sex. N = 1,002,031 mothers and 986,075 fathers at risk of mortality.

	Deaths		HR (95% CI) per SD of son's DBP			IV HR (95% CI) per SD of own DBP			
Cause of death	Fathers	Mothers	Fathers	Mothers	All parents	Fathers	Mothers	All parents	$P_{F\ vs\ M}$
Cancer	79,137	61,973	1.00 (1.00, 1.01)	1.00 (0.99, 1.01)	1.00 (1.00, 1.01)	1.05 (0.94, 1.18)	1.04 (0.91, 1.18)	1.05 (0.96, 1.14)	0.815
Bladder cancer	2,469	632	1.01 (0.97, 1.05)	0.93 (0.86, 1.01)	0.99 (0.96, 1.03)	1.16 (0.60, 2.26)	0.30 (0.08, 1.12)	0.88 (0.49, 1.60)	0.071
Brain cancer	3,072	2,232	1.01 (0.97, 1.05)	1.00 (0.96, 1.05)	1.01 (0.98, 1.03)	1.16 (0.64, 2.11)	1.06 (0.53, 2.13)	1.11 (0.71, 1.72)	0.846
Breast cancer		11,365		1.01 (0.99, 1.03)			1.11 (0.81, 1.51)		
Breast cancer, <50 y.o.		2,538		1.01 (0.97, 1.04)			1.09 (0.57, 2.08)		
Breast cancer, ≥50 y.o.		8,827		1.01 (0.99, 1.03)			1.14 (0.80, 1.62)		
Colorectal cancer	8,910	6,644	1.01 (0.99, 1.03)	1.02 (0.99, 1.04)	1.01 (1.00, 1.03)	1.16 (0.82, 1.65)	1.29 (0.86, 1.94)	1.21 (0.93, 1.58)	0.653
Gallbladder cancer	1,359	2,446	1.01 (0.96, 1.07)	0.99 (0.95, 1.03)	0.99 (0.96, 1.03)	1.18 (0.48, 2.90)	0.78 (0.40, 1.53)	0.91 (0.54, 1.54)	0.477
Kidney cancer	3,484	1,962	1.02 (0.99, 1.06)	1.01 (0.97, 1.06)	1.02 (0.99, 1.05)	1.44 (0.82, 2.52)	1.18 (0.56, 2.49)	1.34 (0.86, 2.09)	0.692
Liver cancer	2,217	1,356	1.04 (1.00, 1.09)	1.04 (0.99, 1.10)	1.04 (1.01, 1.08)	2.04 (1.00, 4.13)	2.05 (0.83, 5.06)	2.05 (1.17, 3.57)	0.964
Lung cancer	14,576	7,521	0.99 (0.97, 1.01)	0.97 (0.95, 1.00)	0.98 (0.97, 1.00)	0.85 (0.64, 1.11)	0.64 (0.44, 0.94)	0.77 (0.61, 0.96)	0.286
Lymphatic cancer	8,179	5,123	1.00 (0.98, 1.03)	1.00 (0.97, 1.02)	1.00 (0.98, 1.02)	1.06 (0.73, 1.52)	0.94 (0.59, 1.49)	1.00 (0.75, 1.33)	0.718
Malignant melanoma	1,852	1,092	1.05 (1.00, 1.10)	1.06 (0.99, 1.12)	1.05 (1.01, 1.09)	2.15 (0.99, 4.64)	2.49 (0.91, 6.77)	2.26 (1.22, 4.18)	0.792
Oesophageal cancer	1,721	429	0.95 (0.91, 1.00)	1.09 (0.99, 1.20)	0.98 (0.94, 1.02)	0.43 (0.20, 0.95)	4.38 (0.88, 21.9)	0.68 (0.33, 1.41)	0.013
Ovarian cancer		4,959		0.98 (0.96, 1.01)			0.76 (0.48, 1.22)		
Prostate cancer	12,682		1.01 (0.99, 1.03)			1.16 (0.86, 1.56)			
Pancreatic cancer	5,259	4,201	1.00 (0.97, 1.03)	1.00 (0.97, 1.03)	1.00 (0.98, 1.02)	0.98 (0.62, 1.54)	1.00 (0.60, 1.66)	0.98 (0.70, 1.38)	0.957
Stomach cancer	4,908	2,448	0.99 (0.97, 1.02)	1.01 (0.97, 1.05)	1.00 (0.98, 1.02)	0.92 (0.57, 1.47)	1.19 (0.61, 2.33)	1.00 (0.68, 1.47)	0.501
Testicular cancer	205		1.06 (0.93, 1.22)			2.83 (0.29, 27.8)			
Thyroid cancer	222	272	0.98 (0.86, 1.12)	1.12 (0.99, 1.26)	1.05 (0.97, 1.15)	0.74 (0.08, 6.73)	6.18 (0.82, 46.3)	2.34 (0.57, 9.67)	0.147
Uterine cancer		3,509		1.02 (0.99, 1.06)			1.42 (0.81, 2.48)		
Cervical cancer		1,929		0.99 (0.95, 1.04)			0.85 (0.40, 1.80)		
Endometrial cancer		833		1.04 (0.97, 1.11)			1.86 (0.59, 5.86)		

Supplementary Table S16. Unadjusted (cf. Table 2) hazard ratios (HR) for paternal mortality (i) per standard deviation (SD) of own systolic blood pressure (SBP) and (ii) per SD of own SBP, using son's SBP as an instrumental variable (IV) within the subset having data on own SBP. SBP was pre-adjusted for regional patterns, secular trends and age at examination and its SD was 10.80 mmHg. Cox proportional hazards models with age as the time axis were not further adjusted. One-sample IV estimates were made using the ratio method. $P_{own\ vs\ IV}$ was derived from Durbin-Wu-Hausman test comparing the two HR. N = 66,567 fathers at risk of mortality. Rarer causes of death (<50 deaths in the data subset) are omitted.

Cause of death	Deaths	HR (95% CI) per SD of own SBP	IV HR (95% CI) per SD of own SBP	$P_{own\ vs\ IV}$
All cause	2,332	1.02 (0.98, 1.06)	0.84 (0.62, 1.15)	0.242
Cardiovascular disease	423	1.21 (1.10, 1.32)	1.19 (0.57, 2.48)	0.975
Coronary heart disease	235	1.22 (1.09, 1.38)	1.75 (0.65, 4.67)	0.475
Stroke	86	1.20 (0.99, 1.47)	1.69 (0.33, 8.56)	0.681
External causes	1,065	0.95 (0.90, 1.01)	0.75 (0.47, 1.19)	0.312
Suicide	466	0.93 (0.85, 1.02)	0.71 (0.35, 1.42)	0.436
Cancer	428	1.03 (0.94, 1.13)	0.91 (0.44, 1.89)	0.737
Brain cancer	61	1.12 (0.88, 1.43)	0.29 (0.04, 2.00)	0.167
Lung cancer	59	0.84 (0.65, 1.09)	1.15 (0.16, 8.15)	0.756
Lymphatic cancer	64	1.01 (0.79, 1.28)	0.30 (0.05, 1.95)	0.200

Supplementary Table S17. Unadjusted (cf. Table 3) hazard ratios (HR) for paternal mortality (i) per standard deviation (SD) of own diastolic blood pressure (DBP) and (ii) per SD of own DBP, using son's DBP as an instrumental variable (IV) within the subset having data on own DBP. DBP was pre-adjusted for regional patterns, secular trends and age at examination and its SD was 9.22 mmHg. Cox proportional hazards models with age as the time axis were not further adjusted. One-sample IV estimates were made using the ratio method. $P_{own\ vs\ IV}$ was derived from Durbin-Wu-Hausman test comparing the two HR. N = 66,567 fathers at risk of mortality. Rarer causes of death (<50 deaths in the data subset) are omitted.

Cause of death	Deaths	HR (95% CI) per SD of own DBP	IV HR (95% CI) per SD of own DBP	$P_{own\ vs\ IV}$
All cause	2,332	1.00 (0.96, 1.05)	0.66 (0.34, 1.28)	0.218
Cardiovascular disease	423	1.10 (1.00, 1.22)	1.23 (0.26, 5.94)	0.889
Coronary heart disease	235	1.12 (0.98, 1.29)	2.76 (0.33, 22.87)	0.405
Stroke	86	1.14 (0.91, 1.42)	3.72 (0.11, 122.89)	0.506
External causes	1,065	0.96 (0.90, 1.02)	0.64 (0.24, 1.72)	0.424
Suicide	466	0.96 (0.87, 1.05)	0.81 (0.18, 3.60)	0.825
Cancer	428	1.02 (0.92, 1.13)	0.57 (0.12, 2.71)	0.463
Brain cancer	61	0.95 (0.73, 1.25)	0.25 (0.00, 15.76)	0.529
Lung cancer	59	0.84 (0.64, 1.10)	1.12 (0.02, 74.99)	0.894
Lymphatic cancer	64	0.94 (0.72, 1.22)	0.06 (0.00, 3.11)	0.169

Supplementary Table S18. Proportional hazards tests and adjusted age-specific hazard ratios (HR) for parental mortality per standard deviation (SD) of a son's systolic blood pressure (SBP). SBP was pre-adjusted for regional patterns, secular trends and age at examination and its SD was 10.80 mmHg. Cox proportional hazards models with age as the time axis were adjusted for educational and occupational socioeconomic position. Robust standard errors were clustered by the son's identity. P_{PH} was derived from a Pearson correlation of the scaled Schoenfeld residuals against age. Follow-up was then split at 60 years of age to estimate age-specific hazard ratios. N = 1,002,031 mothers and 986,075 fathers at risk of mortality.

			HR (95% CI) per SD of son's SBP			
	P_{PH}		<60 years old		>60 years old	
Cause of death	Fathers	Mothers	Fathers	Mothers	Fathers	Mothers
All cause	<0.001	<0.001	1.01 (1.00, 1.01)	0.99 (0.98, 1.00)	1.02 (1.01, 1.02)	1.02 (1.02, 1.03)
Cardiovascular disease	0.022	0.072	1.07 (1.06, 1.08)	1.08 (1.05, 1.10)	1.05 (1.04, 1.05)	1.05 (1.04, 1.06)
Coronary heart disease	0.007	0.018	1.09 (1.07, 1.10)	1.10 (1.06, 1.13)	1.06 (1.05, 1.07)	1.08 (1.06, 1.09)
Aortic aneurysm	0.532	0.980	1.01 (0.94, 1.09)	1.02 (0.89, 1.16)	0.98 (0.95, 1.01)	0.97 (0.92, 1.03)
Stroke	0.674	0.002	1.05 (1.02, 1.08)	1.09 (1.05, 1.13)	1.03 (1.02, 1.05)	1.01 (0.99, 1.02)
Diabetes	0.185	0.286	1.12 (1.06, 1.19)	1.11 (1.02, 1.21)	1.06 (1.02, 1.09)	1.08 (1.04, 1.13)
Kidney disease	0.041	0.317	0.95 (0.87, 1.04)	0.98 (0.89, 1.09)	1.00 (0.95, 1.05)	1.05 (0.99, 1.12)
Respiratory diseases	0.143	0.256	0.97 (0.92, 1.01)	0.99 (0.95, 1.05)	0.98 (0.96, 1.00)	1.01 (0.98, 1.03)
External causes	0.241	0.009	0.96 (0.94, 0.97)	0.91 (0.89, 0.93)	0.95 (0.93, 0.97)	0.96 (0.92, 0.99)
Suicide	0.060	0.953	0.96 (0.94, 0.99)	0.92 (0.89, 0.95)	0.94 (0.90, 0.98)	0.91 (0.84, 0.97)
Cancer	0.166	<0.001	1.00 (0.99, 1.01)	1.00 (0.99, 1.01)	0.99 (0.98, 1.00)	1.01 (1.00, 1.02)
Colorectal cancer	0.370	0.197	1.00 (0.96, 1.05)	0.98 (0.94, 1.02)	1.01 (0.98, 1.03)	1.01 (0.98, 1.04)
Kidney cancer	0.568	0.993	1.00 (0.94, 1.06)	1.09 (1.01, 1.18)	1.01 (0.97, 1.05)	1.06 (1.01, 1.12)
Lung cancer	0.245	0.156	0.97 (0.94, 1.00)	0.97 (0.93, 1.00)	0.97 (0.95, 0.98)	0.99 (0.96, 1.02)
Lymphatic cancer	0.048	0.517	1.00 (0.96, 1.04)	1.00 (0.95, 1.05)	1.01 (0.98, 1.03)	1.01 (0.98, 1.05)
Breast cancer		0.483		1.01 (0.98, 1.03)		1.02 (0.99, 1.04)
Prostate cancer	0.866		0.95 (0.88, 1.02)		1.00 (0.98, 1.02)	

Supplementary Table S19. Proportional hazards tests and adjusted age-specific hazard ratios (HR) for parental mortality per standard deviation (SD) of a son's diastolic blood pressure (DBP). DBP was pre-adjusted for regional patterns, secular trends and age at examination and its SD was 9.22 mmHg. Cox proportional hazards models with age as the time axis were adjusted for educational and occupational socioeconomic position. Robust standard errors were clustered by the son's identity. P_{PH} was derived from a Pearson correlation of the scaled Schoenfeld residuals against age. Follow-up was then split at 60 years of age to estimate age-specific hazard ratios. N = 1,002,031 mothers and 986,075 fathers at risk of mortality.

			HR (95% CI) per SD of son's DBP			
	P_{PH}		<60 years old		>60 years old	
Cause of death	Fathers	Mothers	Fathers	Mothers	Fathers	Mothers
All cause	0.880	<0.001	1.02 (1.02, 1.03)	1.01 (1.00, 1.02)	1.02 (1.02, 1.02)	1.02 (1.02, 1.03)
Cardiovascular disease	0.017	0.238	1.06 (1.05, 1.07)	1.07 (1.04, 1.09)	1.04 (1.03, 1.05)	1.04 (1.03, 1.05)
Coronary heart disease	0.050	0.100	1.07 (1.05, 1.08)	1.08 (1.04, 1.12)	1.05 (1.04, 1.05)	1.05 (1.04, 1.07)
Aortic aneurysm	0.691	0.272	1.06 (0.98, 1.13)	1.11 (0.97, 1.26)	1.01 (0.98, 1.04)	1.01 (0.96, 1.07)
Stroke	0.442	0.248	1.05 (1.01, 1.08)	1.06 (1.02, 1.10)	1.03 (1.02, 1.05)	1.03 (1.02, 1.05)
Diabetes	0.107	0.069	1.09 (1.02, 1.15)	1.13 (1.03, 1.23)	1.05 (1.01, 1.09)	1.08 (1.03, 1.12)
Kidney disease	0.558	0.209	1.01 (0.93, 1.10)	1.11 (1.00, 1.23)	1.02 (0.96, 1.07)	1.09 (1.02, 1.17)
Respiratory diseases	0.948	0.833	0.99 (0.94, 1.03)	1.02 (0.97, 1.07)	0.99 (0.98, 1.01)	1.01 (0.98, 1.03)
External causes	0.338	0.067	1.01 (0.99, 1.02)	0.97 (0.95, 0.99)	1.00 (0.97, 1.02)	0.97 (0.94, 1.01)
Suicide	0.449	0.174	1.01 (0.99, 1.04)	0.97 (0.93, 1.00)	0.99 (0.95, 1.03)	0.96 (0.89, 1.03)
Cancer	0.725	0.064	1.00 (0.99, 1.02)	1.00 (0.99, 1.01)	1.00 (0.99, 1.01)	1.00 (0.99, 1.01)
Colorectal cancer	0.827	0.374	0.99 (0.95, 1.04)	1.00 (0.96, 1.05)	1.01 (0.99, 1.04)	1.02 (0.99, 1.05)
Kidney cancer	0.777	0.981	1.03 (0.97, 1.10)	1.04 (0.96, 1.12)	1.02 (0.98, 1.06)	1.00 (0.94, 1.05)
Lung cancer	0.240	0.124	1.01 (0.98, 1.04)	0.96 (0.92, 0.99)	0.98 (0.97, 1.00)	0.98 (0.96, 1.01)
Lymphatic cancer	0.735	0.561	0.98 (0.94, 1.02)	0.98 (0.94, 1.02)	1.02 (0.99, 1.04)	1.01 (0.97, 1.04)
Breast cancer		0.424		1.01 (0.99, 1.04)		1.00 (0.97, 1.03)
Prostate cancer	0.078		1.00 (0.93, 1.07)		1.01 (0.99, 1.03)	

Supplementary Figure S1. Flow of participants through the study.

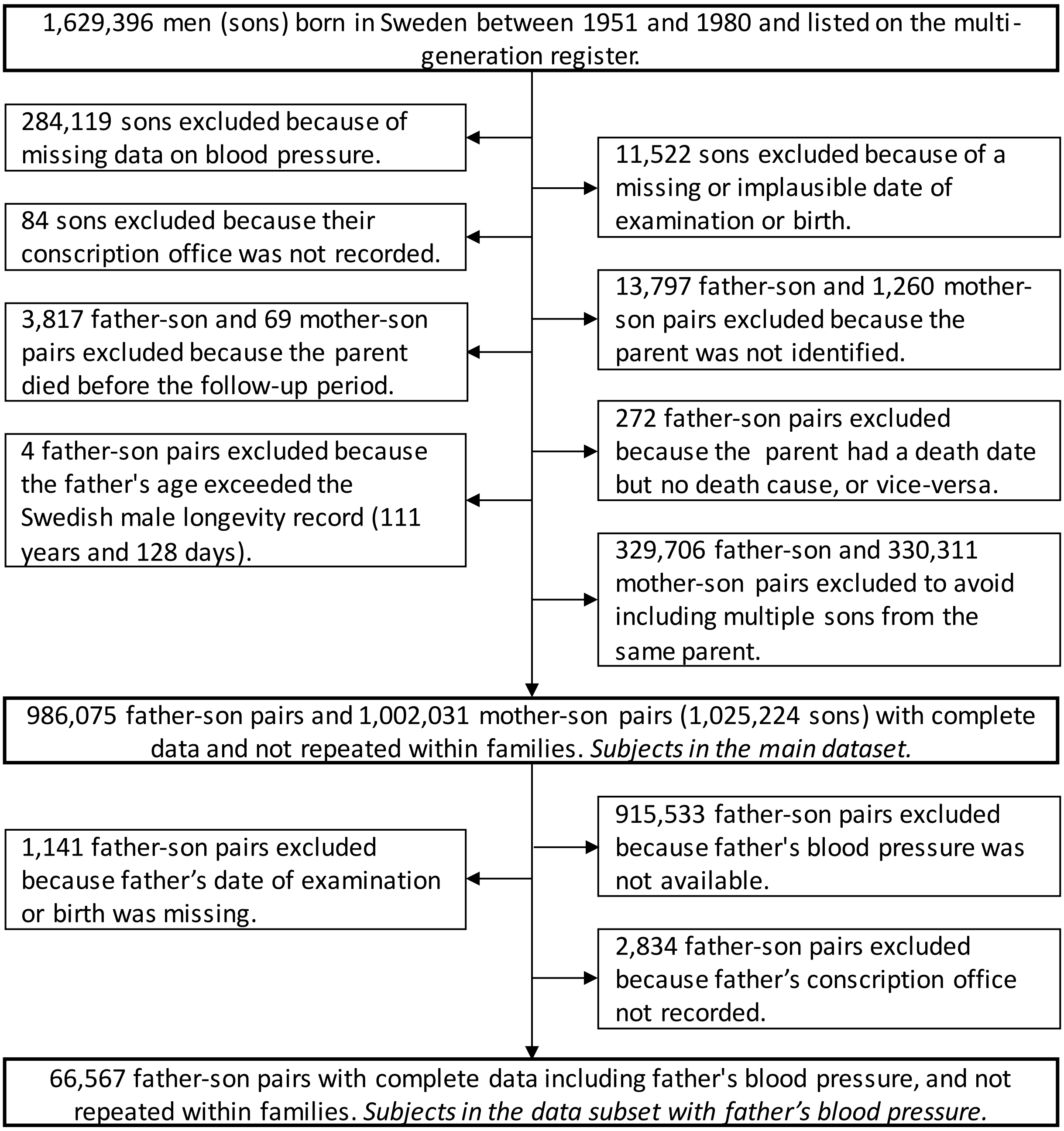

Supplementary Figure S2. Association of systolic (SBP) and diastolic (DBP) blood pressure between fathers and sons. Means and standard errors of father's SBP or DBP are plotted within centiles of son's SBP or DBP, using the subset with data on father's blood pressure (N=66,567). All blood pressure values were adjusted for regional patterns, secular trends and age at examination.

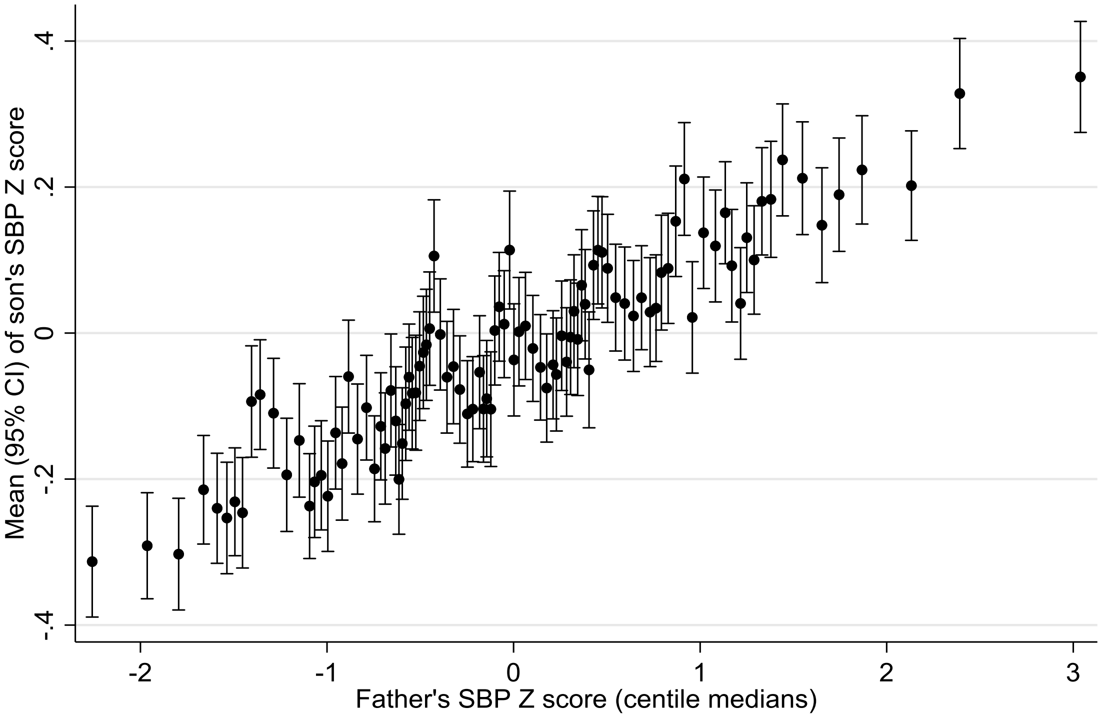

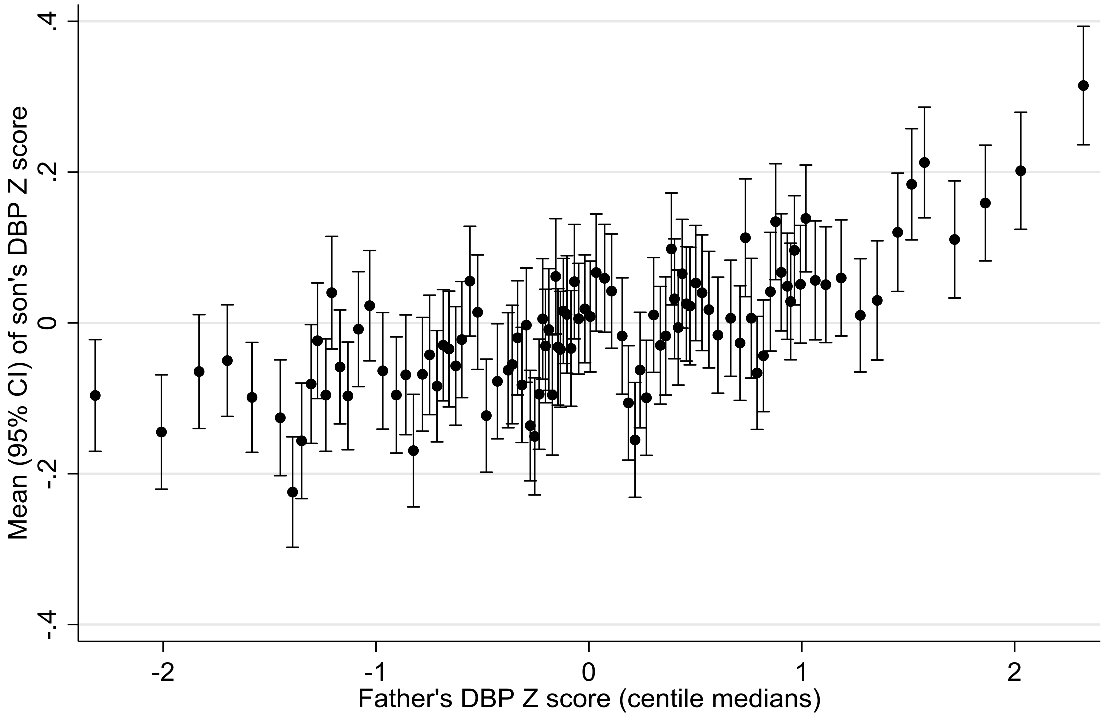

Supplementary Figure S3 (part 1 of 6). Plots of hazard ratio (relative to the median blood pressure) for parental mortality against a son's systolic (SBP) or diastolic (DBP) blood pressure. Son's SBP and DBP were pre-adjusted for regional patterns, secular trends and age at examination. Cox regressions with parental age as the time axis modeled SBP or DBP as cubic splines with 4 knots and were adjusted for the parent's educational and occupational socioeconomic position. Shaded areas represent 95% confidence intervals. For clarity, plots are truncated at the 1st and 99th percentiles of SBP or DBP and scaling of the vertical axis varies between causes of death.

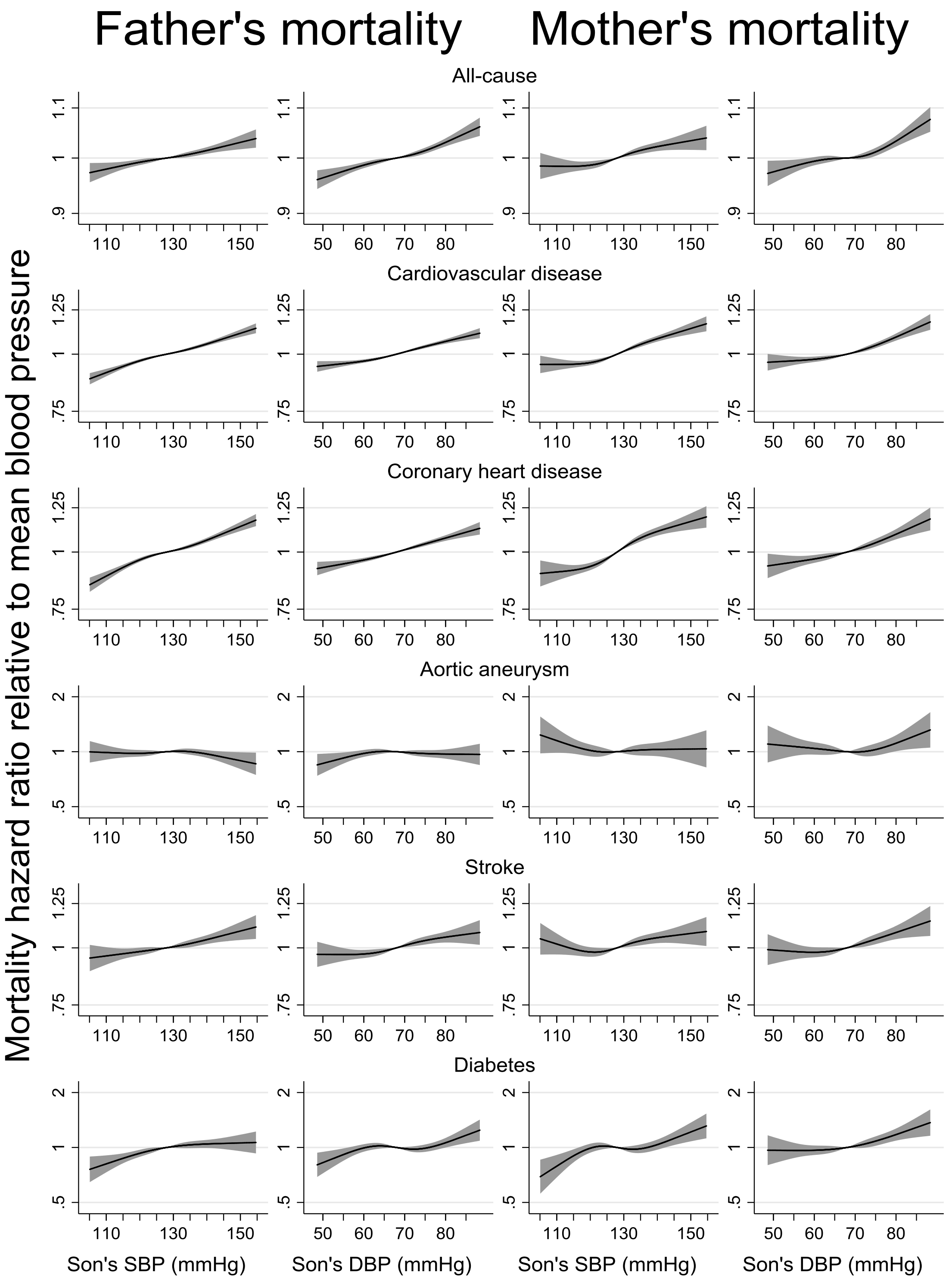

Supplementary Figure S3 (part 2 of 6).

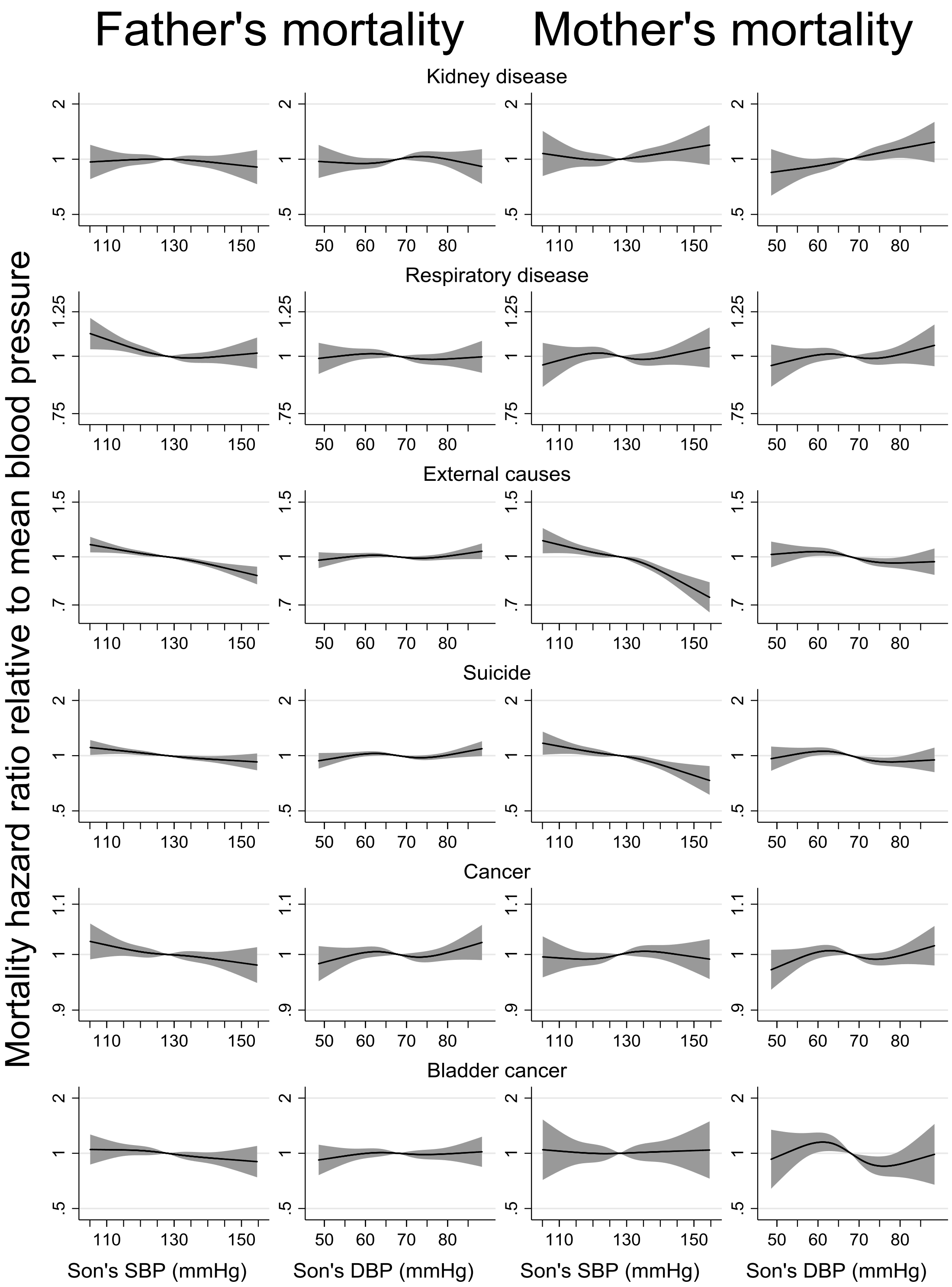

Supplementary Figure S3 (part 3 of 6).

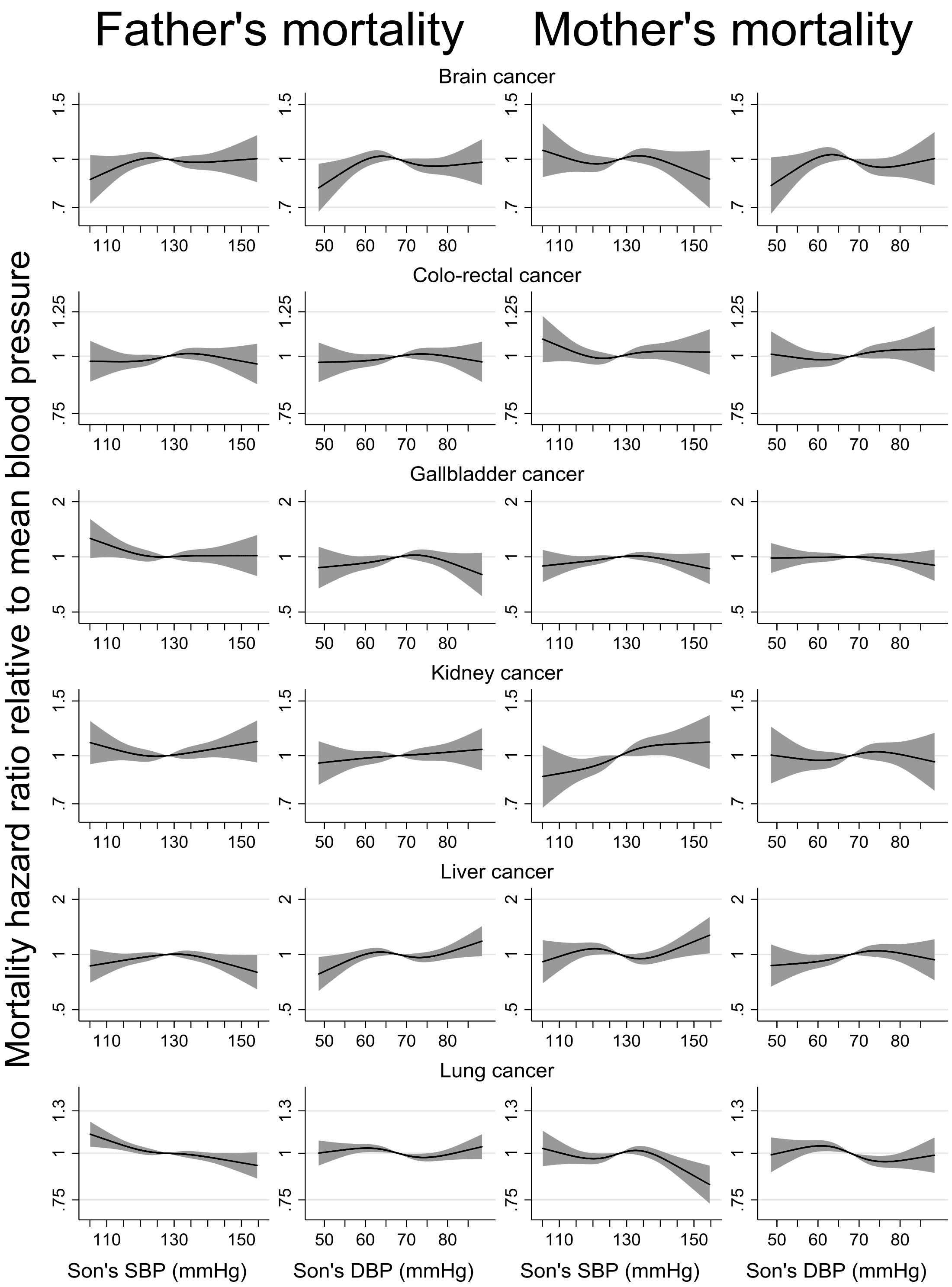

Supplementary Figure S3 (part 4 of 6).

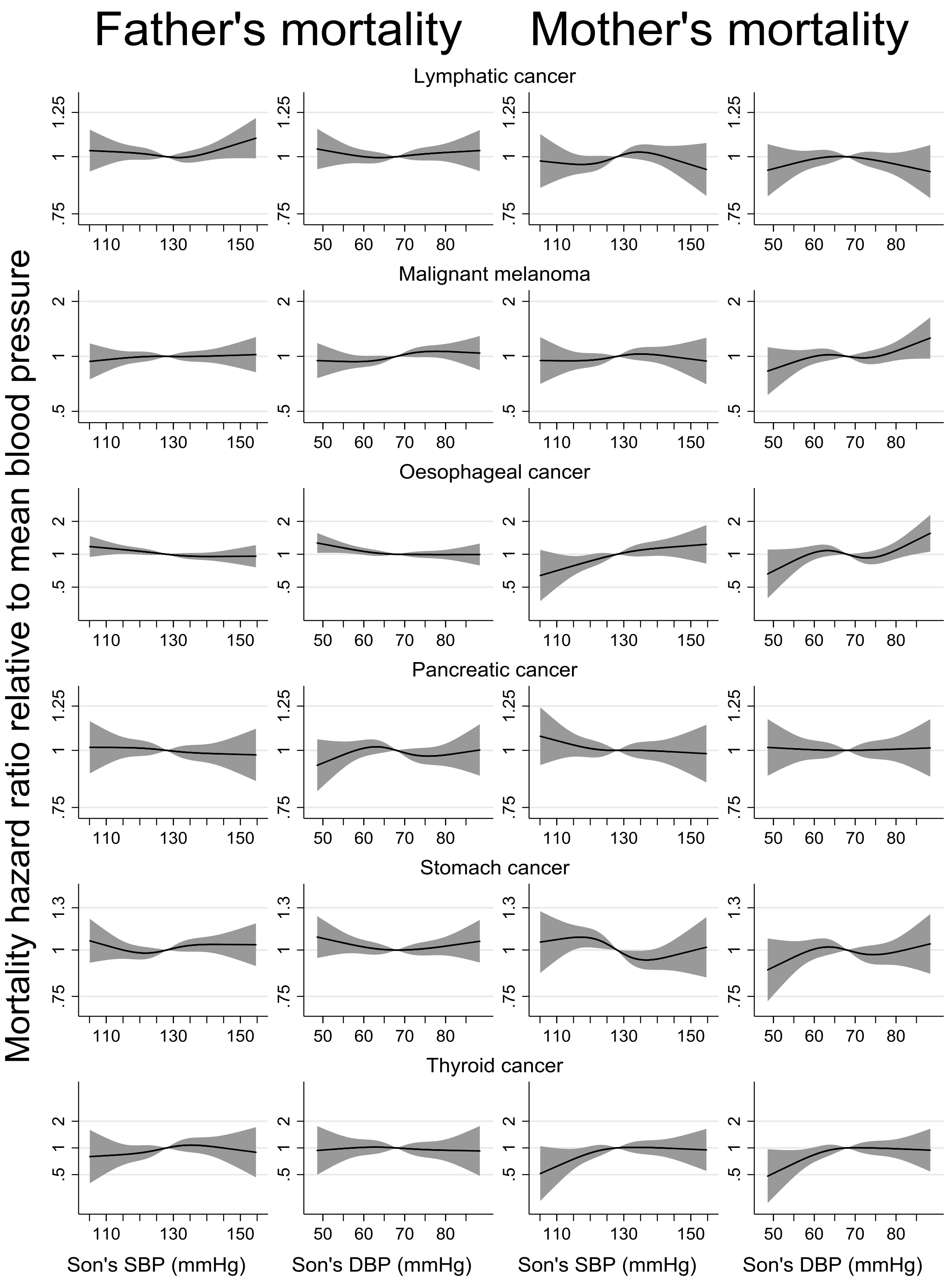

Supplementary Figure S3 (part 5 of 6).

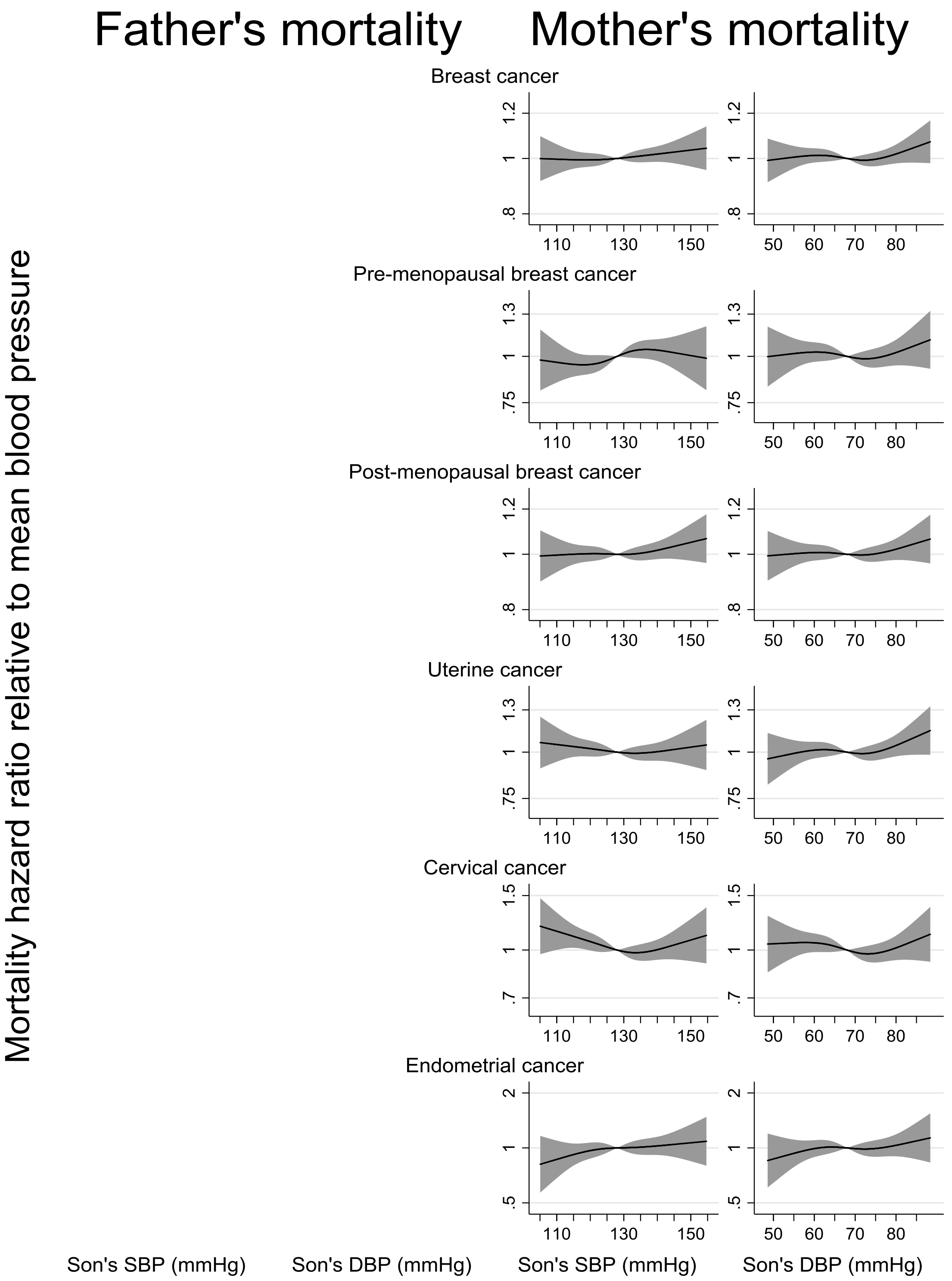

Supplementary Figure S3 (part 6 of 6).

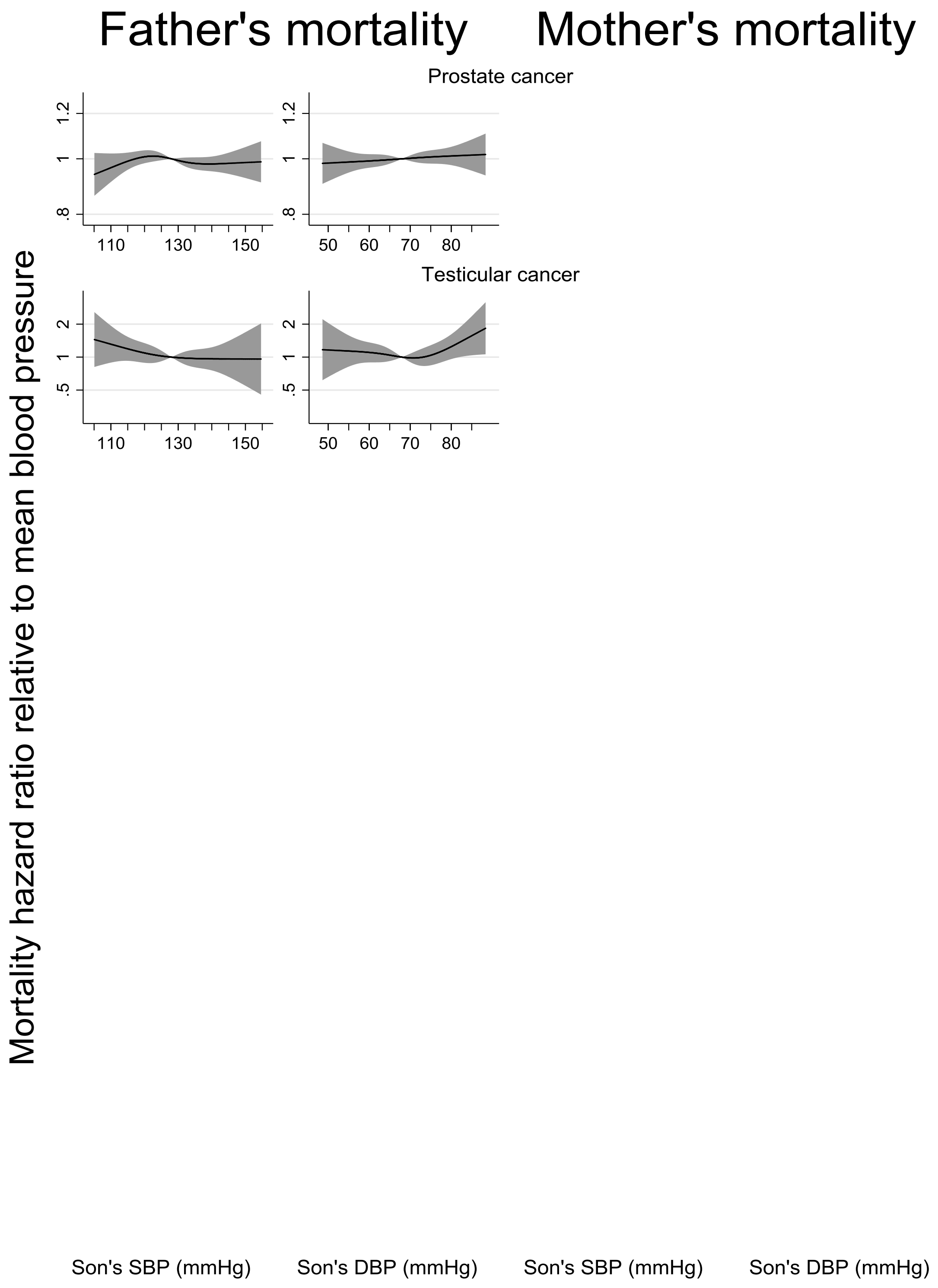

Supplement: Supplementary file 1 — Supplementary material [file 41598_2019_45391_MOESM1_ESM.pdf]
