# Supplementary material for: Author Correction: Associations of mortality with own blood pressure using son’s blood pressure as an instrumental variable
Source: Sci Rep. 2021 Mar 3;11:5470. doi: 10.1038/s41598-021-84494-1 (PMC7930109; doi:10.1038/s41598-021-84494-1)
Supplement: Supplementary file 1 — Supplementary Information 1. [file 41598_2021_84494_MOESM1_ESM.docx]

**Supplementary Table S8. Adjusted hazard ratios (HR) for parental mortality (i) per standard deviation (SD) of a son’s systolic blood pressure (SBP) and (ii) per SD of own SBP, using son’s SBP as an instrumental variable (IV).** SBP was pre-adjusted for regional patterns, secular trends and age at examination and its SD was 10.80 mmHg. Cox proportional hazards models with age as the time axis were adjusted for parental sex and for educational and occupational socioeconomic position. Robust standard errors were clustered by the son’s identity. P_M vs F_ was derived from a Z-test of an additional interaction term between parental sex and son’s SBP. Two-sample IV estimates were made using the ratio method. Mothers and fathers were also modelled separately without the robust standard errors or the adjustment for parental sex. N = 1,002,031 mothers and 986,075 fathers at risk of mortality.

|  | Deaths | |  | HR (95% CI) per SD of son's SBP | | |  | IV HR (95% CI) per SD of own SBP | | |  |  |
| --- | --- | --- | --- | --- | --- | --- | --- | --- | --- | --- | --- | --- |
| Cause of death | Fathers | Mothers |  | Fathers | Mothers | All parents |  | Fathers | Mothers | All parents |  | P_F vs M_ |
| All cause | 281,489 | 152,575 |  | 1.01 (1.01, 1.02) | 1.02 (1.01, 1.02) | 1.01 (1.01, 1.02) |  | 1.11 (1.07, 1.14) | 1.12 (1.08, 1.17) | 1.11 (1.09, 1.14) |  | 0.544 |
| Cardiovascular disease | 127,136 | 50,861 |  | 1.05 (1.05, 1.06) | 1.06 (1.05, 1.06) | 1.05 (1.05, 1.06) |  | 1.47 (1.41, 1.54) | 1.51 (1.41, 1.62) | 1.49 (1.43, 1.56) |  | 0.625 |
| Coronary heart disease | 81,628 | 24,009 |  | 1.06 (1.06, 1.07) | 1.08 (1.07, 1.09) | 1.07 (1.06, 1.08) |  | 1.61 (1.52, 1.71) | 1.81 (1.64, 2.00) | 1.66 (1.57, 1.75) |  | 0.041 |
| Aortic aneurysm | 4,989 | 1,525 |  | 0.98 (0.96, 1.01) | 0.98 (0.93, 1.03) | 0.98 (0.96, 1.01) |  | 0.89 (0.72, 1.09) | 0.85 (0.59, 1.24) | 0.88 (0.73, 1.05) |  | 0.851 |
| Stroke | 21,988 | 14,475 |  | 1.03 (1.02, 1.05) | 1.02 (1.01, 1.04) | 1.03 (1.02, 1.04) |  | 1.29 (1.17, 1.42) | 1.18 (1.05, 1.33) | 1.25 (1.16, 1.35) |  | 0.256 |
| Diabetes | 4,258 | 2,594 |  | 1.07 (1.04, 1.10) | 1.09 (1.05, 1.13) | 1.08 (1.05, 1.10) |  | 1.69 (1.35, 2.12) | 1.91 (1.44, 2.54) | 1.78 (1.49, 2.13) |  | 0.514 |
| Kidney disease | 1,992 | 1,151 |  | 0.99 (0.95, 1.03) | 1.03 (0.98, 1.09) | 1.00 (0.97, 1.04) |  | 0.90 (0.65, 1.26) | 1.27 (0.83, 1.95) | 1.03 (0.79, 1.34) |  | 0.216 |
| Respiratory diseases | 13,996 | 7,826 |  | 0.98 (0.96, 0.99) | 1.00 (0.98, 1.03) | 0.99 (0.97, 1.00) |  | 0.84 (0.74, 0.95) | 1.03 (0.87, 1.22) | 0.91 (0.82, 1.00) |  | 0.047 |
| External causes | 25,465 | 9,384 |  | 0.96 (0.94, 0.97) | 0.93 (0.91, 0.94) | 0.95 (0.94, 0.96) |  | 0.71 (0.64, 0.78) | 0.55 (0.47, 0.65) | 0.66 (0.61, 0.72) |  | 0.009 |
| Suicide | 9,475 | 3,795 |  | 0.96 (0.94, 0.98) | 0.92 (0.89, 0.94) | 0.95 (0.93, 0.96) |  | 0.73 (0.62, 0.85) | 0.51 (0.40, 0.65) | 0.65 (0.57, 0.74) |  | 0.014 |
| Cancer | 79,137 | 61,973 |  | 0.99 (0.98, 1.00) | 1.00 (1.00, 1.01) | 1.00 (0.99, 1.00) |  | 0.94 (0.89, 0.99) | 1.02 (0.97, 1.09) | 0.98 (0.94, 1.02) |  | 0.018 |
| Colorectal cancer | 8,910 | 6,644 |  | 1.01 (0.99, 1.03) | 1.00 (0.98, 1.02) | 1.00 (0.99, 1.02) |  | 1.06 (0.90, 1.24) | 0.99 (0.83, 1.19) | 1.03 (0.92, 1.16) |  | 0.637 |
| Kidney cancer | 3,484 | 1,962 |  | 1.01 (0.98, 1.04) | 1.07 (1.03, 1.12) | 1.03 (1.00, 1.06) |  | 1.07 (0.83, 1.37) | 1.71 (1.23, 2.39) | 1.27 (1.04, 1.55) |  | 0.024 |
| Lung cancer | 14,576 | 7,521 |  | 0.97 (0.95, 0.98) | 0.98 (0.96, 1.00) | 0.97 (0.96, 0.98) |  | 0.77 (0.68, 0.87) | 0.87 (0.73, 1.03) | 0.80 (0.73, 0.89) |  | 0.237 |
| Lymphatic cancer | 8,179 | 5,123 |  | 1.01 (0.98, 1.03) | 1.01 (0.98, 1.04) | 1.01 (0.99, 1.02) |  | 1.04 (0.89, 1.23) | 1.07 (0.87, 1.32) | 1.06 (0.93, 1.20) |  | 0.840 |
| Breast cancer |  | 11,365 |  |  | 1.01 (0.99, 1.03) |  |  |  | 1.08 (0.94, 1.24) |  |  |  |
| Prostate cancer | 12,682 |  |  | 1.00 (0.98, 1.02) |  |  |  | 0.99 (0.87, 1.13) |  |  |  |  |

**Supplementary Table S9. Adjusted hazard ratios (HR) for parental mortality (i) per standard deviation (SD) of a son’s diastolic blood pressure (DBP) and (ii) per SD of own DBP, using son’s DBP as an instrumental variable (IV).** DBP was pre-adjusted for regional patterns, secular trends and age at examination and its SD was 9.22 mmHg. Cox proportional hazards models with age as the time axis were adjusted for parental sex and for educational and occupational socioeconomic position. Robust standard errors were clustered by the son’s identity. P_M vs F_ was derived from a Z-test of an additional interaction term between parental sex and son’s DBP. Two-sample IV estimates were made using the ratio method. Mothers and fathers were also modelled separately without the robust standard errors or the adjustment for parental sex. N = 1,002,031 mothers and 986,075 fathers at risk of mortality.

|  | Deaths | |  | HR (95% CI) per SD of son's DBP | | |  | IV HR (95% CI) per SD of own DBP | | |  |  |
| --- | --- | --- | --- | --- | --- | --- | --- | --- | --- | --- | --- | --- |
| Cause of death | Fathers | Mothers |  | Fathers | Mothers | All parents |  | Fathers | Mothers | All parents |  | P_F vs M_ |
| All cause | 281,489 | 152,575 |  | 1.02 (1.02, 1.02) | 1.02 (1.01, 1.02) | 1.02 (1.02, 1.02) |  | 1.41 (1.31, 1.52) | 1.37 (1.24, 1.50) | 1.39 (1.30, 1.48) |  | 0.495 |
| Cardiovascular disease | 127,136 | 50,861 |  | 1.04 (1.04, 1.05) | 1.05 (1.04, 1.06) | 1.04 (1.04, 1.05) |  | 2.02 (1.78, 2.29) | 2.19 (1.84, 2.61) | 2.07 (1.84, 2.32) |  | 0.636 |
| Coronary heart disease | 81,628 | 24,009 |  | 1.05 (1.04, 1.06) | 1.06 (1.04, 1.07) | 1.05 (1.04, 1.06) |  | 2.26 (1.94, 2.63) | 2.48 (1.95, 3.15) | 2.30 (2.00, 2.66) |  | 0.727 |
| Aortic aneurysm | 4,989 | 1,525 |  | 1.01 (0.99, 1.04) | 1.02 (0.97, 1.08) | 1.02 (0.99, 1.04) |  | 1.27 (0.80, 2.03) | 1.50 (0.64, 3.52) | 1.33 (0.88, 2.00) |  | 0.762 |
| Stroke | 21,988 | 14,475 |  | 1.03 (1.02, 1.05) | 1.04 (1.02, 1.06) | 1.04 (1.02, 1.05) |  | 1.74 (1.38, 2.19) | 1.88 (1.42, 2.51) | 1.80 (1.49, 2.17) |  | 0.701 |
| Diabetes | 4,258 | 2,594 |  | 1.06 (1.03, 1.09) | 1.09 (1.04, 1.13) | 1.07 (1.04, 1.09) |  | 2.56 (1.52, 4.29) | 3.97 (2.03, 7.76) | 3.02 (1.98, 4.62) |  | 0.325 |
| Kidney disease | 1,992 | 1,151 |  | 1.01 (0.97, 1.06) | 1.10 (1.04, 1.17) | 1.05 (1.01, 1.08) |  | 1.27 (0.61, 2.67) | 4.86 (1.80, 13.10) | 2.09 (1.16, 3.79) |  | 0.030 |
| Respiratory diseases | 13,996 | 7,826 |  | 0.99 (0.98, 1.01) | 1.01 (0.99, 1.03) | 1.00 (0.99, 1.01) |  | 0.90 (0.68, 1.19) | 1.16 (0.80, 1.69) | 0.99 (0.79, 1.23) |  | 0.235 |
| External causes | 25,465 | 9,384 |  | 1.00 (0.99, 1.02) | 0.97 (0.95, 0.99) | 0.99 (0.98, 1.01) |  | 1.06 (0.87, 1.30) | 0.62 (0.44, 0.88) | 0.91 (0.76, 1.09) |  | 0.007 |
| Suicide | 9,475 | 3,795 |  | 1.01 (0.99, 1.03) | 0.96 (0.93, 1.00) | 0.99 (0.98, 1.01) |  | 1.14 (0.81, 1.59) | 0.54 (0.32, 0.93) | 0.91 (0.68, 1.21) |  | 0.021 |
| Cancer | 79,137 | 61,973 |  | 1.00 (1.00, 1.01) | 1.00 (0.99, 1.01) | 1.00 (1.00, 1.01) |  | 1.05 (0.94, 1.18) | 1.04 (0.91, 1.18) | 1.05 (0.96, 1.14) |  | 0.815 |
| Colorectal cancer | 8,910 | 6,644 |  | 1.01 (0.99, 1.03) | 1.02 (0.99, 1.04) | 1.01 (1.00, 1.03) |  | 1.16 (0.82, 1.65) | 1.29 (0.86, 1.94) | 1.21 (0.93, 1.58) |  | 0.653 |
| Kidney cancer | 3,484 | 1,962 |  | 1.02 (0.99, 1.06) | 1.01 (0.97, 1.06) | 1.02 (0.99, 1.05) |  | 1.44 (0.82, 2.52) | 1.18 (0.56, 2.49) | 1.34 (0.86, 2.09) |  | 0.692 |
| Lung cancer | 14,576 | 7,521 |  | 0.99 (0.97, 1.01) | 0.97 (0.95, 1.00) | 0.98 (0.97, 1.00) |  | 0.85 (0.64, 1.11) | 0.64 (0.44, 0.94) | 0.77 (0.61, 0.96) |  | 0.286 |
| Lymphatic cancer | 8,179 | 5,123 |  | 1.00 (0.98, 1.03) | 1.00 (0.97, 1.02) | 1.00 (0.98, 1.02) |  | 1.06 (0.73, 1.52) | 0.94 (0.59, 1.49) | 1.00 (0.75, 1.33) |  | 0.718 |
| Breast cancer |  | 11,365 |  |  | 1.01 (0.99, 1.03) |  |  |  | 1.11 (0.81, 1.51) |  |  |  |
| Prostate cancer | 12,682 |  |  | 1.01 (0.99, 1.03) |  |  |  | 1.16 (0.86, 1.56) |  |  |  |  |

**Supplementary Table S10. Unadjusted (cf. Supplementary Table S8) hazard ratios (HR) for parental mortality (i) per standard deviation (SD) of a son’s systolic blood pressure (SBP) and (ii) per SD of own SBP, using son’s SBP as an instrumental variable (IV).** SBP was pre-adjusted for regional patterns, secular trends and age at examination and its SD was 10.80 mmHg. Cox proportional hazards models with age as the time axis were adjusted for parental sex only. Robust standard errors were clustered by the son’s identity. P_M vs F_ was derived from a Z-test of an additional interaction term between parental sex and son’s SBP. Two-sample IV estimates were made using the ratio method. Mothers and fathers were also modelled separately without the robust standard errors or the adjustment for parental sex. N = 1,002,031 mothers and 986,075 fathers at risk of mortality.

|  | Deaths | |  | HR (95% CI) per SD of son's SBP | | |  | IV HR (95% CI) per SD of own SBP | | |  |  |
| --- | --- | --- | --- | --- | --- | --- | --- | --- | --- | --- | --- | --- |
| Cause of death | Fathers | Mothers |  | Fathers | Mothers | All parents |  | Fathers | Mothers | All parents |  | P_F vs M_ |
| All cause | 281,489 | 152,575 |  | 1.02 (1.02, 1.02) | 1.03 (1.02, 1.03) | 1.02 (1.02, 1.03) |  | 1.17 (1.14, 1.20) | 1.22 (1.17, 1.26) | 1.19 (1.16, 1.22) |  | 0.093 |
| Cardiovascular disease | 127,136 | 50,861 |  | 1.06 (1.06, 1.07) | 1.07 (1.06, 1.08) | 1.06 (1.06, 1.07) |  | 1.58 (1.50, 1.66) | 1.69 (1.58, 1.82) | 1.62 (1.54, 1.69) |  | 0.045 |
| Coronary heart disease | 81,628 | 24,009 |  | 1.07 (1.07, 1.08) | 1.10 (1.08, 1.11) | 1.08 (1.07, 1.09) |  | 1.74 (1.64, 1.85) | 2.05 (1.85, 2.27) | 1.81 (1.71, 1.92) |  | <0.001 |
| Aortic aneurysm | 4,989 | 1,525 |  | 0.99 (0.96, 1.01) | 0.98 (0.94, 1.03) | 0.99 (0.96, 1.01) |  | 0.90 (0.73, 1.12) | 0.89 (0.61, 1.30) | 0.90 (0.75, 1.08) |  | 0.943 |
| Stroke | 21,988 | 14,475 |  | 1.04 (1.03, 1.05) | 1.04 (1.02, 1.05) | 1.04 (1.03, 1.05) |  | 1.36 (1.23, 1.51) | 1.31 (1.16, 1.47) | 1.34 (1.24, 1.45) |  | 0.562 |
| Diabetes | 4,258 | 2,594 |  | 1.08 (1.05, 1.11) | 1.11 (1.07, 1.15) | 1.09 (1.07, 1.12) |  | 1.83 (1.46, 2.29) | 2.25 (1.69, 3.00) | 1.98 (1.66, 2.37) |  | 0.237 |
| Kidney disease | 1,992 | 1,151 |  | 1.00 (0.95, 1.04) | 1.05 (1.00, 1.12) | 1.02 (0.98, 1.05) |  | 0.97 (0.70, 1.35) | 1.51 (0.98, 2.33) | 1.14 (0.88, 1.49) |  | 0.115 |
| Respiratory diseases | 13,996 | 7,826 |  | 0.99 (0.97, 1.00) | 1.02 (0.99, 1.04) | 1.00 (0.98, 1.01) |  | 0.91 (0.80, 1.03) | 1.13 (0.96, 1.34) | 0.98 (0.89, 1.09) |  | 0.036 |
| External causes | 25,465 | 9,384 |  | 0.96 (0.94, 0.97) | 0.93 (0.91, 0.95) | 0.95 (0.94, 0.96) |  | 0.71 (0.64, 0.78) | 0.58 (0.49, 0.67) | 0.67 (0.61, 0.73) |  | 0.035 |
| Suicide | 9,475 | 3,795 |  | 0.96 (0.94, 0.98) | 0.92 (0.89, 0.95) | 0.94 (0.93, 0.96) |  | 0.71 (0.61, 0.83) | 0.51 (0.40, 0.65) | 0.65 (0.56, 0.74) |  | 0.027 |
| Cancer | 79,137 | 61,973 |  | 1.00 (0.99, 1.00) | 1.01 (1.00, 1.02) | 1.00 (1.00, 1.01) |  | 0.96 (0.91, 1.02) | 1.08 (1.01, 1.14) | 1.01 (0.97, 1.05) |  | 0.011 |
| Colorectal cancer | 8,910 | 6,644 |  | 1.01 (0.99, 1.03) | 1.01 (0.98, 1.03) | 1.01 (0.99, 1.02) |  | 1.08 (0.92, 1.26) | 1.04 (0.87, 1.25) | 1.06 (0.94, 1.20) |  | 0.771 |
| Kidney cancer | 3,484 | 1,962 |  | 1.01 (0.98, 1.05) | 1.08 (1.04, 1.13) | 1.04 (1.01, 1.07) |  | 1.10 (0.85, 1.41) | 1.86 (1.33, 2.59) | 1.33 (1.09, 1.63) |  | 0.014 |
| Lung cancer | 14,576 | 7,521 |  | 0.97 (0.96, 0.99) | 0.98 (0.96, 1.01) | 0.98 (0.96, 0.99) |  | 0.80 (0.71, 0.91) | 0.88 (0.74, 1.05) | 0.83 (0.75, 0.92) |  | 0.410 |
| Lymphatic cancer | 8,179 | 5,123 |  | 1.01 (0.99, 1.03) | 1.02 (0.99, 1.04) | 1.01 (1.00, 1.03) |  | 1.07 (0.91, 1.26) | 1.14 (0.92, 1.40) | 1.10 (0.96, 1.25) |  | 0.659 |
| Breast cancer |  | 11,365 |  |  | 1.01 (1.00, 1.03) |  |  |  | 1.12 (0.97, 1.28) |  |  |  |
| Prostate cancer | 12,682 |  |  | 1.00 (0.98, 1.02) |  |  |  | 0.99 (0.87, 1.13) |  |  |  |  |

**Supplementary Table S11. Unadjusted (cf. Supplementary Table S9) hazard ratios (HR) for parental mortality (i) per standard deviation (SD) of a son’s diastolic blood pressure (DBP) and (ii) per SD of own DBP, using son’s DBP as an instrumental variable (IV).** DBP was pre-adjusted for regional patterns, secular trends and age at examination and its SD was 9.22 mmHg. Cox proportional hazards models with age as the time axis were adjusted for parental sex only. Robust standard errors were clustered by the son’s identity. P_M vs F_ was derived from a Z-test of an additional interaction term between parental sex and son’s DBP. Two-sample IV estimates were made using the ratio method. Mothers and fathers were also modelled separately without the robust standard errors or the adjustment for parental sex. N = 1,002,031 mothers and 986,075 fathers at risk of mortality.

|  | Deaths | |  | HR (95% CI) per SD of son's DBP | | |  | IV HR (95% CI) per SD of own DBP | | |  |  |
| --- | --- | --- | --- | --- | --- | --- | --- | --- | --- | --- | --- | --- |
| Cause of death | Fathers | Mothers |  | Fathers | Mothers | All parents |  | Fathers | Mothers | All parents |  | P_F vs M_ |
| All cause | 281,489 | 152,575 |  | 1.02 (1.02, 1.03) | 1.03 (1.02, 1.03) | 1.02 (1.02, 1.03) |  | 1.49 (1.38, 1.61) | 1.53 (1.39, 1.69) | 1.50 (1.40, 1.61) |  | 0.617 |
| Cardiovascular disease | 127,136 | 50,861 |  | 1.05 (1.04, 1.05) | 1.06 (1.05, 1.07) | 1.05 (1.05, 1.06) |  | 2.18 (1.91, 2.48) | 2.58 (2.14, 3.09) | 2.29 (2.02, 2.60) |  | 0.101 |
| Coronary heart disease | 81,628 | 24,009 |  | 1.06 (1.05, 1.06) | 1.07 (1.05, 1.08) | 1.06 (1.05, 1.07) |  | 2.47 (2.11, 2.88) | 2.96 (2.31, 3.80) | 2.57 (2.21, 3.00) |  | 0.194 |
| Aortic aneurysm | 4,989 | 1,525 |  | 1.02 (0.99, 1.05) | 1.03 (0.98, 1.08) | 1.02 (0.99, 1.04) |  | 1.31 (0.82, 2.09) | 1.59 (0.68, 3.71) | 1.37 (0.91, 2.07) |  | 0.712 |
| Stroke | 21,988 | 14,475 |  | 1.04 (1.02, 1.05) | 1.05 (1.03, 1.07) | 1.04 (1.03, 1.05) |  | 1.84 (1.46, 2.32) | 2.18 (1.63, 2.91) | 1.97 (1.62, 2.38) |  | 0.396 |
| Diabetes | 4,258 | 2,594 |  | 1.06 (1.03, 1.09) | 1.10 (1.06, 1.15) | 1.08 (1.05, 1.10) |  | 2.71 (1.62, 4.54) | 5.08 (2.59, 9.96) | 3.44 (2.24, 5.27) |  | 0.150 |
| Kidney disease | 1,992 | 1,151 |  | 1.02 (0.97, 1.06) | 1.12 (1.05, 1.18) | 1.05 (1.02, 1.09) |  | 1.36 (0.65, 2.84) | 6.18 (2.29, 16.68) | 2.36 (1.30, 4.28) |  | 0.015 |
| Respiratory diseases | 13,996 | 7,826 |  | 1.00 (0.98, 1.02) | 1.02 (1.00, 1.04) | 1.01 (0.99, 1.02) |  | 0.98 (0.74, 1.29) | 1.34 (0.92, 1.95) | 1.10 (0.88, 1.37) |  | 0.170 |
| External causes | 25,465 | 9,384 |  | 1.00 (0.99, 1.02) | 0.98 (0.96, 1.00) | 1.00 (0.99, 1.01) |  | 1.08 (0.88, 1.33) | 0.70 (0.50, 0.98) | 0.96 (0.81, 1.15) |  | 0.029 |
| Suicide | 9,475 | 3,795 |  | 1.01 (0.99, 1.03) | 0.97 (0.94, 1.00) | 1.00 (0.98, 1.01) |  | 1.14 (0.81, 1.59) | 0.60 (0.35, 1.02) | 0.95 (0.71, 1.26) |  | 0.044 |
| Cancer | 79,137 | 61,973 |  | 1.00 (1.00, 1.01) | 1.01 (1.00, 1.01) | 1.01 (1.00, 1.01) |  | 1.08 (0.96, 1.21) | 1.12 (0.98, 1.27) | 1.09 (1.00, 1.19) |  | 0.637 |
| Colorectal cancer | 8,910 | 6,644 |  | 1.01 (0.99, 1.03) | 1.02 (1.00, 1.04) | 1.01 (1.00, 1.03) |  | 1.17 (0.83, 1.66) | 1.38 (0.92, 2.07) | 1.26 (0.97, 1.64) |  | 0.536 |
| Kidney cancer | 3,484 | 1,962 |  | 1.02 (0.99, 1.06) | 1.02 (0.97, 1.06) | 1.02 (0.99, 1.05) |  | 1.48 (0.85, 2.58) | 1.32 (0.63, 2.78) | 1.42 (0.91, 2.22) |  | 0.818 |
| Lung cancer | 14,576 | 7,521 |  | 0.99 (0.98, 1.01) | 0.98 (0.95, 1.00) | 0.99 (0.97, 1.00) |  | 0.90 (0.68, 1.18) | 0.66 (0.45, 0.97) | 0.81 (0.65, 1.01) |  | 0.198 |
| Lymphatic cancer | 8,179 | 5,123 |  | 1.00 (0.98, 1.03) | 1.00 (0.97, 1.03) | 1.00 (0.99, 1.02) |  | 1.05 (0.73, 1.51) | 1.02 (0.65, 1.62) | 1.04 (0.78, 1.38) |  | 0.922 |
| Breast cancer |  | 11,365 |  |  | 1.01 (0.99, 1.03) |  |  |  | 1.16 (0.85, 1.58) |  |  |  |
| Prostate cancer | 12,682 |  |  | 1.01 (0.99, 1.03) |  |  |  | 1.16 (0.86, 1.55) |  |  |  |  |

**Supplementary Table S12. BMI-adjusted (cf. Supplementary Table S8) hazard ratios (HR) for parental mortality (i) per standard deviation (SD) of a son’s systolic blood pressure (SBP) and (ii) per SD of own SBP, using son’s SBP as an instrumental variable (IV).** SBP was pre-adjusted for regional patterns, secular trends and age at examination and its SD was 10.80 mmHg. Cox proportional hazards models with age as the time axis were adjusted for parental sex, son’s BMI and educational and occupational socioeconomic position. Robust standard errors were clustered by the son’s identity. P_M vs F_ was derived from a Z-test of an additional interaction term between parental sex and son’s SBP. Two-sample IV estimates were made using the ratio method. Mothers and fathers were also modelled separately without the robust standard errors or the adjustment for parental sex. N = 1,002,031 mothers and 986,075 fathers at risk of mortality.

|  | Deaths | |  | HR (95% CI) per SD of son's SBP | | |  | IV HR (95% CI) per SD of own SBP | | |  |  |
| --- | --- | --- | --- | --- | --- | --- | --- | --- | --- | --- | --- | --- |
| Cause of death | Fathers | Mothers |  | Fathers | Mothers | All parents |  | Fathers | Mothers | All parents |  | P_F vs M_ |
| All cause | 281,212 | 152,429 |  | 1.01 (1.00, 1.01) | 1.00 (1.00, 1.01) | 1.01 (1.00, 1.01) |  | 1.05 (1.02, 1.08) | 1.03 (0.99, 1.07) | 1.04 (1.02, 1.07) |  | 0.426 |
| Cardiovascular disease | 127,017 | 50,823 |  | 1.04 (1.04, 1.05) | 1.04 (1.03, 1.05) | 1.04 (1.04, 1.05) |  | 1.37 (1.31, 1.44) | 1.36 (1.27, 1.45) | 1.37 (1.32, 1.43) |  | 0.965 |
| Coronary heart disease | 81,547 | 23,996 |  | 1.05 (1.05, 1.06) | 1.06 (1.05, 1.07) | 1.05 (1.05, 1.06) |  | 1.49 (1.41, 1.58) | 1.58 (1.43, 1.74) | 1.52 (1.44, 1.60) |  | 0.149 |
| Aortic aneurysm | 4,985 | 1,525 |  | 0.97 (0.95, 1.00) | 0.96 (0.92, 1.01) | 0.97 (0.95, 0.99) |  | 0.80 (0.64, 0.99) | 0.74 (0.50, 1.10) | 0.79 (0.65, 0.95) |  | 0.744 |
| Stroke | 21,965 | 14,463 |  | 1.03 (1.02, 1.04) | 1.02 (1.00, 1.03) | 1.02 (1.01, 1.03) |  | 1.25 (1.12, 1.38) | 1.13 (0.99, 1.28) | 1.20 (1.11, 1.31) |  | 0.204 |
| Diabetes | 4,252 | 2,588 |  | 1.04 (1.01, 1.07) | 1.03 (0.99, 1.07) | 1.03 (1.01, 1.06) |  | 1.32 (1.05, 1.67) | 1.22 (0.91, 1.65) | 1.29 (1.08, 1.55) |  | 0.747 |
| Kidney disease | 1,992 | 1,151 |  | 0.98 (0.94, 1.03) | 1.01 (0.95, 1.07) | 0.99 (0.96, 1.03) |  | 0.88 (0.63, 1.24) | 1.05 (0.67, 1.64) | 0.95 (0.72, 1.24) |  | 0.585 |
| Respiratory diseases | 13,987 | 7,821 |  | 0.97 (0.96, 0.99) | 0.99 (0.97, 1.01) | 0.98 (0.97, 0.99) |  | 0.82 (0.72, 0.93) | 0.93 (0.78, 1.10) | 0.86 (0.77, 0.95) |  | 0.276 |
| External causes | 25,411 | 9,373 |  | 0.96 (0.94, 0.97) | 0.93 (0.91, 0.95) | 0.95 (0.94, 0.96) |  | 0.70 (0.63, 0.77) | 0.55 (0.47, 0.65) | 0.65 (0.59, 0.71) |  | 0.021 |
| Suicide | 9,456 | 3,791 |  | 0.96 (0.94, 0.98) | 0.92 (0.89, 0.95) | 0.95 (0.93, 0.97) |  | 0.73 (0.62, 0.86) | 0.52 (0.41, 0.68) | 0.66 (0.58, 0.76) |  | 0.025 |
| Cancer | 79,069 | 61,906 |  | 0.99 (0.98, 0.99) | 0.99 (0.99, 1.00) | 0.99 (0.98, 0.99) |  | 0.89 (0.85, 0.94) | 0.95 (0.90, 1.01) | 0.92 (0.88, 0.96) |  | 0.366 |
| Colorectal cancer | 8,904 | 6,643 |  | 1.00 (0.98, 1.02) | 1.00 (0.97, 1.02) | 1.00 (0.98, 1.01) |  | 0.99 (0.85, 1.17) | 0.97 (0.80, 1.17) | 0.98 (0.87, 1.11) |  | 0.759 |
| Kidney cancer | 3,483 | 1,961 |  | 1.00 (0.97, 1.03) | 1.06 (1.01, 1.10) | 1.02 (0.99, 1.05) |  | 0.99 (0.77, 1.29) | 1.53 (1.09, 2.16) | 1.17 (0.94, 1.44) |  | 0.052 |
| Lung cancer | 14,565 | 7,512 |  | 0.96 (0.95, 0.98) | 0.96 (0.94, 0.98) | 0.96 (0.95, 0.97) |  | 0.74 (0.65, 0.84) | 0.73 (0.61, 0.87) | 0.73 (0.66, 0.82) |  | 0.805 |
| Lymphatic cancer | 8,173 | 5,121 |  | 1.00 (0.98, 1.02) | 1.00 (0.97, 1.03) | 1.00 (0.98, 1.02) |  | 1.00 (0.84, 1.18) | 1.01 (0.82, 1.25) | 1.00 (0.88, 1.15) |  | 0.986 |
| Breast cancer |  | 11,345 |  |  | 1.01 (0.99, 1.03) |  |  |  | 1.08 (0.93, 1.25) |  |  |  |
| Prostate cancer | 12,673 |  |  | 1.00 (0.98, 1.01) |  |  |  | 0.98 (0.85, 1.12) |  |  |  |  |

**Supplementary Table S13. BMI-adjusted (cf. Supplementary Table S9) hazard ratios (HR) for parental mortality (i) per standard deviation (SD) of a son’s diastolic blood pressure (DBP) and (ii) per SD of own DBP, using son’s DBP as an instrumental variable (IV).** DBP was pre-adjusted for regional patterns, secular trends and age at examination and its SD was 9.22 mmHg. Cox proportional hazards models with age as the time axis were adjusted for parental sex, son’s BMI and educational and occupational socioeconomic position. Robust standard errors were clustered by the son’s identity. P_M vs F_ was derived from a Z-test of an additional interaction term between parental sex and son’s DBP. Two-sample IV estimates were made using the ratio method. Mothers and fathers were also modelled separately without the robust standard errors or the adjustment for parental sex. N = 1,002,031 mothers and 986,075 fathers at risk of mortality.

|  | Deaths | |  | HR (95% CI) per SD of son's DBP | | |  | IV HR (95% CI) per SD of own DBP | | |  |  |
| --- | --- | --- | --- | --- | --- | --- | --- | --- | --- | --- | --- | --- |
| Cause of death | Fathers | Mothers |  | Fathers | Mothers | All parents |  | Fathers | Mothers | All parents |  | P_F vs M_ |
| All cause | 281,212 | 152,429 |  | 1.02 (1.01, 1.02) | 1.01 (1.01, 1.02) | 1.01 (1.01, 1.02) |  | 1.31 (1.22, 1.41) | 1.20 (1.10, 1.32) | 1.27 (1.19, 1.35) |  | 0.089 |
| Cardiovascular disease | 127,017 | 50,823 |  | 1.04 (1.03, 1.04) | 1.04 (1.03, 1.05) | 1.04 (1.03, 1.04) |  | 1.80 (1.60, 2.02) | 1.83 (1.55, 2.16) | 1.81 (1.62, 2.02) |  | 0.907 |
| Coronary heart disease | 81,547 | 23,996 |  | 1.04 (1.03, 1.05) | 1.04 (1.03, 1.05) | 1.04 (1.03, 1.05) |  | 1.98 (1.72, 2.29) | 1.94 (1.54, 2.45) | 1.98 (1.73, 2.26) |  | 0.887 |
| Aortic aneurysm | 4,985 | 1,525 |  | 1.01 (0.98, 1.04) | 1.01 (0.96, 1.07) | 1.01 (0.98, 1.03) |  | 1.12 (0.69, 1.80) | 1.25 (0.52, 2.97) | 1.15 (0.76, 1.75) |  | 0.831 |
| Stroke | 21,965 | 14,463 |  | 1.03 (1.02, 1.04) | 1.03 (1.02, 1.05) | 1.03 (1.02, 1.04) |  | 1.65 (1.30, 2.08) | 1.76 (1.32, 2.35) | 1.69 (1.40, 2.05) |  | 0.765 |
| Diabetes | 4,252 | 2,588 |  | 1.03 (1.00, 1.07) | 1.04 (1.00, 1.08) | 1.04 (1.01, 1.06) |  | 1.79 (1.06, 3.01) | 1.88 (0.97, 3.66) | 1.84 (1.22, 2.78) |  | 0.890 |
| Kidney disease | 1,992 | 1,151 |  | 1.01 (0.97, 1.06) | 1.08 (1.02, 1.15) | 1.04 (1.00, 1.08) |  | 1.25 (0.59, 2.66) | 3.69 (1.35, 10.07) | 1.89 (1.03, 3.45) |  | 0.092 |
| Respiratory diseases | 13,987 | 7,821 |  | 0.99 (0.98, 1.01) | 1.00 (0.98, 1.02) | 0.99 (0.98, 1.01) |  | 0.87 (0.65, 1.16) | 1.00 (0.68, 1.47) | 0.92 (0.73, 1.15) |  | 0.572 |
| External causes | 25,411 | 9,373 |  | 1.00 (0.99, 1.02) | 0.97 (0.95, 0.99) | 0.99 (0.98, 1.01) |  | 1.07 (0.87, 1.32) | 0.63 (0.44, 0.90) | 0.92 (0.76, 1.10) |  | 0.011 |
| Suicide | 9,456 | 3,791 |  | 1.01 (0.99, 1.03) | 0.97 (0.94, 1.00) | 1.00 (0.98, 1.01) |  | 1.17 (0.83, 1.65) | 0.58 (0.33, 1.00) | 0.94 (0.71, 1.26) |  | 0.030 |
| Cancer | 79,069 | 61,906 |  | 1.00 (0.99, 1.01) | 1.00 (0.99, 1.00) | 1.00 (0.99, 1.00) |  | 0.99 (0.88, 1.12) | 0.94 (0.82, 1.07) | 0.97 (0.89, 1.06) |  | 0.448 |
| Colorectal cancer | 8,904 | 6,643 |  | 1.00 (0.98, 1.02) | 1.01 (0.99, 1.04) | 1.01 (0.99, 1.02) |  | 1.06 (0.74, 1.52) | 1.25 (0.83, 1.90) | 1.14 (0.87, 1.49) |  | 0.563 |
| Kidney cancer | 3,483 | 1,961 |  | 1.02 (0.98, 1.05) | 1.00 (0.95, 1.04) | 1.01 (0.98, 1.04) |  | 1.31 (0.74, 2.32) | 0.95 (0.44, 2.03) | 1.17 (0.74, 1.85) |  | 0.503 |
| Lung cancer | 14,565 | 7,512 |  | 0.99 (0.97, 1.00) | 0.96 (0.94, 0.98) | 0.98 (0.96, 0.99) |  | 0.81 (0.61, 1.07) | 0.50 (0.34, 0.75) | 0.68 (0.54, 0.86) |  | 0.046 |
| Lymphatic cancer | 8,173 | 5,121 |  | 1.00 (0.98, 1.02) | 0.99 (0.96, 1.02) | 1.00 (0.98, 1.01) |  | 0.99 (0.68, 1.44) | 0.86 (0.54, 1.37) | 0.93 (0.69, 1.24) |  | 0.633 |
| Breast cancer |  | 11,345 |  |  | 1.01 (0.99, 1.03) |  |  |  | 1.11 (0.81, 1.52) |  |  |  |
| Prostate cancer | 12,673 |  |  | 1.01 (0.99, 1.03) |  |  |  | 1.15 (0.85, 1.56) |  |  |  |  |

**Supplementary Table S14. Adjusted hazard ratios (HR) for parental cancer mortality (i) per standard deviation (SD) of a son’s systolic blood pressure (SBP) and (ii) per SD of own SBP, using son’s SBP as an instrumental variable (IV).** SBP was pre-adjusted for regional patterns, secular trends and age at examination and its SD was 10.80 mmHg. Cox proportional hazards models with age as the time axis were adjusted for parental sex and for educational and occupational socioeconomic position. Robust standard errors were clustered by the son’s identity. P_M vs F_ was derived from a Z-test of an additional interaction term between parental sex and son’s SBP. Two-sample IV estimates were made using the ratio method. Mothers and fathers were also modelled separately without the robust standard errors or the adjustment for parental sex. N = 1,002,031 mothers and 986,075 fathers at risk of mortality.

|  | Deaths | |  | HR (95% CI) per SD of son's SBP | | |  | IV HR (95% CI) per SD of own SBP | | |  |  |
| --- | --- | --- | --- | --- | --- | --- | --- | --- | --- | --- | --- | --- |
| Cause of death | Fathers | Mothers |  | Fathers | Mothers | All parents |  | Fathers | Mothers | All parents |  | P_F vs M_ |
| Cancer | 79,137 | 61,973 |  | 0.99 (0.98, 1.00) | 1.00 (1.00, 1.01) | 1.00 (0.99, 1.00) |  | 0.94 (0.89, 0.99) | 1.02 (0.97, 1.09) | 0.98 (0.94, 1.02) |  | 0.018 |
| Bladder cancer | 2,469 | 632 |  | 0.96 (0.92, 1.00) | 1.00 (0.93, 1.08) | 0.97 (0.94, 1.00) |  | 0.74 (0.55, 0.99) | 1.03 (0.58, 1.84) | 0.79 (0.61, 1.03) |  | 0.309 |
| Brain cancer | 3,072 | 2,232 |  | 1.01 (0.98, 1.05) | 0.99 (0.95, 1.03) | 1.00 (0.98, 1.03) |  | 1.10 (0.84, 1.44) | 0.90 (0.66, 1.24) | 1.01 (0.83, 1.24) |  | 0.329 |
| Breast cancer |  | 11,365 |  |  | 1.01 (0.99, 1.03) |  |  |  | 1.08 (0.94, 1.24) |  |  |  |
| Breast cancer, <50 y.o. |  | 2,538 |  |  | 1.03 (0.99, 1.07) |  |  |  | 1.22 (0.91, 1.64) |  |  |  |
| Breast cancer, ≥50 y.o. |  | 8,827 |  |  | 1.01 (0.99, 1.03) |  |  |  | 1.09 (0.93, 1.27) |  |  |  |
| Colorectal cancer | 8,910 | 6,644 |  | 1.01 (0.99, 1.03) | 1.00 (0.98, 1.02) | 1.00 (0.99, 1.02) |  | 1.06 (0.90, 1.24) | 0.99 (0.83, 1.19) | 1.03 (0.92, 1.16) |  | 0.637 |
| Gallbladder cancer | 1,359 | 2,446 |  | 0.97 (0.92, 1.02) | 1.01 (0.97, 1.05) | 0.99 (0.96, 1.02) |  | 0.78 (0.52, 1.17) | 1.05 (0.78, 1.41) | 0.95 (0.75, 1.20) |  | 0.244 |
| Kidney cancer | 3,484 | 1,962 |  | 1.01 (0.98, 1.04) | 1.07 (1.03, 1.12) | 1.03 (1.00, 1.06) |  | 1.07 (0.83, 1.37) | 1.71 (1.23, 2.39) | 1.27 (1.04, 1.55) |  | 0.024 |
| Liver cancer | 2,217 | 1,356 |  | 0.99 (0.95, 1.04) | 1.02 (0.97, 1.07) | 1.00 (0.97, 1.04) |  | 0.96 (0.70, 1.31) | 1.15 (0.77, 1.71) | 1.03 (0.81, 1.32) |  | 0.480 |
| Lung cancer | 14,576 | 7,521 |  | 0.97 (0.95, 0.98) | 0.98 (0.96, 1.00) | 0.97 (0.96, 0.98) |  | 0.77 (0.68, 0.87) | 0.87 (0.73, 1.03) | 0.80 (0.73, 0.89) |  | 0.237 |
| Lymphatic cancer | 8,179 | 5,123 |  | 1.01 (0.98, 1.03) | 1.01 (0.98, 1.04) | 1.01 (0.99, 1.02) |  | 1.04 (0.89, 1.23) | 1.07 (0.87, 1.32) | 1.06 (0.93, 1.20) |  | 0.840 |
| Malignant melanoma | 1,852 | 1,092 |  | 1.01 (0.97, 1.06) | 1.02 (0.96, 1.08) | 1.02 (0.98, 1.05) |  | 1.10 (0.78, 1.55) | 1.16 (0.74, 1.81) | 1.12 (0.86, 1.47) |  | 0.862 |
| Oesophageal cancer | 1,721 | 429 |  | 0.95 (0.90, 0.99) | 1.15 (1.05, 1.26) | 0.99 (0.94, 1.03) |  | 0.66 (0.46, 0.94) | 2.93 (1.46, 5.89) | 0.89 (0.65, 1.23) |  | <0.001 |
| Ovarian cancer |  | 4,959 |  |  | 1.00 (0.97, 1.03) |  |  |  | 0.98 (0.80, 1.21) |  |  |  |
| Prostate cancer | 12,682 |  |  | 1.00 (0.98, 1.02) |  |  |  | 0.99 (0.87, 1.13) |  |  |  |  |
| Pancreatic cancer | 5,259 | 4,201 |  | 0.99 (0.96, 1.02) | 0.99 (0.96, 1.02) | 0.99 (0.97, 1.01) |  | 0.92 (0.75, 1.12) | 0.90 (0.72, 1.13) | 0.91 (0.78, 1.06) |  | 0.889 |
| Stomach cancer | 4,908 | 2,448 |  | 1.01 (0.98, 1.04) | 0.96 (0.92, 1.00) | 0.99 (0.97, 1.02) |  | 1.08 (0.88, 1.33) | 0.74 (0.55, 1.00) | 0.95 (0.80, 1.13) |  | 0.043 |
| Testicular cancer | 205 |  |  | 0.91 (0.79, 1.05) |  |  |  | 0.49 (0.17, 1.40) |  |  |  |  |
| Thyroid cancer | 222 | 272 |  | 1.07 (0.94, 1.21) | 1.10 (0.98, 1.24) | 1.09 (1.00, 1.18) |  | 1.62 (0.61, 4.34) | 2.10 (0.88, 5.05) | 1.88 (1.01, 3.51) |  | 0.688 |
| Uterine cancer |  | 3,509 |  |  | 0.99 (0.96, 1.02) |  |  |  | 0.94 (0.73, 1.20) |  |  |  |
| Cervical cancer |  | 1,929 |  |  | 0.97 (0.93, 1.02) |  |  |  | 0.80 (0.57, 1.12) |  |  |  |
| Endometrial cancer |  | 833 |  |  | 1.05 (0.98, 1.12) |  |  |  | 1.44 (0.87, 2.40) |  |  |  |

**Supplementary Table S15. Adjusted hazard ratios (HR) for parental cancer mortality (i) per standard deviation (SD) of a son’s diastolic blood pressure (DBP) and (ii) per SD of own DBP, using son’s DBP as an instrumental variable (IV).** DBP was pre-adjusted for regional patterns, secular trends and age at examination and its SD was 9.22 mmHg. Cox proportional hazards models with age as the time axis were adjusted for parental sex and for educational and occupational socioeconomic position. Robust standard errors were clustered by the son’s identity. P_M vs F_ was derived from a Z-test of an additional interaction term between parental sex and son’s DBP. Two-sample IV estimates were made using the ratio method. Mothers and fathers were also modelled separately without the robust standard errors or the adjustment for parental sex. N = 1,002,031 mothers and 986,075 fathers at risk of mortality.

|  | Deaths | |  | HR (95% CI) per SD of son's DBP | | |  | IV HR (95% CI) per SD of own DBP | | |  |  |
| --- | --- | --- | --- | --- | --- | --- | --- | --- | --- | --- | --- | --- |
| Cause of death | Fathers | Mothers |  | Fathers | Mothers | All parents |  | Fathers | Mothers | All parents |  | P_F vs M_ |
| Cancer | 79,137 | 61,973 |  | 1.00 (1.00, 1.01) | 1.00 (0.99, 1.01) | 1.00 (1.00, 1.01) |  | 1.05 (0.94, 1.18) | 1.04 (0.91, 1.18) | 1.05 (0.96, 1.14) |  | 0.815 |
| Bladder cancer | 2,469 | 632 |  | 1.01 (0.97, 1.05) | 0.93 (0.86, 1.01) | 0.99 (0.96, 1.03) |  | 1.16 (0.60, 2.26) | 0.30 (0.08, 1.13) | 0.88 (0.49, 1.60) |  | 0.071 |
| Brain cancer | 3,072 | 2,232 |  | 1.01 (0.97, 1.05) | 1.00 (0.96, 1.05) | 1.01 (0.98, 1.03) |  | 1.16 (0.64, 2.11) | 1.06 (0.53, 2.13) | 1.11 (0.71, 1.72) |  | 0.846 |
| Breast cancer |  | 11,365 |  |  | 1.01 (0.99, 1.03) |  |  |  | 1.11 (0.81, 1.51) |  |  |  |
| Breast cancer, <50 y.o. |  | 2,538 |  |  | 1.01 (0.97, 1.04) |  |  |  | 1.09 (0.57, 2.08) |  |  |  |
| Breast cancer, ≥50 y.o. |  | 8,827 |  |  | 1.01 (0.99, 1.03) |  |  |  | 1.14 (0.80, 1.62) |  |  |  |
| Colorectal cancer | 8,910 | 6,644 |  | 1.01 (0.99, 1.03) | 1.02 (0.99, 1.04) | 1.01 (1.00, 1.03) |  | 1.16 (0.82, 1.65) | 1.29 (0.86, 1.94) | 1.21 (0.93, 1.58) |  | 0.653 |
| Gallbladder cancer | 1,359 | 2,446 |  | 1.01 (0.96, 1.07) | 0.99 (0.95, 1.03) | 0.99 (0.96, 1.03) |  | 1.18 (0.48, 2.90) | 0.78 (0.40, 1.53) | 0.91 (0.54, 1.54) |  | 0.477 |
| Kidney cancer | 3,484 | 1,962 |  | 1.02 (0.99, 1.06) | 1.01 (0.97, 1.06) | 1.02 (0.99, 1.05) |  | 1.44 (0.82, 2.52) | 1.18 (0.56, 2.49) | 1.34 (0.86, 2.09) |  | 0.692 |
| Liver cancer | 2,217 | 1,356 |  | 1.04 (1.00, 1.09) | 1.04 (0.99, 1.10) | 1.04 (1.01, 1.08) |  | 2.04 (1.00, 4.13) | 2.05 (0.83, 5.06) | 2.05 (1.17, 3.57) |  | 0.964 |
| Lung cancer | 14,576 | 7,521 |  | 0.99 (0.97, 1.01) | 0.97 (0.95, 1.00) | 0.98 (0.97, 1.00) |  | 0.85 (0.64, 1.11) | 0.64 (0.44, 0.94) | 0.77 (0.61, 0.96) |  | 0.286 |
| Lymphatic cancer | 8,179 | 5,123 |  | 1.00 (0.98, 1.03) | 1.00 (0.97, 1.02) | 1.00 (0.98, 1.02) |  | 1.06 (0.73, 1.52) | 0.94 (0.59, 1.49) | 1.00 (0.75, 1.33) |  | 0.718 |
| Malignant melanoma | 1,852 | 1,092 |  | 1.05 (1.00, 1.10) | 1.06 (0.99, 1.12) | 1.05 (1.01, 1.09) |  | 2.15 (0.99, 4.64) | 2.49 (0.91, 6.77) | 2.26 (1.22, 4.18) |  | 0.792 |
| Oesophageal cancer | 1,721 | 429 |  | 0.95 (0.91, 1.00) | 1.09 (0.99, 1.20) | 0.98 (0.94, 1.02) |  | 0.43 (0.19, 0.97) | 4.38 (0.88, 21.9) | 0.68 (0.33, 1.42) |  | 0.013 |
| Ovarian cancer |  | 4,959 |  |  | 0.98 (0.96, 1.01) |  |  |  | 0.76 (0.48, 1.22) |  |  |  |
| Prostate cancer | 12,682 |  |  | 1.01 (0.99, 1.03) |  |  |  | 1.16 (0.86, 1.56) |  |  |  |  |
| Pancreatic cancer | 5,259 | 4,201 |  | 1.00 (0.97, 1.03) | 1.00 (0.97, 1.03) | 1.00 (0.98, 1.02) |  | 0.98 (0.62, 1.54) | 1.00 (0.60, 1.66) | 0.98 (0.70, 1.38) |  | 0.957 |
| Stomach cancer | 4,908 | 2,448 |  | 0.99 (0.97, 1.02) | 1.01 (0.97, 1.05) | 1.00 (0.98, 1.02) |  | 0.92 (0.57, 1.47) | 1.19 (0.61, 2.33) | 1.00 (0.68, 1.47) |  | 0.501 |
| Testicular cancer | 205 |  |  | 1.06 (0.93, 1.22) |  |  |  | 2.83 (0.29, 27.8) |  |  |  |  |
| Thyroid cancer | 222 | 272 |  | 0.98 (0.86, 1.12) | 1.12 (0.99, 1.26) | 1.05 (0.97, 1.15) |  | 0.74 (0.08, 6.73) | 6.18 (0.82, 46.3) | 2.34 (0.57, 9.67) |  | 0.147 |
| Uterine cancer |  | 3,509 |  |  | 1.02 (0.99, 1.06) |  |  |  | 1.42 (0.81, 2.48) |  |  |  |
| Cervical cancer |  | 1,929 |  |  | 0.99 (0.95, 1.04) |  |  |  | 0.85 (0.40, 1.80) |  |  |  |
| Endometrial cancer |  | 833 |  |  | 1.04 (0.97, 1.11) |  |  |  | 1.86 (0.59, 5.86) |  |  |  |

**Supplementary Table S16. Unadjusted (cf. Table 2) hazard ratios (HR) for paternal mortality (i) per standard deviation (SD) of own systolic blood pressure (SBP) and (ii) per SD of own SBP, using son’s SBP as an instrumental variable (IV) within the subset having data on own SBP.** SBP was pre-adjusted for regional patterns, secular trends and age at examination and its SD was 10.80 mmHg. Cox proportional hazards models with age as the time axis were not further adjusted. One-sample IV estimates were made using the ratio method. P_own vs IV_ was derived from Durbin-Wu-Hausman test comparing the two HR. N = 66,567 fathers at risk of mortality. Rarer causes of death (<50 deaths in the data subset) are omitted.

| Cause of death | Deaths | HR (95% CI) per SD of own SBP | IV HR (95% CI) per SD of own SBP | P_own vs IV_ |
| --- | --- | --- | --- | --- |
| All cause | 2,332 | 1.02 (0.98, 1.06) | 0.84 (0.62, 1.15) | 0.242 |
| Cardiovascular disease | 423 | 1.21 (1.10, 1.32) | 1.19 (0.57, 2.48) | 0.975 |
| Coronary heart disease | 235 | 1.22 (1.09, 1.38) | 1.75 (0.65, 4.67) | 0.475 |
| Stroke | 86 | 1.20 (0.99, 1.47) | 1.69 (0.33, 8.56) | 0.681 |
| External causes | 1,065 | 0.95 (0.90, 1.01) | 0.75 (0.47, 1.19) | 0.313 |
| Suicide | 466 | 0.93 (0.85, 1.02) | 0.71 (0.35, 1.42) | 0.436 |
| Cancer | 428 | 1.03 (0.94, 1.13) | 0.91 (0.44, 1.89) | 0.737 |
| Brain cancer | 61 | 1.12 (0.88, 1.43) | 0.29 (0.04, 2.01) | 0.167 |
| Lung cancer | 59 | 0.84 (0.65, 1.09) | 1.15 (0.16, 8.15) | 0.756 |
| Lymphatic cancer | 64 | 1.01 (0.79, 1.28) | 0.30 (0.05, 1.96) | 0.201 |

**Supplementary Table S17. Unadjusted (cf. Table 3) hazard ratios (HR) for paternal mortality (i) per standard deviation (SD) of own diastolic blood pressure (DBP) and (ii) per SD of own DBP, using son’s DBP as an instrumental variable (IV) within the subset having data on own DBP.** DBP was pre-adjusted for regional patterns, secular trends and age at examination and its SD was 9.22 mmHg. Cox proportional hazards models with age as the time axis were not further adjusted. One-sample IV estimates were made using the ratio method. P_own vs IV_ was derived from Durbin-Wu-Hausman test comparing the two HR. N = 66,567 fathers at risk of mortality. Rarer causes of death (<50 deaths in the data subset) are omitted.

| Cause of death | Deaths | HR (95% CI) per SD of own DBP | IV HR (95% CI) per SD of own DBP | P_own vs IV_ |
| --- | --- | --- | --- | --- |
| All cause | 2,332 | 1.00 (0.96, 1.05) | 0.66 (0.34, 1.29) | 0.220 |
| Cardiovascular disease | 423 | 1.10 (1.00, 1.22) | 1.23 (0.26, 5.94) | 0.889 |
| Coronary heart disease | 235 | 1.12 (0.98, 1.29) | 2.76 (0.33, 22.87) | 0.405 |
| Stroke | 86 | 1.14 (0.91, 1.42) | 3.72 (0.11, 122.89) | 0.506 |
| External causes | 1,065 | 0.96 (0.90, 1.02) | 0.64 (0.24, 1.72) | 0.425 |
| Suicide | 466 | 0.96 (0.87, 1.05) | 0.81 (0.18, 3.61) | 0.825 |
| Cancer | 428 | 1.02 (0.92, 1.13) | 0.57 (0.12, 2.71) | 0.464 |
| Brain cancer | 61 | 0.95 (0.73, 1.25) | 0.25 (0.00, 15.86) | 0.530 |
| Lung cancer | 59 | 0.84 (0.64, 1.10) | 1.12 (0.02, 74.99) | 0.894 |
| Lymphatic cancer | 64 | 0.94 (0.72, 1.22) | 0.06 (0.00, 3.20) | 0.172 |
